# Click chemistry towards thermally reversible photochromic 4,5-bisthiazolyl-1,2,3-triazoles

**DOI:** 10.3762/bjoc.15.213

**Published:** 2019-09-13

**Authors:** Chenxia Zhang, Kaori Morinaka, Mahmut Kose, Takashi Ubukata, Yasushi Yokoyama

**Affiliations:** 1 Department of Chemistry and Life Science, Graduate School of Engineering Science, Yokohama National University, 79-5, Tokiwadai, Hodogaya, Yokohama 240-8501, Japan; 2 Department of Chemistry, Faculty of Arts and Science, Zonguldak Bülent Ecevit University, 67100, Zonguldak, Turkey

**Keywords:** aromatic stabilization energy, diarylethene, ruthenium(I) catalysed Huisgen cyclization, terarylene, thermally reversible photochromism

## Abstract

Three new diarylethenes were synthesized from 1,2-bis(5-methyl-2-(4-substituted-phenyl)thiazol-4-yl)ethyne and benzyl azide through Ru(I)-catalyzed Huisgen cyclization reactions. The 4,5-bisthiazolyl-1,2,3-triazoles thus prepared, which belong to the terarylene family, showed thermally reversible photochromism. The absorption maximum wavelengths of the closed forms are longer than other terarylenes reported so far. The thermal back reactions are much faster when the substituents on the terminal phenyl groups are electron-withdrawing cyano groups than when they are electron-donating methoxy groups.

## Introduction

Diarylethenes are one of the most widely investigated among the photochromic families [1–3]. They are known to show thermally irreversible photochromism. However, some of them are thermally reversible [4]: (1) when the aromatic stabilization energy of the aromatic rings is large [5]; (2) when the substituent groups on the ring-closing carbon atoms are large [6]; (3) when the substituent groups on the ring-closing carbon atoms are strongly electron-withdrawing [7]; or (4) when the dialkylamino group on the side chain [8] or a carbon atom of the conjugation system in a strained closed form [9] are protonated.

Terarylenes [10–11], one of the closely related families of diarylethenes, are largely thermally reversible [10,12] although some are irreversible when the aromatic stabilization energy of the aromatic rings is small [11]. Their syntheses are usually carried out by the sequential construction of the central aromatic ring at the final stage [10] or the introduction of two aromatic rings to the central aromatic ring [11]. If the construction of three contiguous aromatic-ring arrays can be easily achieved, a new and facile synthesis method for the photochromic family which undergoes 6π-electrocyclization can be realized [13].

Early in the 21st century, Sharpless and co-workers proposed the concept of “click chemistry” [14], which stands for the secure, quick, selective, general and facile reaction between two organic functional groups. In click chemistry, the Huisgen cyclization, which occurs between an organic azide and a terminal alkyne catalyzed by a Cu(I) ion, was regarded as the representative reaction [15–16]. This reaction occurs even in aqueous media, the chemical yield is always high, and the regiochemical structure of the product is always 1,4-disubstituted triazole.

**Scheme 1:**
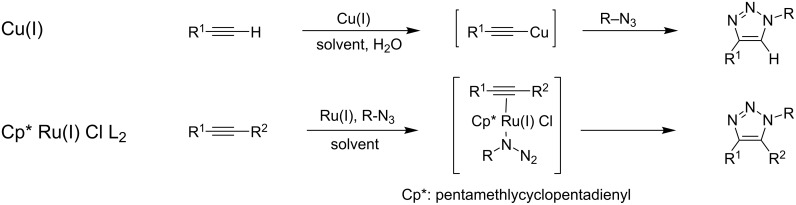
Reaction mechanisms of Huisgen cyclization catalyzed by Cu(I) and Ru(I).

Cu(I)-catalyzed Huisgen cyclization proceeds by the formation of copper acetylide as the intermediate [15,17], resulting in the formation of 1,4-disubstituted triazoles. In contrast, when Ru(I) complexes are employed as the catalyst, the reaction mechanism is different from the case of Cu(I), and the major products are 1,5-disubstituted triazoles. Another more important difference is that Ru(I) catalysts work on the disubstituted alkynes to give 1,4,5-trisubstituted triazoles (Scheme 1) [18–19]. When both substituents of an internal alkyne are aromatic groups, the triazoles thus formed include the hexatriene motif in the structure.

Although a number of photochromic diarylethenes containing triazole groups have been reported [20–31], all of them use the triazole ring as the linker of the second functional molecule with the diarylethene core. To the best of our knowledge, no diarylethenes or terarylenes possessing the triazole ring as one of the components of the hexatriene moiety has been reported to date.

**Scheme 2:**
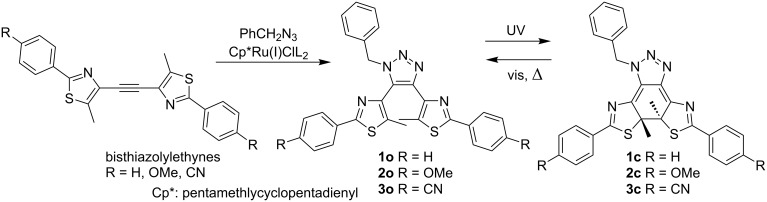
Synthesis and photochromism of bisthiazolyltriazoles.

Accordingly, we decided to employ a Ru(I) catalyst to synthesize 4,5-diaryl-1-substituted-1,2,3-triazoles **1o**–**3o** as possible photochromic compounds (Scheme 2).

## Results and Discussion

### Molecular design and synthesis

As the organic azide we used commercially available benzyl azide. Since 1,2-bis(5-methyl-2-phenylthiazol-4-yl)ethyne was used in our previous research [32–34], we employed bisthiazolylethynes as the foundation for the skeleton of the target compounds. In order to examine the substituent effects of the terminal phenyl groups on the photochromic properties, compounds with methoxy groups or cyano groups at the *para*-position of the phenyl groups were also synthesized. To avoid the generation of isomers with a substitution pattern by Huisgen cyclization, the same substituents were introduced to both phenyl groups.

The Ru(I)-catalyzed Huisgen cyclization reaction proceeded rather smoothly to give **1o**, **2o** and **3o** in moderate chemical yields of 36%, 53% and 20%, respectively. Since the reported chemical yield of the reaction of tolan, the simplest bisarylethyne, and 2-phenylethyl azide was 63% [19], it could be considered reasonable that the reactions of the sterically more congested ethynes and benzyl azide gave triazoles with less chemical yields. Details of the synthesis are described in Supporting Information File 1.

### Photochromic reactions

#### Photochemical cyclizations

**Figure 1:**
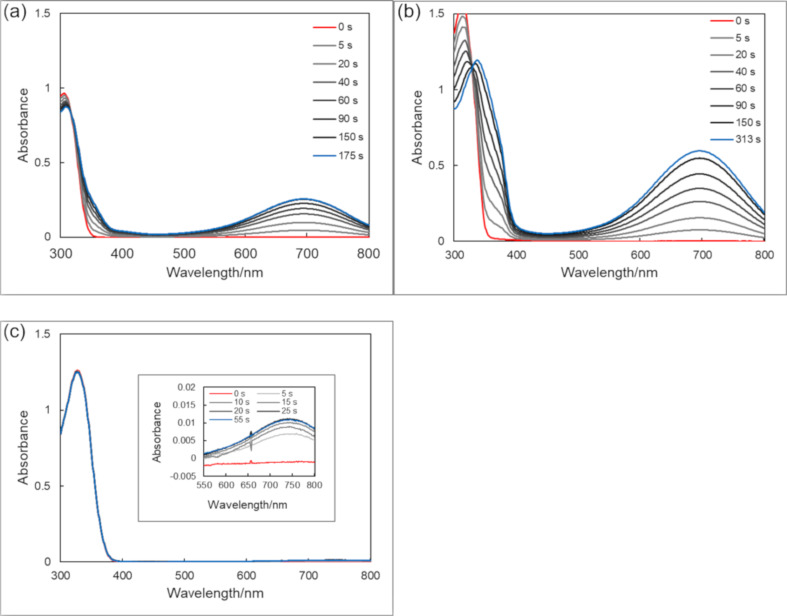
Absorption spectral change of triazoles **1o**–**3o** upon irradiation of 313 nm light in MeCN at 28 °C. Light intensity: 1.8 mW cm^−2^. (a) **1o**. 4.44 × 10^−5^ mol dm^−3^. (b) **2o**. 4.38 × 10^−5^ mol dm^−3^. (c) **3o**. 4.73 × 10^−5^ mol dm^−3^.

Triazoles were dissolved in ethanol (EtOH), acetonitrile (MeCN), ethyl acetate (AcOEt) and toluene, and each solution was irradiated with 313 nm light. The changes in the absorption spectra during UV light irradiation in MeCN are shown in Figure 1 (spectra of the compounds in the other solvents are shown in Figures S1–S9 in Supporting Information File 1). The absorption spectral properties are summarized in Table 1 together with their predicted absorption maxima obtained by TD DFT calculations in vacuum [35]. Although **1o** and **2o** showed substantial coloration, **3o** showed only a slight coloration at room temperature.

**Table 1:** Wavelengths of absorption maxima of the closed forms of photochromic triazoles.

Solvent and calculation method	EtOH	MeCN	AcOEt	toluene	TD DFT^a^

*e*_r _^b^ *E*_T_^N c^	24.55 0.654	35.94 0.460	6.02 0.228	2.38 0.099	in vacuum

	λ_max_/nm				

**1c** (R = H)	692	695	693	698	704
**2c** (R = OMe)	697	696	693	699	699
**3c** (R = CN)	737	739	737	739	759

^a^In vacuum [35]. ^b^Relative dielectric constant. ^c^Normalized *E*_T_(30) value.

The solvent effects on the absorption maximum wavelengths of the closed forms were not remarkable. The tendency observed was of the absorption maximum wavelength being a few nanometers longer in less polar toluene than for the other solvents.

**Scheme 3:**
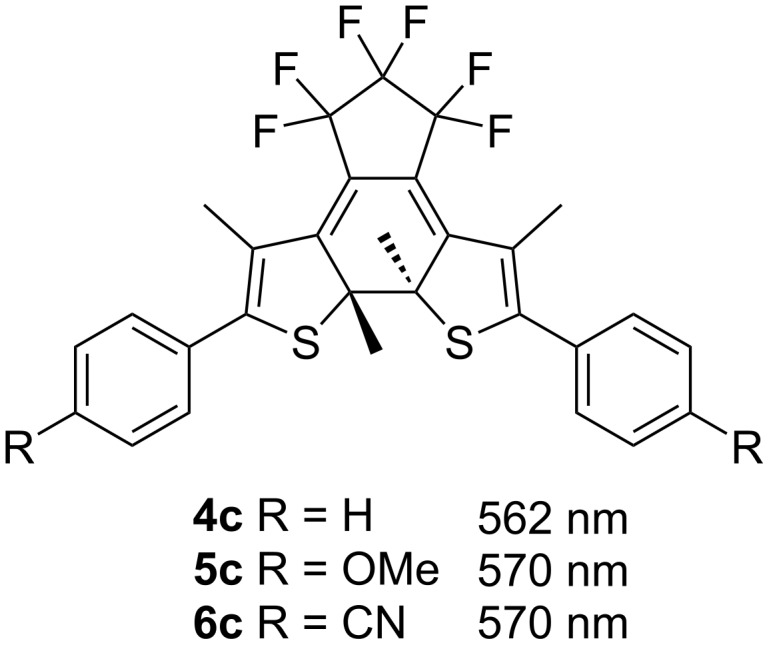
Wavelengths of absorption maxima of the closed forms of bisthienyletenes in hexane [36].

When the substituents on the terminal phenyl rings are electron-withdrawing cyano groups, the measured absorption maximum of the closed form was about 40 nm longer than the others in any solvent. The substituent effect observed here is different from the small substituent effect on the closed forms of the representative bisthienylhexafluorocyclopentenes **4c** (R = H, 562 nm), **5c** (R = OMe, 570 nm) and **6c** (R = CN, 570 nm) in hexane (Scheme 3) [36]. In triazoles, while the introduction of electron-donating methoxy groups had little effect on the absorption maximum wavelengths, the introduction of electron-withdrawing cyano groups induced a large bathochromic shift. This observation was well-reproduced by TD DFT calculations (Table 1 and chapter SI-4 in Supporting Information File 1).

**Scheme 4:**
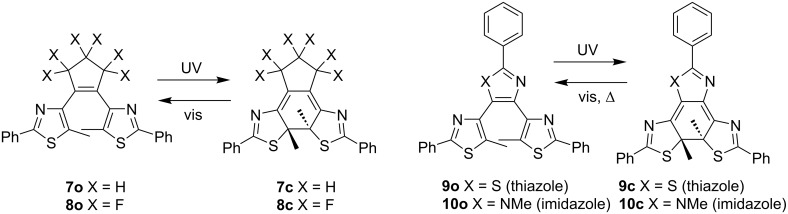
Photochromism of closely related compounds.

When the absorption maximum wavelength of **1c**, whose central ethene moiety is triazole, is compared with those of the closely related **7c** (cyclopentene) [37], **8c** (hexafluorocyclopentene) [38], **9c** (thiazole) [39], and **10c** (imidazole) [40] in non-polar solvents, **1c** has a much longer absorption maximum in toluene (Scheme 4, Table 2). It should also be noted that the absorption maximum wavelength is longer when the central ethene moiety is part of the aromatic ring than when it is an isolated ethene in their open-form structures.

**Table 2:** Absorption spectral data of triazoles and other related photochromic compounds.

	λ_max_/nm	
	In solution	TD DFT^a^

**1c**	698^b^	704
**7c**	500^c^ [37]	537
**8c**	525^d^ [38]	541
**9c**	587^d^ [39]	636
**10c**	654^d^ [40]	668

^a^In vacuum [35]. ^b^In toluene. ^c^In cyclohexane. ^d^In hexane.

#### Thermal back reactions

**Figure 2:**
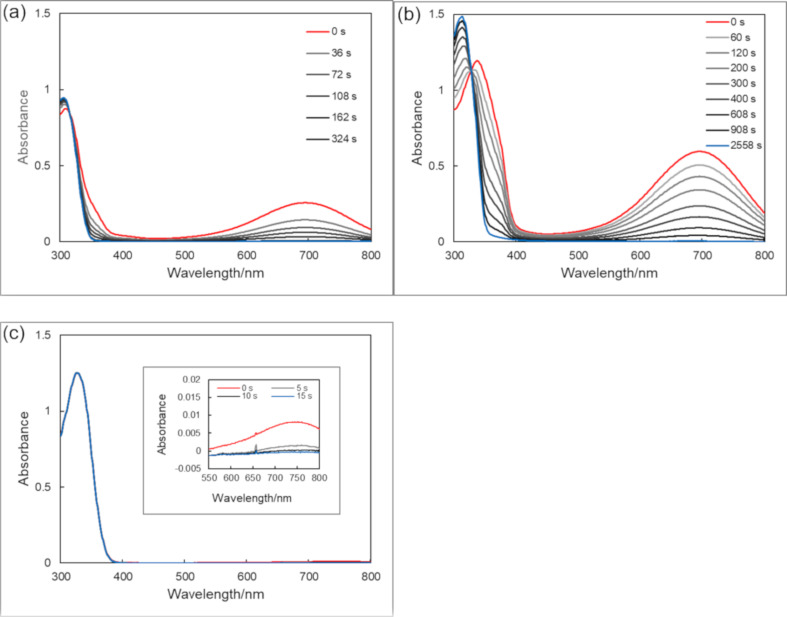
Absorption spectral change of triazoles **1c**–**3c** during the thermal back reaction after 313-nm light irradiation to **1o**–**3o** in MeCN at 28 °C. Concentration of compounds are the same as in Figure 1. (a) **1c**. (b) **2c**. (c) **3c**.

As expected, all closed forms of triazoles showed thermal back reactions since the photochemical cyclization results in the loss of the aromatic stabilization energy [41] of the three contiguous aromatic rings. The changes in the absorption spectra of thermal decoloration in MeCN at room temperature are shown in Figure 2.

In order to clarify the nature of the thermal back reaction of triazoles, **1o**, **2o** and **3o** were irradiated with 313 nm light in four different solvents, and the decrease in the absorbance of the absorption maximum wavelengths in the visible region was observed at three different temperatures. The first order reaction rate constants of the thermal back reactions at different temperatures were then determined. Arrhenius plots of ln *k* against 1/*T* gave pre-exponential factors (A) and Arrhenius activation energy *E_a_* of these thermally reversible photochromic triazoles (Figures S10–S13 in Supporting Information File 1). The kinetic data of the thermal back reactions of **1c**–**3c** in toluene are shown in Table 3 together with the literature data of related compounds **9c** [39] and **10c** [40] shown in Scheme 4.

**Table 3:** Kinetic data of thermal back reactions and aromatic stabilization energy of **1c**–**3c**, **9c** and **10c**.

	*A*/s^−1^	*E_a_*/kJ mol^−1^	*k* (293K)/s^−1^	*t*_1/2_ (293 K)^a^	ASE^b^/kJ mol^−1^

**9c**^c^	7.1 × 10^11^	112	6.7 × 10^−9d^	3.3 years	72.9
**10c**^c^	1.3 × 10^9^	85	8.7 × 10^−7d^	9.2 days	78.6
**1c**^e^	1.21 × 10^13^	87.8	2.80 × 10^−3^	248 s	102.0
**2c**^e^	2.79 × 10^12^	87.9	6.14 × 10^−4^	1130 s	102.0
**3c**^e^	1.26 × 10^13^	81.4	3.92 × 10^−2^	17.7 s	102.0

^a^*t*_1/2_ (293 K): Half-life at 293 K. ^b^ASE: Aromatic stabilization energy of the central aromatic rings when unsubstituted. Data taken from ref. [41]. ^c^In toluene. Data taken from ref. [39] for **9** and ref. [40] for **10**. ^d^Calculated from *t*_1/2_ at 293 K.^e^In toluene.

As shown in Table 3, the thermal back reaction is fast when the aromatic stabilization energy of the central aromatic ring is large. Although *E_a_* of **10** is smaller than that of **1** and **2**, the pre-exponential factors (*A*) of **1** and **2** are about 10^4^ times and 10^3^ times larger, respectively, than that of **10**.

When the thermal kinetic data of **1**, **2** and **3** are compared, the thermal back reaction rate of **3** is extremely faster than the others, although the central aromatic ring is common to these compounds.

**Scheme 5:**
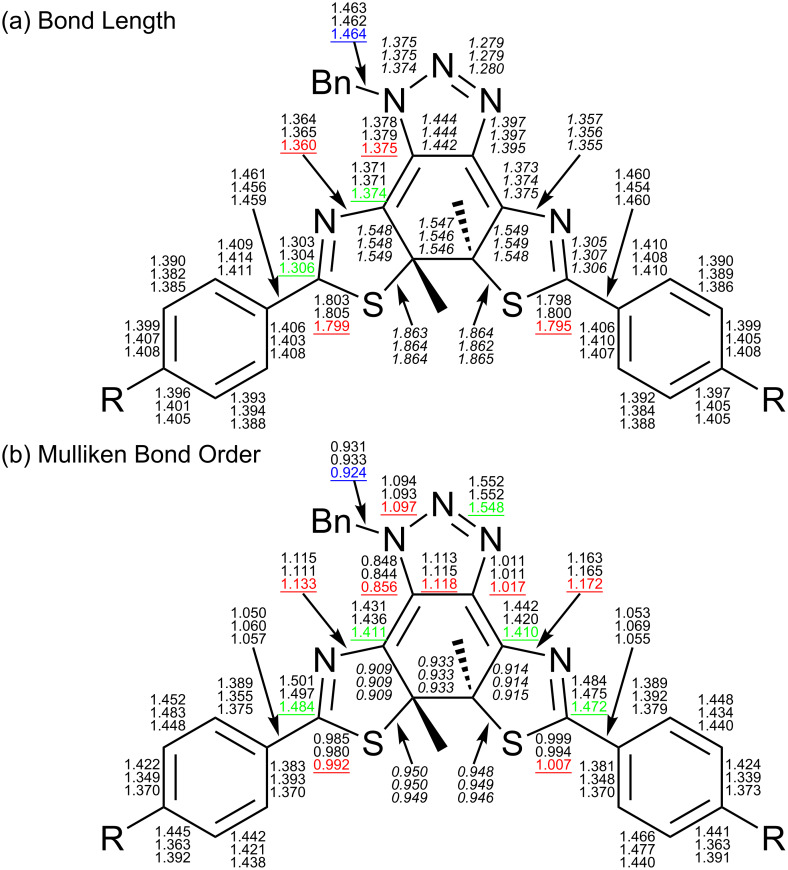
Bond length (a) (in Å) and Mulliken bond order (b) of **1c**–**3c** obtained by DFT calculations. Top number of each set: **1c**. Second number: **2c**. Bottom number: **3c**. Red numbers of **3c**: indicates stronger double bond character. Green numbers of **3c**: indicates stronger single bond character. Blue numbers of **3c**: indicates weaker bond character. Italic numbers around the central conjugation systems: no significant difference between the three compounds.

As shown in Table 4, neither electronic charge distribution on the carbon atoms (natural charge [42]) comprising the thermally breaking C–C bond, its bond length nor its bond order, which were obtained by DFT calculations of these compounds, did not give a clear explanation for the difference in the reaction rate. Possible evidence of the fast back reaction of **3c** may be found in the bond lengths and Mulliken bond orders between the atoms constructing the conjugation system around the central cyclohexadiene moiety (Scheme 5).

**Table 4:** Natural charge, bond length and bond order of the bond-breaking carbon atoms of triazoles **1c** – **3c** in the thermal back reactions obtained by DFT calculations.

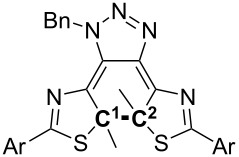				

	Natural charge^a^		Bond length/Å	Mulliken bond order
	C^1^	C^2^	C^1^–C^2^	C^1^–C^2^

**1c**	−0.198	−0.204	1.547	0.933
**2c**	−0.195	−0.204	1.546	0.933
**3c**	−0.198	−0.204	1.546	0.933

^a^Calculated by DFT calculations as the number of positive charges [42].

In Scheme 5a, the single bonds of **3c** written in red are shorter than in the other two compounds, implying that they have a stronger double bond character. Similarly, the double bonds of **3c** written in green are longer, showing the stronger single bond character. This suggests a stronger bond alteration tendency in **3c** than in **1c** and **2c**, and this distinctive character of **3c** can be seen more clearly in Scheme 5b.

In addition, since the bond order of the N–Bn bond in blue of **3c** is the smallest, it can be reasonably assumed that the lone pair on this nitrogen atom participates in the conjugation in the molecular core more strongly than in **1c** and **2c**. This distinctive character of **3c** originates from the electron-withdrawing cyano groups in the molecule.

Data on the terminal phenyl rings in Scheme 5 do not give any convergent tendency of the substituents due to the electron-donating character of the methoxy groups in **2c** and the electron-withdrawing character of the cyano groups in **3c**. However, it is safe to say the cyano groups in **3c** are pulling the electrons and this effect reaches the nitrogen atom bearing the benzyl group through the conjugation.

**Scheme 6:**
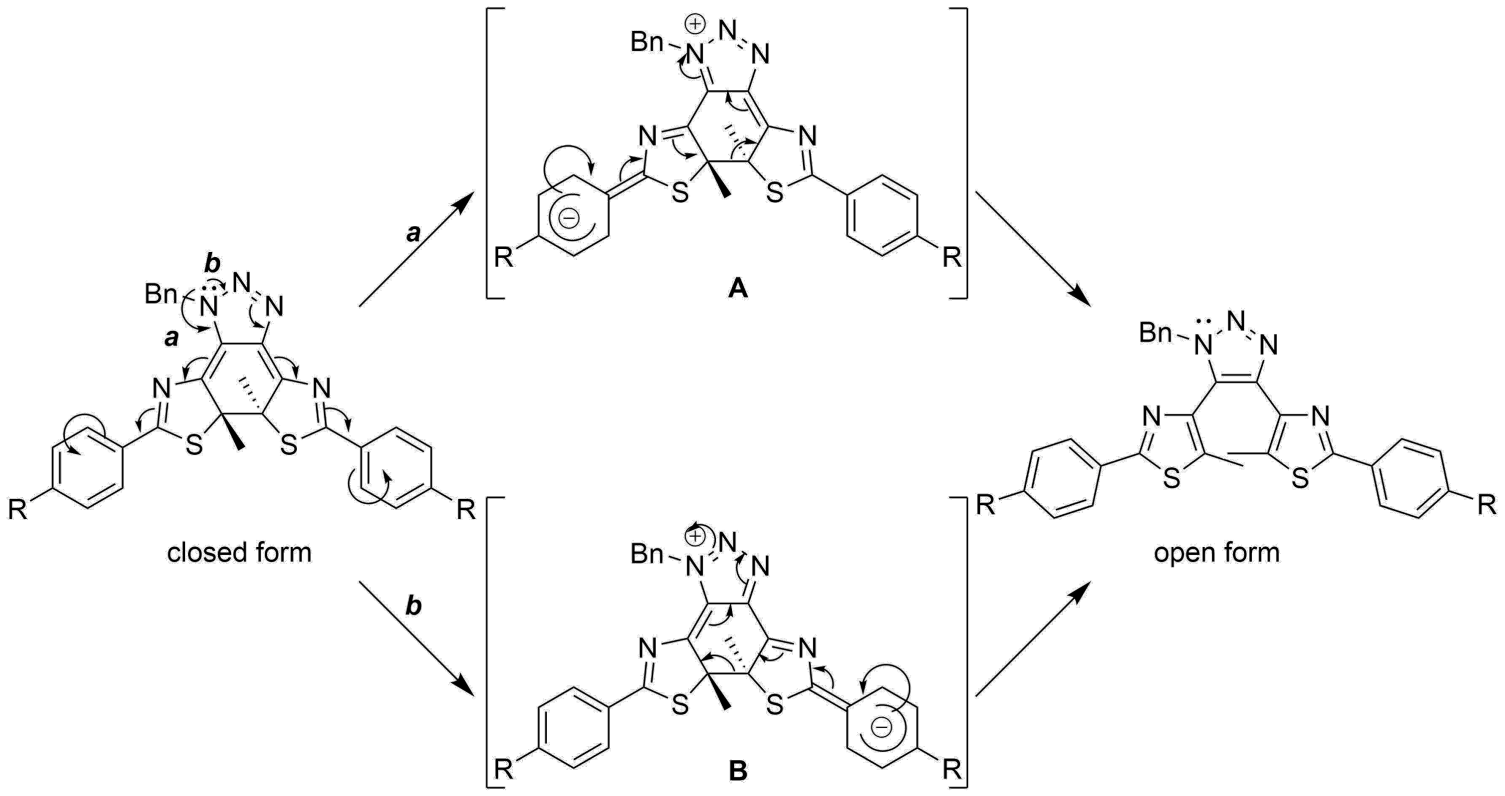
Possible reaction mechanism of thermal ring opening of the closed forms.

Then how does this effect accelerate the thermal back reaction in **3c**? We propose the conventional reaction mechanism shown in Scheme 6. When the delocalized lone pair on the nitrogen atom moves back from the resonance structures **A** and **B**, it may break the central C–C single bond to give the open form, as shown with the arrows. The resonance structures are stabilized more strongly when R is a cyano group so that the C–C bond scission occurs easily. Thus, it is possible to control the rate of the thermal back reaction by: (1) changing the aromatic ring at the ethene moiety, and (2) changing the substituents on the phenyl rings at the peripheral of the molecule.

Finally, we would like to discuss the solvent effects on the thermal back reactions of **1c**, **2c**, and **3c**. As can be seen in Figure S13, the solvents are classified into two categories: (1) polar solvents (EtOH and MeCN) and (2) less polar solvents (AcOEt and toluene). Although the gradient of the lines (*E*a) for the same compound in Figure S13a–c are quite similar, the intercepts (*A*) are clearly different for the two solvent groups of **1c** and **2c**. As for **3c**, although its categorization is not as clear as **1c** and **2c**, differences can still be observed. In general, the thermal back reactions are faster in the more polar solvents, and faster in MeCN than in EtOH among these two solvents. However, for **2c**, it is faster in EtOH than in MeCN. This may have originated from the hydrogen-bond formation between EtOH molecules and the methoxy groups in **2c**, which reduces the electrostatic repulsion between the methoxy groups and the negatively charged phenyl groups in the intermediate resonance structures **A** and **B** shown in Scheme 6.

In less polar solvents, the thermal back reactions of **1c** and **2c** are faster in toluene than in AcOEt. However, the reaction of **3c** is the opposite. This could be explained by the strong electron-withdrawing power of the cyano groups in **3c**, which enhanced the charge-separation character in the intermediate resonance structures **A** and **B**. These structures are stabilized more strongly in more polar AcOEt than in toluene, and the thermal back reaction rate in AcOEt was increased.

## Conclusion

We have synthesized three novel thermally reversible 4,4'-(1-benzyl-1*H*-1,2,3-triazole-4,5-diyl)bis(5-methyl-2-(4-substituted-phenyl)thiazole)s **1o**–**3o** by Ru(I)-catalysed Huisgen cyclization, which is a type of “click” reaction. They showed thermally reversible photochromism in various solvents. The absorption maximum wavelengths of **1c** (with unsubstituted peripheral phenyl groups) and **2c** (with methoxy groups on the phenyl groups) are close to 700 nm, while that of **3c** (with cyano groups on the phenyl groups) was about 740 nm. The thermal back reactions of these compounds proved that **3c** with the electron-withdrawing cyano groups is the fastest while **2c** with electron-donating methoxy groups was the slowest. DFT and TD DFT calculations supported these experimental results.

The Cu(I)-catalyzed Huisgen reaction has been used many times to connect two functional molecules. When the Ru(I)-catalyzed Huisgen reaction is employed to connect two functional molecules, the linker itself possesses the thermally reversible photochromic property. Thus, this work can open the door to the creation of promising new materials with highly integrated functions.

## Supporting Information

Experimental details including the synthesis of **1o**, **2o** and **3o**. Changes in the absorption spectra of **1o**, **2o** and **3o** in ethanol, ethyl acetate and toluene. Analysis processes of the thermal back reactions of **1o**, **2o** and **3o**. DFT and TD DFT calculation results of **1c**, **2c** and **3c** and ^1^H NMR, IR and mass spectra of new compounds.

File 1 Additional experimental data and spectra.
