# Supplementary material for: Click chemistry towards thermally reversible photochromic 4,5-bisthiazolyl-1,2,3-triazoles
Source: Beilstein J Org Chem. 2019 Sep 13;15:2161–9. doi: 10.3762/bjoc.15.213 (PMC6753672; doi:10.3762/bjoc.15.213)
Supplement: File 1 — Additional experimental data and spectra. [file Beilstein_J_Org_Chem-15-2161-s001.pdf]

BEILSTEIN JOURNAL OF ORGANIC CHEMISTRY

Supporting Information

for

Click chemistry towards thermally reversible photochromic 4,5-bisthiazolyl-1,2,3-triazoles

Chenxia Zhang, Kaori Morinaka, Mahmut Kose, Takashi Ubukata and Yasushi Yokoyama

Beilstein J. Org. Chem. **2019,** *15,* 2161–2169. doi:10.3762/bjoc.15.213

Additional experimental data and spectra

License and Terms: This is a supporting information file under the terms of the Creative Commons Attribution License (http://creativecommons.org/licenses/by/4.0). Please note that the reuse, redistribution and reproduction in particular requires that the authors and source are credited.
The license is subject to the *Beilstein Journal of Organic Chemistry* terms and conditions: (https://www.beilstein-journals.org/bjoc)

Table of contents

SI-1	Experimental details	p. S2
SI-2	Change in absorption spectra of **1o**, **2o**, and **3o** by photochromism	p. S10
SI-3	Analysis of thermal back reactions	p. S14
SI-4	DFT and TD DFT calculation results	p. S19
SI-5	^{1}H NMR spectra	p. S21
SI-6	IR spectra	p. S33
SI-7	Mass spectra	p. S45

SI-1. Experimental Details

General.

Chemical reactions were carried out under a dry nitrogen atmosphere. All solvents were used as received. All flash column chromatography purifications were carried out on 230–400 mesh silica gel using ethyl acetate and hexane or chloroform and hexane as eluent. Analytical thin-layer chromatography was performed on the pre-coated 0.25-mm thick silica gel TLC plates. ^{1}H NMR Spectra were recorded in deuteriochloroform ($CDCl_3$) with a 300 MHz NMR spectrometer. J values are expressed in Hz and quoted chemical shifts are in ppm. Splitting patterns are indicated as s, singlet; d, doublet; t, triplet; q, quartet, m, multiplet. Infrared spectra (IR) were recorded on a FTIR spectrometer. Low- and high-resolution mass spectra were measured by the electron ionization mass spectrometry using a Mass spectrometer. Ultraviolet and visible spectra were recorded on a UV–vis spectrophotometer equipped with a photodiode array detector and a temperature-controllable cell holder. Melting points were measured using a hot stage microscope, and those were uncorrected.

Photochemical reactions were all carried out in a 10 mm path length quartz cell. Photoirradiation with 313 nm light was carried out using a 500 W high-pressure mercury lamp, separated by filters (a 5 cm water filter, a UV-D35 glass filter, a 5 cm aqueous $NiSO_4 \cdot 6H_2O$ solution, a 1 cm aqueous K_2CrO_4 solution, and a 1 cm aqueous potassium diphthalate solution). High-performance liquid chromatography (HPLC) equipped with a UV–vis detector and a silica gel column (20 mm diameter × 250 mm) with chloroform/hexane as the eluent was used for the purification of synthesized compounds.

Synthetic schemes of 4,5-bisthiazolyl-1,2,3-triazoles.

Synthesis of **1o**, **2o**, and **3o** were carried out according to the following procedures.

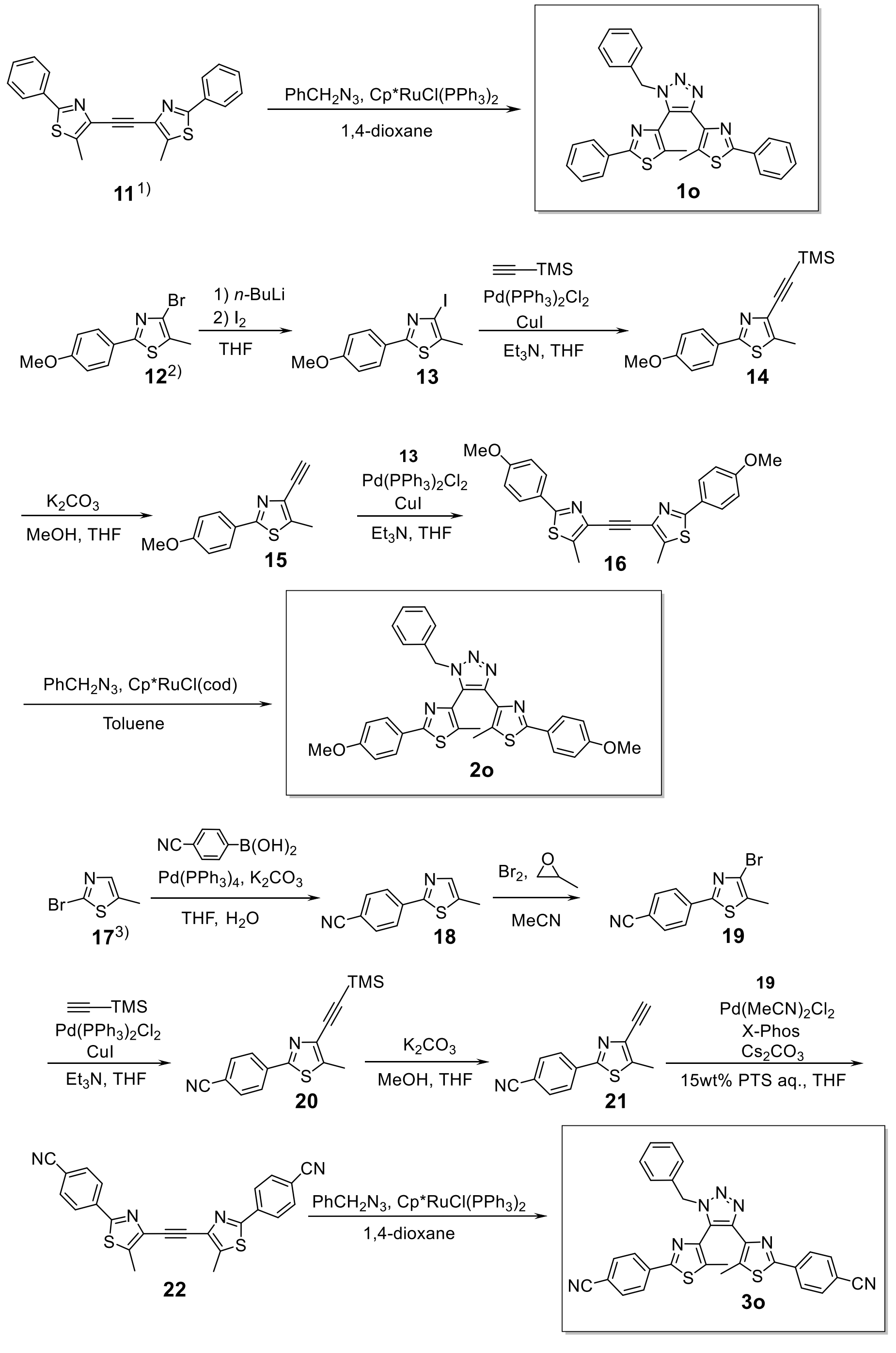

Cp*: pentamethylcyclopentadienyl

1) Morinaka, K.; Ubukata, T.; Yokoyama, Y. *Org. Lett.* **2009,** *11,* 3890-3893.

2) Li, R.; Nakashima, T.; Galangau, O.; Iijima, S.; Kanazawa, R.; Kawai, T. *Chem. Asian J.* **2015,** *10,* 1725-1730.

3) Commercially available.

Synthesis of 4,4'-(1-benzyl-1*H*-1,2,3-triazole-4,5-diyl)bis(5-methyl-2-phenylthiazole) (1o)

A mixture of 1,2-bis(5-methyl-2-phenylthiazol-4-yl)ethyne (**11**, 202.1 mg, 0.543 mmol, 1.00 equiv), benzyl azide (0.560 mL, 4.48 mmol, 8.28 equiv), Cp*RuCl(PPh_3)$_2$ (56.6 mg, 0.0711 mmol, 0.13 equiv) and 1,4-dioxane (4.00 mL) was refluxed for 43 h under a N_2 atmosphere with vigorous stirring. The solvent was removed in vacuo. The residue was purified by flash column chromatography on silica gel using ethyl acetate/hexane (15%) as the eluent, to give 98.6 mg (0.195 mmol) of 4,4'-(1-benzyl-1*H*-1,2,3-triazole-4,5-diyl)bis(5-methyl-2-phenylthiazole) **1o** as a pale yellow solid in 36% yield.

^{1}H NMR (300 MHz, $CDCl_3$, TMS) δ/ppm 1.83 (3H, s), 2.73 (3H, s), 5.70 (2H, s), 7.04-7.07 (2H, m), 7.20-7.23 (3H, m), 7.31-7.33 (3H, m), 7.46-7.48 (3H, m), 7.66-7.69 (2H, m), 7.94-7.97 (2H, m).

LRMS (EI, 70 eV) m/z (rel intensity), 505 (M^+, 75), 477 ($(M-N_2)^+$, 100), 386 ($(M-119)^+$, 63).

IR (neat) ν/cm^{-1} 1485, 1456, 1440, 1254, 972, 761, 689.

Found: m/z 505.14133. Calcd for $C_{29}H_{23}N_5S_2$: M, 505.13950.

Mp 61–64 °C.

Synthesis of 4-iodo-2-(4-methoxyphenyl)-5-methylthiazole (13)

To a solution of 4-bromo-2-(4-methoxyphenyl)-5-methylthiazole (**12**, 3.2 g, 11 mmol, 1.0 equiv) in anhydrous THF (140 mL) was added dropwise a hexane solution of *n*-butyllithium (1.63 mol dm^{-3}, 8.28 mL, 13 mmol, 1.1 equiv) at –78 °C under a N_2 atmosphere. The resulting solution was stirred at this temperature for 40 min, then a solution of I_2 (5.144 g, 20 mmol, 1.8 equiv) in anhydrous THF (40 mL) was added through a cannula, and the mixture was stirred for overnight with gradual warming up to room temperature. The reaction was quenched by adding 10% aq. $Na_2S_2O_3$, and the resultant mixture was extracted with ethyl acetate. The combined organic layer was dried over anhydrous Na_2SO_4, the drying agent filtered off, and evaporated. The residue was purified by flash column chromatography on silica gel using ethyl acetate/hexane (5% to 15%) as the eluent, to give 3.47 g (10 mmol) of 4-iodo-2-(4-methoxyphenyl)-5-methylthiazole **13** as a white solid in 93% yield.

^{1}H NMR (300 MHz, $CDCl_3$, TMS) δ/ppm 2.43 (3H, s), 3.85 (3H, s), 6.92 (2H, d(AA'BB'), J/Hz = 8.9), 7.80 (2H, d(AA'BB'), J/Hz = 9.0).

LRMS (EI, 70 eV) m/z (rel intensity), 331 (M^+, 100), 316 ($(M-Me)^+$, 10).

IR (neat) ν/cm^{-1} 3010, 1603, 1516, 1436, 1248, 1170, 1011, 972, 831, 799.

Mp 131–132 °C.

Synthesis of 2-(4-methoxyphenyl)-5-methyl-4-((trimethylsilyl)ethynyl)thiazole (14)

To a solution of 4-iodo-2-(4-methoxyphenyl)-5-methylthiazole (**13**, 1.5 g, 4.5 mmol, 1.0 equiv) in anhydrous THF (50 mL) and triethylamine (50 mL) was added dichlorobis(triphenylphosphine)palladium(II) (158.9 mg, 0.227 mmol, 0.05 equiv) and copper(I)

iodide (43 mg, 0.226 mmol, 0.05 equiv). To the resulting solution was added an excess of trimethylsilylethyne (1.5 mL, 10 mmol, 2.4 equiv) under a N_2 atmosphere, and the mixture was stirred for overnight at room temperature. The reaction was quenched by adding water and 3 mol dm^{-3} HCl aq, and the resultant mixture was extracted with ethyl acetate. The combined organic layer was dried over anhydrous Na_2SO_4, the drying agent filtered off, and evaporated. The residue was purified by flash column chromatography on silica gel using ethyl acetate/hexane (0% to 3%) as the eluent, to give 1.2480 g (4.14 mmol) of 2-(4-methoxyphenyl)-5-methyl-4-((trimethylsilyl)ethynyl)thiazole (**14**) as a white solid in 92% yield.

^{1}H NMR (300 MHz, $CDCl_3$, TMS) δ/ppm 0.28 (9H, s), 2.54 (3H, s), 3.84 (3H, s), 6.91 (2H, d(AA'BB'), J/Hz = 8.9), 7.84 (2H, d(AA'BB'), J/Hz = 9.0).

LRMS (EI, 70 eV) m/z (rel intensity), 301 (M^+, 100), 286 ($(M\text{-}Me)^+$, 75).

IR (neat) ν/cm^{-1} 2961, 2154, 1605, 1307, 1248, 1168, 1101, 1027, 893, 840, 826, 760, 656, 516.

Mp 92–93 °C.

Synthesis of 4-ethynyl-2-(4-methoxyphenyl)-5-methylthiazole (15)

A suspension of 2-(4-methoxyphenyl)-5-methyl-4-((trimethylsilyl)ethynyl)thiazole (**14**, 1.248 g, 4.14 mmol, 1.0 equiv) and K_2CO_3 (3.433 g, 25 mmol, 6 equiv) in methanol (45 mL) and THF (60 mL) was stirred at room temperature for 3 h. The resulting suspension was evaporated to half volume in vacuo. The resultant mixture was poured into water, and the resultant mixture was extracted with ethyl acetate. The combined organic layer was dried over anhydrous Na_2SO_4, filtered, and evaporated. The residue was purified by flash column chromatography on silica gel using ethyl acetate/hexane (10% to 20%) as the eluent, to give 949.0 mg (4.14 mmol) of 4-ethynyl-2-(4-methoxyphenyl)-5-methylthiazole (**15**) as a pale yellow solid in quantitative yield.

^{1}H NMR (300 MHz, $CDCl_3$, TMS) δ/ppm 2.55 (3H, s), 3.29 (1H, s), 3.85 (3H, s), 6.93 (2H, d(AA'BB'), J/Hz = 8.9), 7.84 (2H, d(AA'BB'), J/Hz = 9.0).

LRMS (EI, 70 eV) m/z (rel intensity), 229 (M^+, 100), 214 ($(M\text{-}Me)^+$, 13).

IR (neat) ν/cm^{-1} 3258, 2919, 1605, 1522, 1254, 1239, 1171, 1021, 826, 680, 509.

Mp 136–137 °C.

Synthesis of 1,2-bis(2-(4-methoxyphenyl)-5-methylthiazol-4-yl)ethyne (16)

A solution of 4-ethynyl-2-(4-methoxyphenyl)-5-methylthiazole (**15**, 0.80 g, 3.5 mmol, 1.0 equiv) and 4-iodo-2-(4-methoxyphenyl)-5-methylthiazole (**13**, 1.15 g, 3.5 mmol, 1.0 equiv) in anhydrous THF (10 mL) and triethylamine (10 mL) was treated with dichlorobis(triphenylphosphine)palladium(II) (122.8 mg, 0.175 mmol, 0.05 equiv) and copper(I) iodide (0.02 g, 0.11 mmol, 0.03 equiv). The resulting solution was stirred at room temperature for overnight under a N_2 atmosphere. The reaction was quenched by adding water and 3 mol dm^{-3} HCl aq., and the resultant mixture was extracted with ethyl acetate. The combined

organic layer was dried over anhydrous Na_2SO_4, the drying agent filtered off, and evaporated. The residue was purified by flash column chromatography on silica gel using chloroform/hexane (10% to 30%) as the eluent, to give 28.0 mg (0.065 mmol) of 1,2-bis(2-(4-methoxyphenyl)-5-methylthiazol-4-yl)ethyne (**16**) as a pale yellow solid in 2% yield.

^{1}H NMR (300 MHz, $CDCl_3$, TMS) δ/ppm 2.64 (6H, s), 3.86 (6H, s), 6.95 (4H, d(AA'BB'), J/Hz = 8.9), 8.87 (4H, d(AA'BB'), J/Hz = 8.9).

LRMS (EI, 70 eV) m/z (rel intensity), 432 (M^+, 100), 216 (M^{2+}, 18).

IR (neat) ν/cm^{-1} 2924, 2851, 1604, 1519, 1436, 1307, 1255, 1172, 1033, 829, 721, 511.

Found: m/z 432.09632 Calcd for $C_{24}H_{20}N_2O_2S_2$: M, 432.09663.

Mp 224–229 °C.

Synthesis of 4,4'-(1-benzyl-1*H*-1,2,3-triazole-4,5-diyl)bis(2-(4-methoxyphenyl)-5-methylthiazole) (2o)

A mixture of 1,2-bis(2-(4-methoxyphenyl)-5-methylthiazol-4-yl)ethyne (**16**, 25 mg, 0.058 mmol, 1.0 equiv), benzyl azide (0.0435 mL, 0.35 mmol, 6.0 equiv), Cp*RuCl(cod) (6.6 mg, 0.017 mmol, 0.3 equiv), and toluene (2 mL) was stirred at room temperature for 2 days under a N_2 atmosphere. The solvent was removed in vacuo. The residue was purified by flash column chromatography on silica gel using ethyl acetate/hexane (0% to 30 %) as the eluent, to give 17.0 mg (0.031 mmol) of 4,4'-(1-benzyl-1*H*-1,2,3-triazole-4,5-diyl)bis(2-(4-methoxyphenyl)-5-methylthiazole) (**2o**) as a pale yellow solid in 53% yield.

^{1}H NMR (300 MHz, $CDCl_3$, TMS) δ/ppm 1.80 (3H, s), 2.67 (3H, s), 3.81 (3H, s), 3.88 (3H, s)

5.69 (2H, s), 6.84 (2H, d(AA'BB'), J/Hz = 8.9), 6.98 (2H, d(AA'BB'), J/Hz = 9.0), 7.04-7.08 (2H, m), 7.20-7.22 (3H, m) , 7.63 (2H, d(AA'BB'), J/Hz = 8.9), 7.84 (2H, d(AA'BB'), J/Hz = 8.9).

LRMS (EI, 70 eV) m/z (rel intensity), 565 (M^+, 26), 537 ($(M-N_2)^+$, 100), 446($(M-119)^+$, 63).

IR (neat) ν/cm^{-1} 2937, 1607, 1522, 1457, 1304, 1251, 1172, 1030, 832.

Found: m/z 565.17141 Calcd for $C_{31}H_{16}N_5O_2S_2$: M, 565.16063.

Mp 66–70 °C.

Synthesis of 2-(4-cyanophenyl)-5-methylthiazole (18)

A suspension of 2-bromo-5-methylthiazole (**17**, 2.01 g, 11.3 mmol, 1.00 equiv), *p*-cyanophenylboronic acid (1.8259 g, 12.4 mmol, 1.10 equiv), $Pd(PPh_3)_4$ (774.2 mg, 0.676 mmol, 0.06 equiv), and K_2CO_3 (6.3399 g, 45.9 mmol, 4.07 equiv) in THF (40 mL) and water (20 mL) was refluxed for 15 h under a N_2 atmosphere with vigorous stirring. The reaction was quenched by 3 mol dm^{-3} HCl aq., and the resultant mixture was extracted with ethyl acetate. The combined organic layer was dried over anhydrous Na_2SO_4, the drying agent filtered off, and evaporated. The residue was purified by flash column chromatography on silica gel using ethyl acetate/hexane (3% to 8 %) as the eluent, to give 1.6136 g (8.06 mmol) of 2-(4-cyanophenyl)-5-

methylthiazole (**18**) as a white solid in 71% yield.

^{1}H NMR (300 MHz, $CDCl_3$, TMS) δ/ppm 2.55 (3H, d, J/Hz = 1.1), 7.58 (1H, q, J/Hz = 1.1), 7.71 (2H, d(AA'BB'), J/Hz = 8.7), 7.99 (2H, d(AA'BB'), J/Hz = 8.7).

LRMS (EI, 70 eV) m/z (rel intensity), 200 (M^+, 100).

IR (neat) ν/cm^{-1} 2924, 1604, 1499, 1432, 1173, 1105, 972, 859, 820, 628, 524.

Mp 118–127 °C.

Synthesis of 4-bromo-2-(4-cyanophenyl)-5-methylthiazole (19)

To a solution of 2-(4-cyanophenyl)-5-methylthiazole (**18**, 1.5757 g, 7.87 mmol, 1.00 equiv) and propylene oxide (0.740 mL, 10.6 mmol, 1.34 equiv) in acetonitrile (100 mL) was added Br_2 (0.725 mL, 14.1 mmol, 1.80 equiv) dropwise at 0 °C. After the mixture was stirred for 5.5 h with gradual warming up to room temperature, the reaction was quenched by adding 10% aq. Na_2SO_3. Then the resultant mixture was extracted with ethyl acetate. The combined organic layer was washed with sat. aq. NaCl, and dried over anhydrous Na_2SO_4, the drying agent filtered off, and evaporated. The residue was purified by flash column chromatography on silica gel using ethyl acetate/hexane (5% to 6%) as the eluent, to give 1.6653 g (5.97 mmol) of 4-bromo-2-(4-cyanophenyl)-5-methylthiazole (**19**) as a white solid in 67% yield.

^{1}H NMR (300 MHz, $CDCl_3$, TMS) δ/ppm 2.48 (3H, s), 7.72 (2H, d(AA'BB'), J/Hz = 8.7), 7.98 (2H, d(AA'BB'), J/Hz = 8.7).

LRMS (EI, 70 eV) m/z (rel intensity), 280 ($(M+2)^+$, 100) 278 (M^+, 99), 199 ($(M-Br)^+$, 3), 146 (84), 71 (34).

IR (neat) ν/cm^{-1} 3059, 2225, 1605, 1500, 1215, 972, 844, 539.

Mp 174–175 °C.

Synthesis of 2-(4-cyanophenyl)-5-methyl-4-((trimethylsilyl)ethynyl)thiazole (20)

To a solution of 4-bromo-2-(4-cyanophenyl)-5-methylthiazole (**19**, 216.6 mg, 0.776 mmol, 1.0 equiv) and trimethylsilylethyne (0.700 mL, 4.95 mmol, 6.38 equiv) in anhydrous THF (4 mL) and triethylamine (4 mL) was added dichlorobis(triphenylphosphine)palladium(II) (8.6 mg, 0.0123 mmol, 0.016 equiv) and copper(I) iodide (2.3 mg, 0.0121 mmol, 0.016 equiv). The mixture was refluxed for 9 h under a N_2 atmosphere with vigorous stirring. The reaction was quenched by adding water and 3 mol dm^{-3} HCl aq, and the resultant mixture was extracted with ethyl acetate. The combined organic layer was washed with sat. aq. NaCl, and dried over anhydrous Na_2SO_4, the drying agent filtered off, and evaporated. The residue was purified by flash column chromatography on silica gel using ethyl acetate/hexane (2% to 20%) as the eluent, to give 102.6 mg (0.346 mmol) of 2-(4-cyanophenyl)-5-methyl-4-((trimethylsilyl)ethynyl)thiazole (**20**) as a pale yellow solid in 45% yield.

^{1}H NMR (300 MHz, $CDCl_3$, TMS) δ/ppm 0.29 (9H, s), 2.59 (3H, s), 7.70 (2H, d(AA'BB'), J/Hz = 8.6), 8.01 (2H, d(AA'BB'), J/Hz = 8.7).

LRMS (EI, 70 eV) m/z (rel intensity), 296 (M^+, 59), 281 ($(M\text{-}Me)^+$, 100).
IR (neat) ν/cm^{-1} 2958, 2223, 1770, 1605, 1498, 1247, 1103, 974, 844.
Mp 175–176 °C.

Synthesis of 4-ethynyl-2-(4-cyanophenyl)-5-methylthiazole (21)

A mixture of 2-(4-cyanophenyl)-5-methyl-4-((trimethylsilyl)ethynyl)thiazole (**20**, 90.9 mg, 0.307 mmol, 1.00 equiv) and K_2CO_3 (215.8 mg, 1.56 mmol, 5.09 equiv) in methanol (4 mL) and THF (4 mL) was stirred for overnight at room temperature. The resulting mixture was evaporated to half volume in vacuo. The resultant mixture was poured into water, and the resultant mixture was extracted with ethyl acetate. The combined organic layer was dried over anhydrous Na_2SO_4, filtered, and evaporated. The residue was purified by flash column chromatography on silica gel using ethyl acetate/hexane (5% to 25%) as the eluent, to give 52.5 mg (0.234 mmol) of 4-ethynyl-2-(4-cyanophenyl)-5-methylthiazole (**21**) as a light brown solid in 76% yield.
^1H NMR (300 MHz, $CDCl_3$, TMS) δ/ppm 2.61 (3H, s), 3.34 (1H, s), 7.71 (2H, d(AA'BB'), J/Hz = 8.9), 8.01 (2H, d(AA'BB'), J/Hz = 8.7).
LRMS (EI, 70 eV) m/z (rel intensity), 224 (M^+, 100).
IR (neat) ν/cm^{-1} 3300, 2994, 2359, 1769, 1241, 841.
Mp 168–170 °C.

Synthesis of 1,2-bis(2-(4-cyanophenyl)-5-methylthiazol-4-yl)ethyne (22)

A mixture of 2-(4-cyanophenyl)-4-ethynyl-5-methylthiazole (**21**, 180.0 mg, 3.5 mmol, 1.00 equiv), 4-bromo-2-(4-cyanophenyl)-5-methylthiazole (**19**, 239.6 mg, 0.858 mmol, 1.07 equiv), $Pd(MeCN)_2Cl_2$ (13.9 mg, 0.0536 mmol, 0.07 equiv), X-Phos (25.8 mg, 0.0541 mmol, 0.07 equiv), and Cs_2CO_3 (686.6 mg, 2.11 mmol, 2.63 equiv), in 15 wt% aq. polyoxyethanyl-α-tocopheryl sebacate (0.8 mL), water (3.2 mL), and THF (4.0 mL) was stirred at room temperature for overnight and heated for 1 h under a N_2 atmosphere. The resultant mixture was extracted with chloroform. The combined organic layer was washed with sat. aq. NaCl, and dried over anhydrous Na_2SO_4, the drying agent filtered off, and evaporated. The residue was purified by flash column chromatography on silica gel using chloroform/hexane (40% to 100%) as the eluent, to give 203.0 mg (0.480 mmol) of 1,2-bis(2-(4-cyanophenyl)-5-methylthiazol-4-yl)ethyne (**22**) as a pale yellow solid in 60% yield.
^1H NMR (300 MHz, $CDCl_3$, TMS) δ/ppm 2.70 (6H, s), 7.73 (4H, d(AA'BB'), J/Hz = 8.7), 8.04 (4H, d(AA'BB'), J/Hz = 8.7).
LRMS (EI, 70 eV) m/z (rel intensity), 422 (M^+, 100).
IR (neat) ν/cm^{-1} 2919, 2359, 1769, 1604, 1246, 972, 841, 542.
Found: m/z 422.06818 Calcd for $C_{24}H_{14}N_{14}S_2$: M, 422.06599.
Mp 246–249 °C.

Synthesis of 4,4'-(1-benzyl-1*H*-1,2,3-triazole-4,5-diyl)bis(2-(4-cyanophenyl)-5-methylthiazole) (3o)

A mixture of 1,2-bis(2-(4-cyanophenyl)-5-methylthiazol-4-yl)ethyne (**22**, 43.8 mg, 0.104 mmol, 1.00 equiv), benzyl azide (0.050 mL, 0.400 mmol, 3.86 equiv), Cp*RuCl(PPh_3)$_2$ (2.6 mg, 0.00327 mmol, 0.03 equiv), 1,4-dioxane 3.00 mL was stirred at 70 °C for 4.5 h and at 100 °C for 11.5 h under a N_2 atmosphere. The solvent was removed in vacuo. The residue was purified by flash column chromatography on silica gel using ethyl acetate/hexane (15% to 30%) as eluent, to give a mixture of **3o** and **22**. Further separation of the mixture of **3o** and **22** was carried out with semi-preparative HPLC (silica gel column, 60% chloroform/hexane as the eluent) to afford 11.7 mg (0.0211 mmol) of 4,4'-(1-benzyl-1*H*-1,2,3-triazole-4,5-diyl)bis(2-(4-cyanophenyl)-5-methylthiazole) (**3o**) as a white solid in 20% yield.

^{1}H NMR (300 MHz, $CDCl_3$, TMS) δ/ppm 1.86 (3H, s), 2.87 (3H, s), 5.65 (2H, s), 7.01-7.04 (2H, m), 7.22-7.24 (3H, m), 7.59 (2H, d(AA'BB'), J/Hz = 8.9), 7.70 (2H, d(AA'BB'), J/Hz = 8.7), 7.76 (2H, d(AA'BB'), J/Hz = 8.7), 8.03 (2H, d(AA'BB'), J/Hz = 8.7).

LRMS (EI, 70 eV) m/z (rel intensity), 555 (M^+, 32), 527 ($(M-N_2)^+$, 83), 436 ($(M-119)^+$, 82), 91 (100).

IR (neat) ν/cm^{-1} 2923, 2363, 2226, 1604, 1485, 1437,1022, 975, 838.

Found: m/z 555.11755 Calcd for $C_{31}H_{21}N_7S_2$: M, 555.12999.

Mp 89–95 °C.

SI-2. Change in absorption spectra of 1o, 2o, and 3o by photochromism

Photoirradiation conditions

Concentration:

1o: 4.44×10^{-5} mol dm^{-3}

2o: 4.38×10^{-5} mol dm^{-3}

3o: 4.73×10^{-5} mol dm^{-3}

Temperature: 28 ^{o}C

Irradiation light wavelength: 313 nm

Light intensity: 1.8 mW cm^{-2}

Optical path length: 1 cm

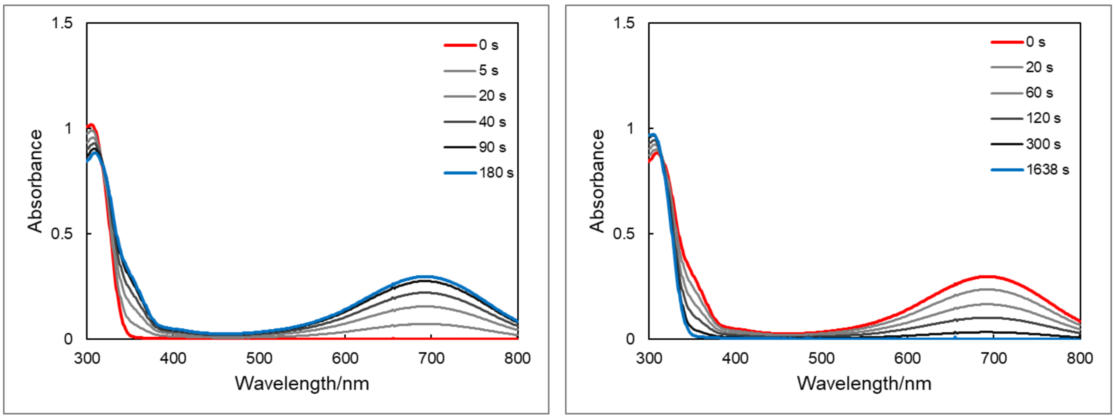

Fig. S1: Absorption spectral change of **1o** in ethanol (Left: photoirradiation. Right: thermal back reaction)

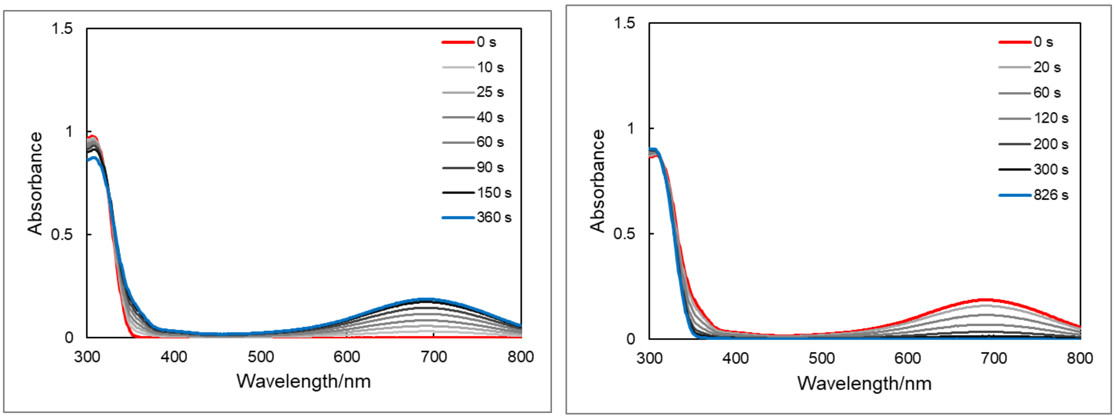

Fig. S2: Absorption spectral change of **1o** in ethyl acetate (Left: photoirradiation. Right: thermal back reaction)

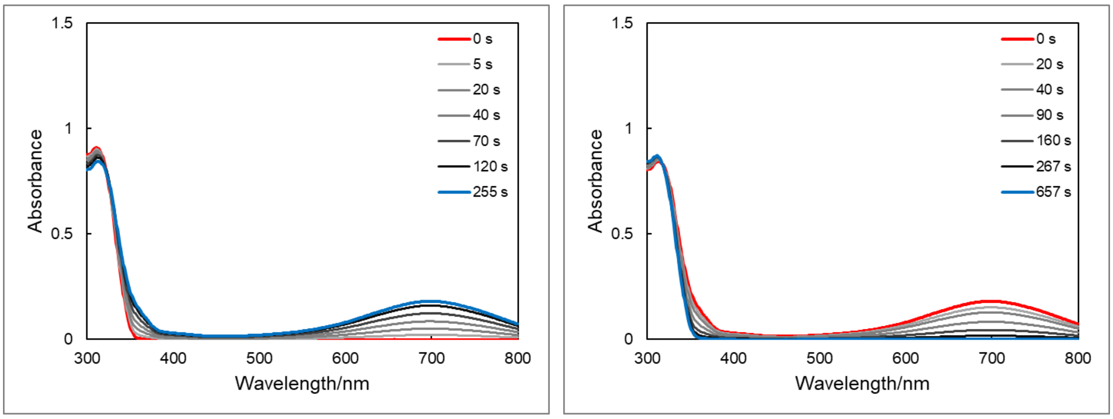

Fig. S3: Absorption spectral change of **1o** in toluene (Left: photoirradiation. Right: thermal back reaction)

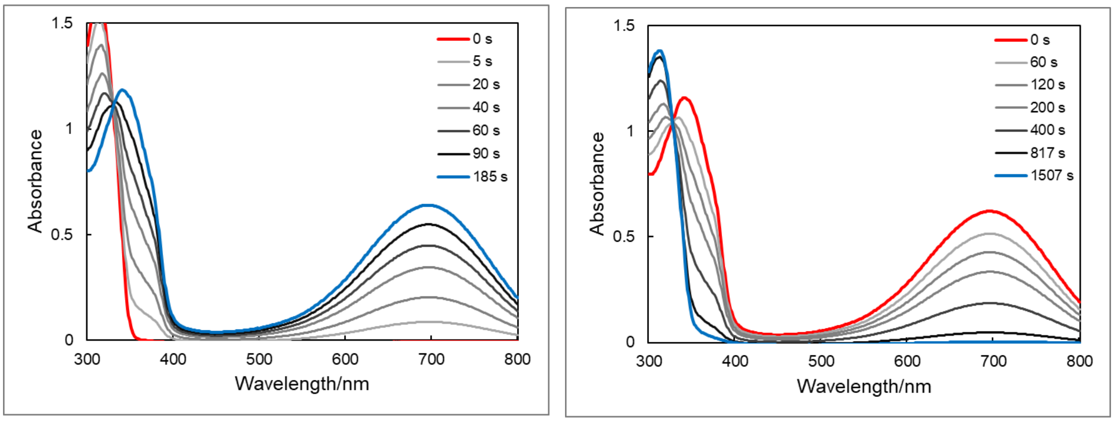

Fig. S4: Absorption spectral change of **2o** in ethanol (Left: photoirradiation. Right: thermal back reaction)

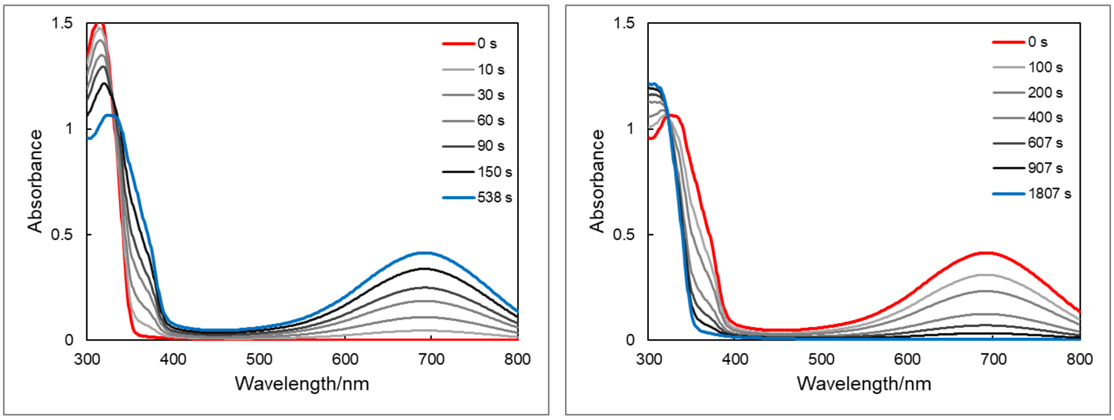

Fig. S5: Absorption spectral change of **2o** in ethyl acetate (Left: photoirradiation. Right: thermal back reaction)

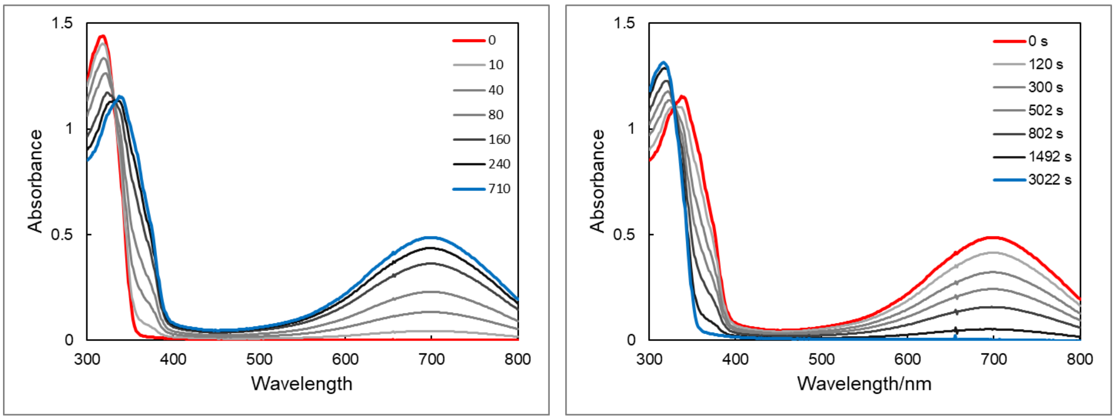

Fig. S6: Absorption spectral change of **2o** in toluene (Left: photoirradiation. Right: thermal back reaction)

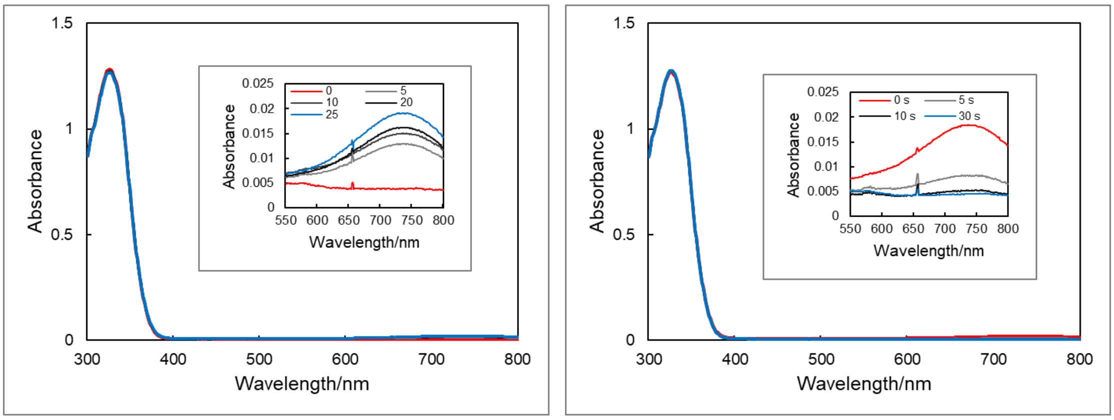

Fig. S7: Absorption spectral change of **3o** in ethanol (Left: photoirradiation. Right: thermal back reaction)

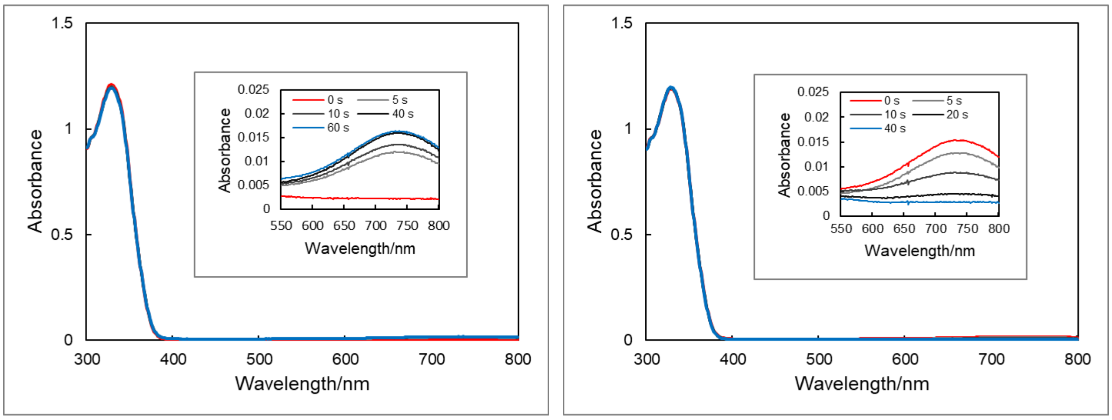

Fig. S8: Absorption spectral change of **3o** in ethyl acetate (Left: photoirradiation. Right: thermal back reaction)

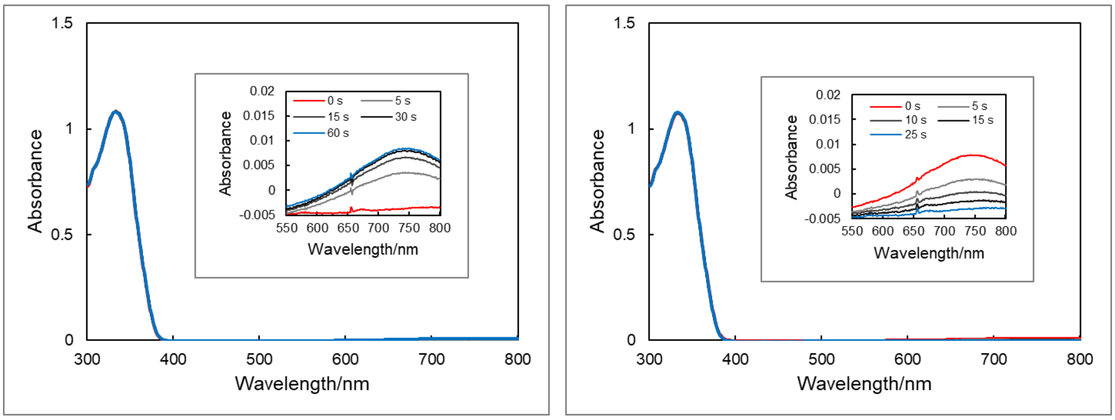

Fig. S9: Absorption spectral change of **3o** in toluene (Left: photoirradiation. Right: thermal back reaction)

SI-3. Analysis of thermal back reactions

Thermal back reaction conditions

Concentration:

1o: 4.44×10^{-5} mol dm^{-3}

2o: 1.46×10^{-5} mol dm^{-3}

3o: 9.46×10^{-5} mol dm^{-3}

Temperature:

1o: 20 ^{o}C, 25 ^{o}C, 30 ^{o}C

2o: 20 ^{o}C, 25 ^{o}C, 30 ^{o}C

3o: 0 ^{o}C, 5 ^{o}C, 10 ^{o}C

Irradiation light wavelength: 313 nm

Light intensity: 1.2 mW cm^{-2}

Optical path length: 1 cm

Analysis of thermal back reactions

Thermal back reactions of triazoles were carried out in ethanol, acetonitrile, ethyl acetate and toluene at three different temperatures. The decay curve of absorbance A_t at the absorption maximum wavelength of the compound in each solvent was traced, ln (A_t/A_0) was plotted against reaction time t, and the rate constant k of the compound at the temperature T was calculated.

Then, ln k was plotted against T^{-1} to obtain A (pre-exponential factor) and E_a (Arrhenius activation energy) from the intercept of y-axis and gradient of the first order equation, respectively.

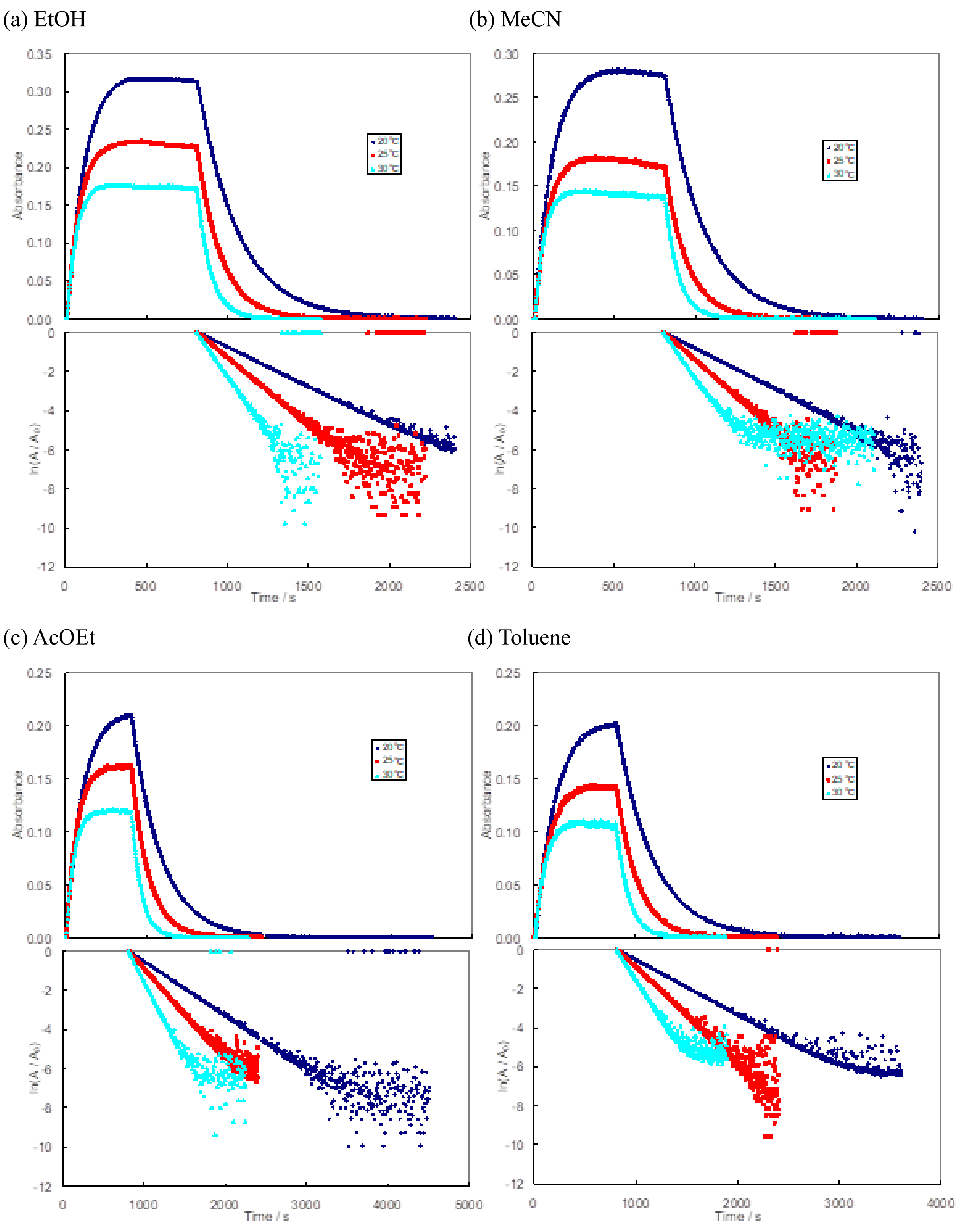

Fig. S10. Thermal back reaction of triazole **1c** at various temperatures in (a) EtOH, (b) MeCN, (c) AcOEt, (d) toluene

Top: Absorption spectral change, Bottom: Decay lines of absorption maximum: $A_0 = A_t$ at 810 s

Irradiation time: 10–810 s, Detection wavelength: λ_{max} in each solvent.

(a) EtOH

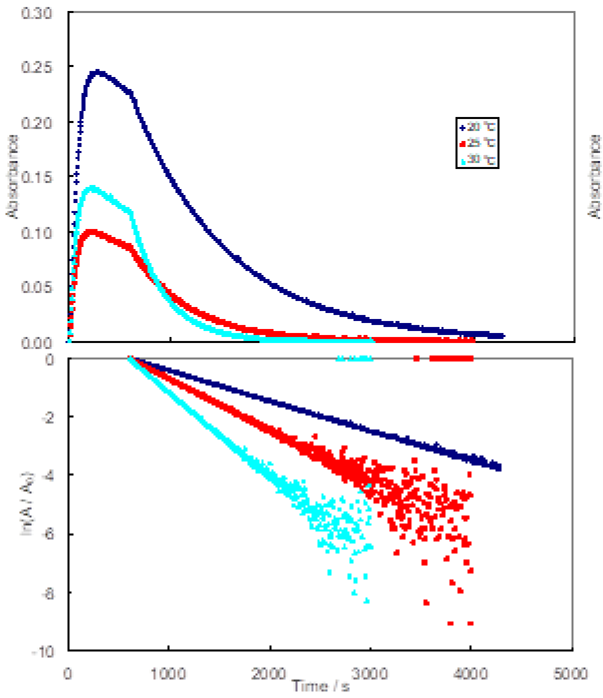

(b) MeCN

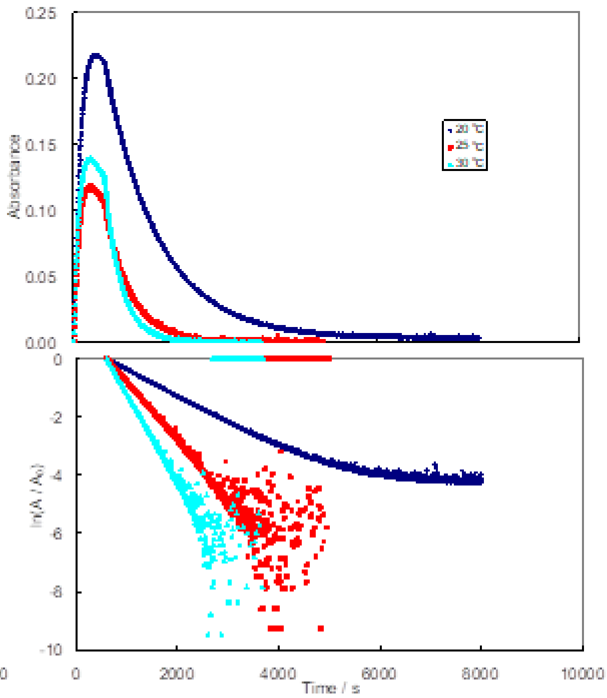

(c) AcOEt

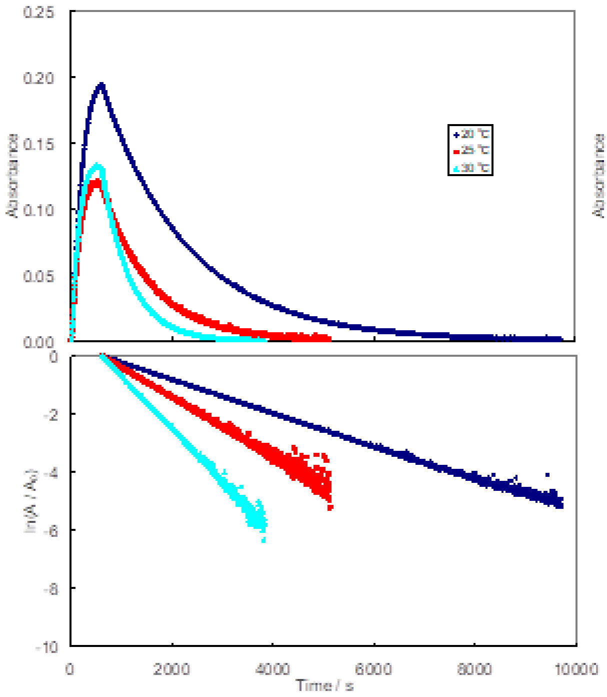

(d) Toluene

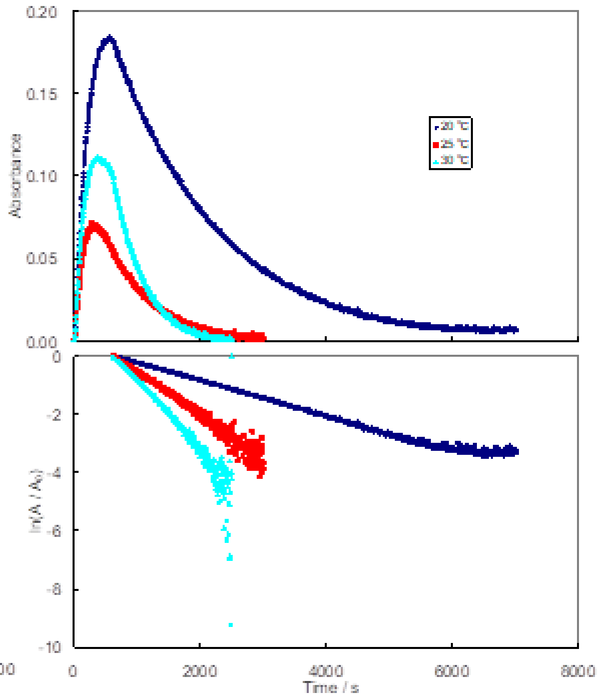

Fig. S11. Thermal back reaction of triazole **2c** at various temperatures in (a) EtOH, (b) MeCN, (c) AcOEt, (d) toluene

Top: Absorption spectral change, Bottom: Decay lines of absorption maximum: $A_0 = A_t$ at 610 s

Irradiation time: 10–610 s, detection wavelength: λ_{max} in each solvent

(a) EtOH

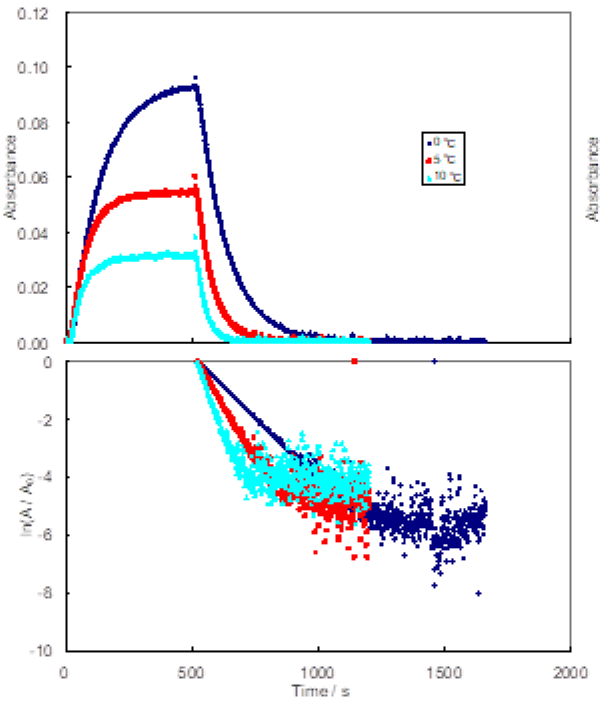

(b) MeCN

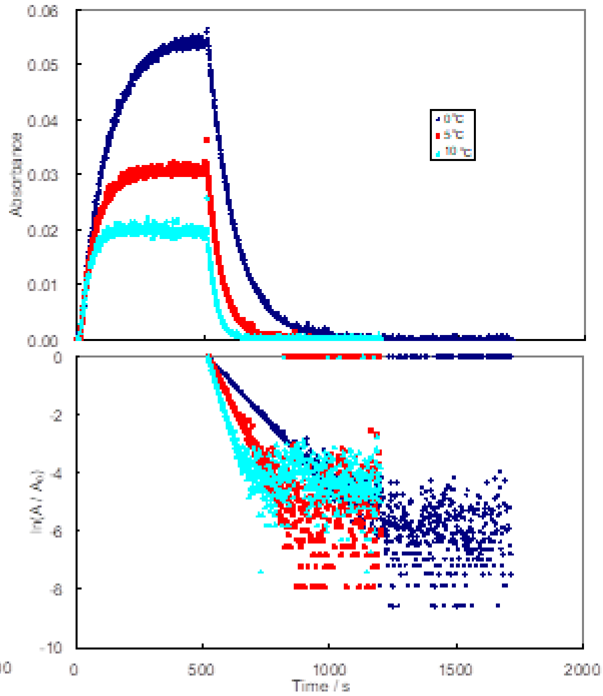

(c) AcOEt

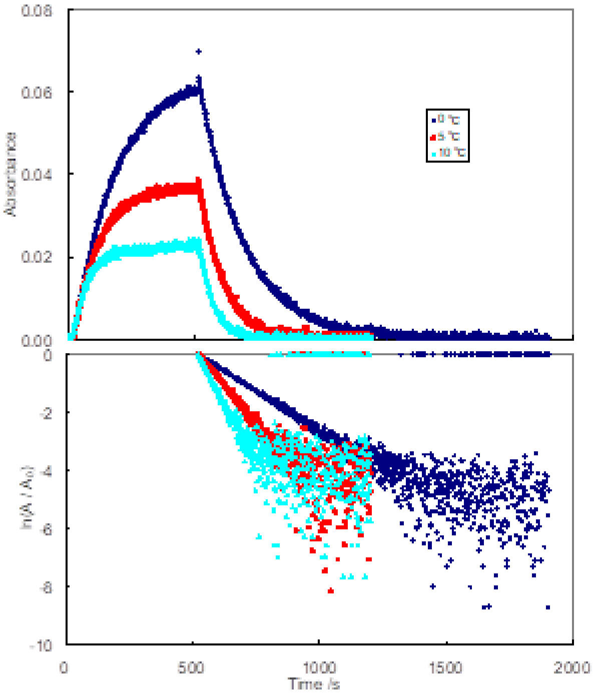

(d) Toluene

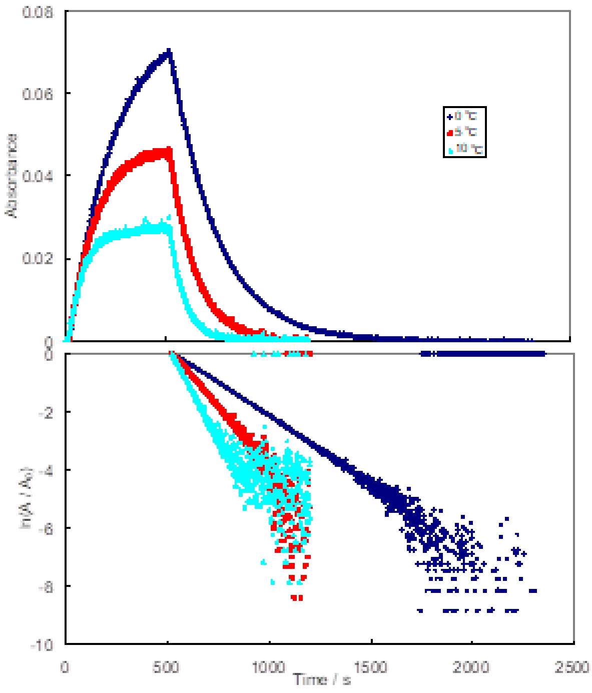

Fig. S12. Thermal back reaction of triazole **3c** at various temperatures in (a) EtOH, (b) MeCN, (c) AcOEt, (d) toluene

Top: Absorption spectral change, Bottom: Decay lines of absorption maximum: $A_0 = A_t$ at 521 s

Irradiation time: 10–510 s, Detection wavelength: λ_{max} in each solvent

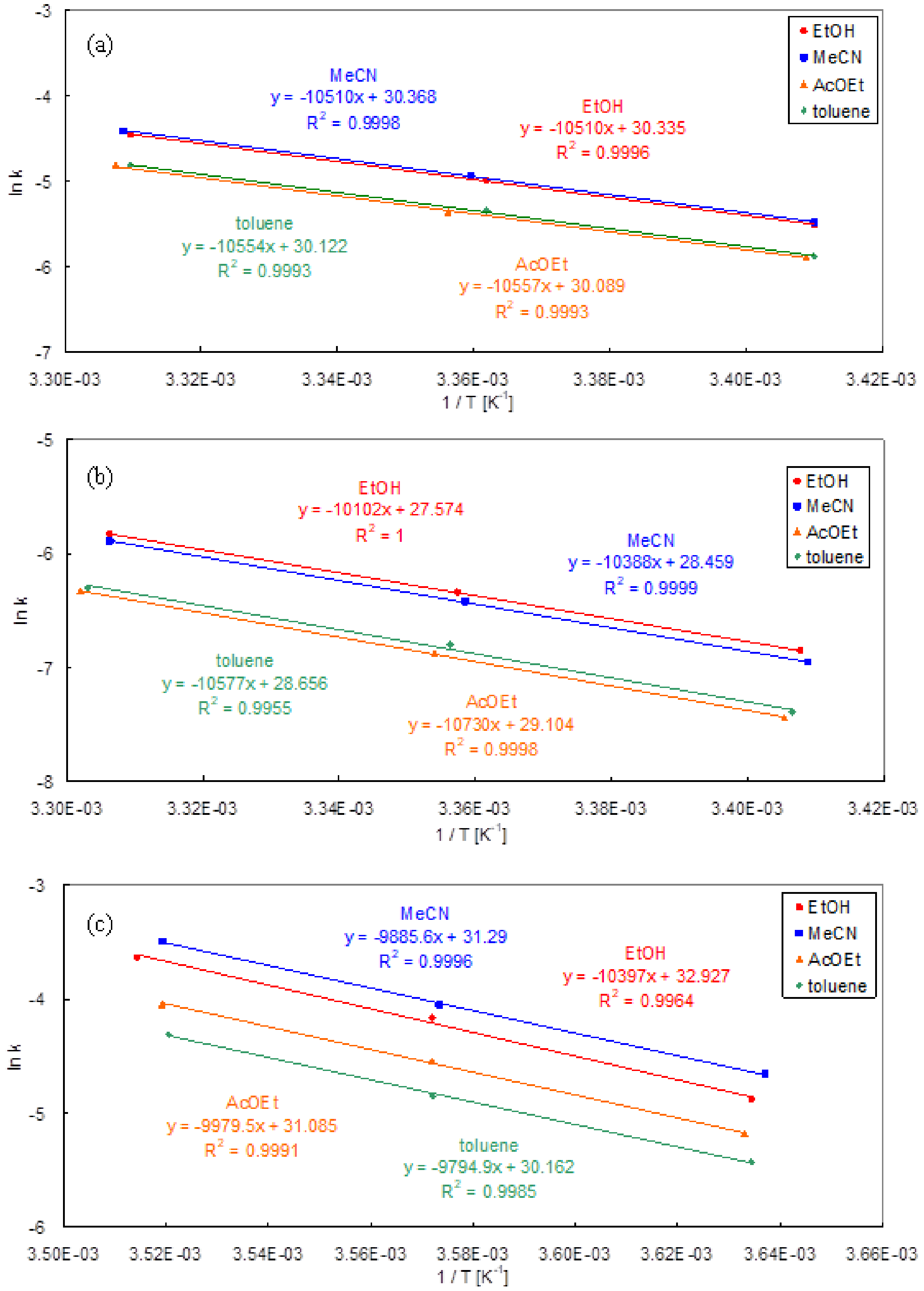

Fig. S13. Arrhenius plots. (a) triazole **1c**, (b) triazole **2c**, (c) triazole **3c**.

SI-4. DFT and TD DFT calculation results

Calculation conditions

DFT and TD DFT calculations of **1c–3c** were carried out with Spartan'18 software (Wavefunction) at the B3LYP/6-31G* level.

1c

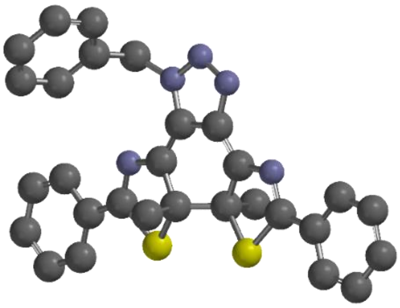

Allowed transitions

wavelength/nm	strength	MO Component	%
346.66	0.0174	HOMO-3 -> LUMO	79%
356.45	0.0233	HOMO-2 -> LUMO	75%
364.51	0.0497	HOMO-1 -> LUMO	67%
382.35	0.0314	HOMO -> LUMO+2	95%
430.24	0.0577	HOMO -> LUMO+1	88%
704.25	0.4309	HOMO -> LUMO	102%

2c

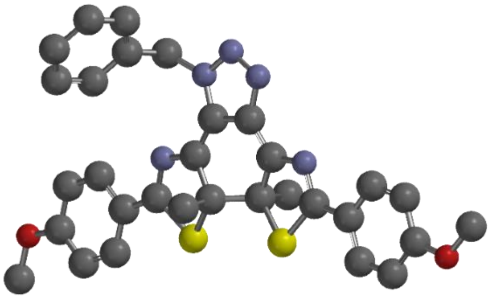

Allowed transitions

wavelength/nm	strength	MO Component	%
356.88	0.0875	HOMO-3 -> LUMO	65%
		HOMO-1 -> LUMO	21%
359.50	0.0453	HOMO -> LUMO+3	29%
		HOMO -> LUMO+6	26%
		HOMO-1 -> LUMO	22%
		HOMO-3 -> LUMO	14%
366.15	0.4453	HOMO-2 -> LUMO	86%
385.83	0.0117	HOMO -> LUMO+2	89%

428.46	0.0596	HOMO -> LUMO+1	84%
698.93	0.5445	HOMO -> LUMO	102%

3c

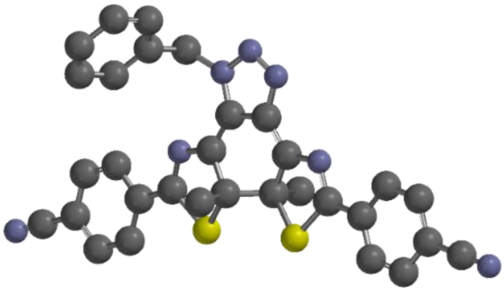

Allowed transitions

wavelength/nm	strength	MO Component	%
379.04	0.0508	HOMO-3 -> LUMO	89%
387.04	0.0001	HOMO-2 -> LUMO	98%
395.70	0.0106	HOMO-1 -> LUMO	95%
406.77	0.0433	HOMO -> LUMO+2	95%
478.47	0.0696	HOMO -> LUMO+1	92%
759.29	0.5479	HOMO -> LUMO	103%

SI-5. ^{1}H NMR spectra

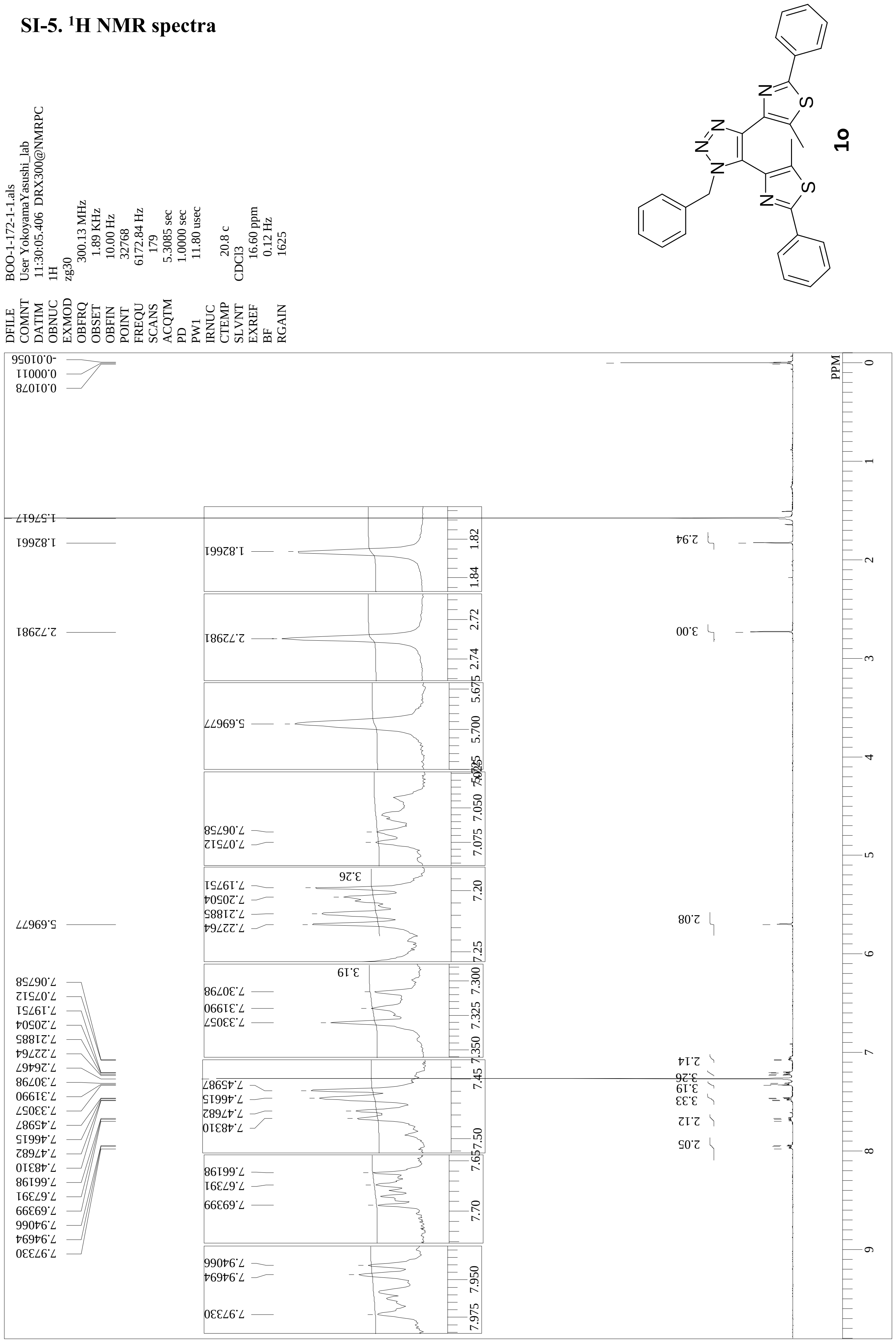

^{1}H NMR spectrum of compound **1o** (300 MHz, $CDCl_3$, TMS)

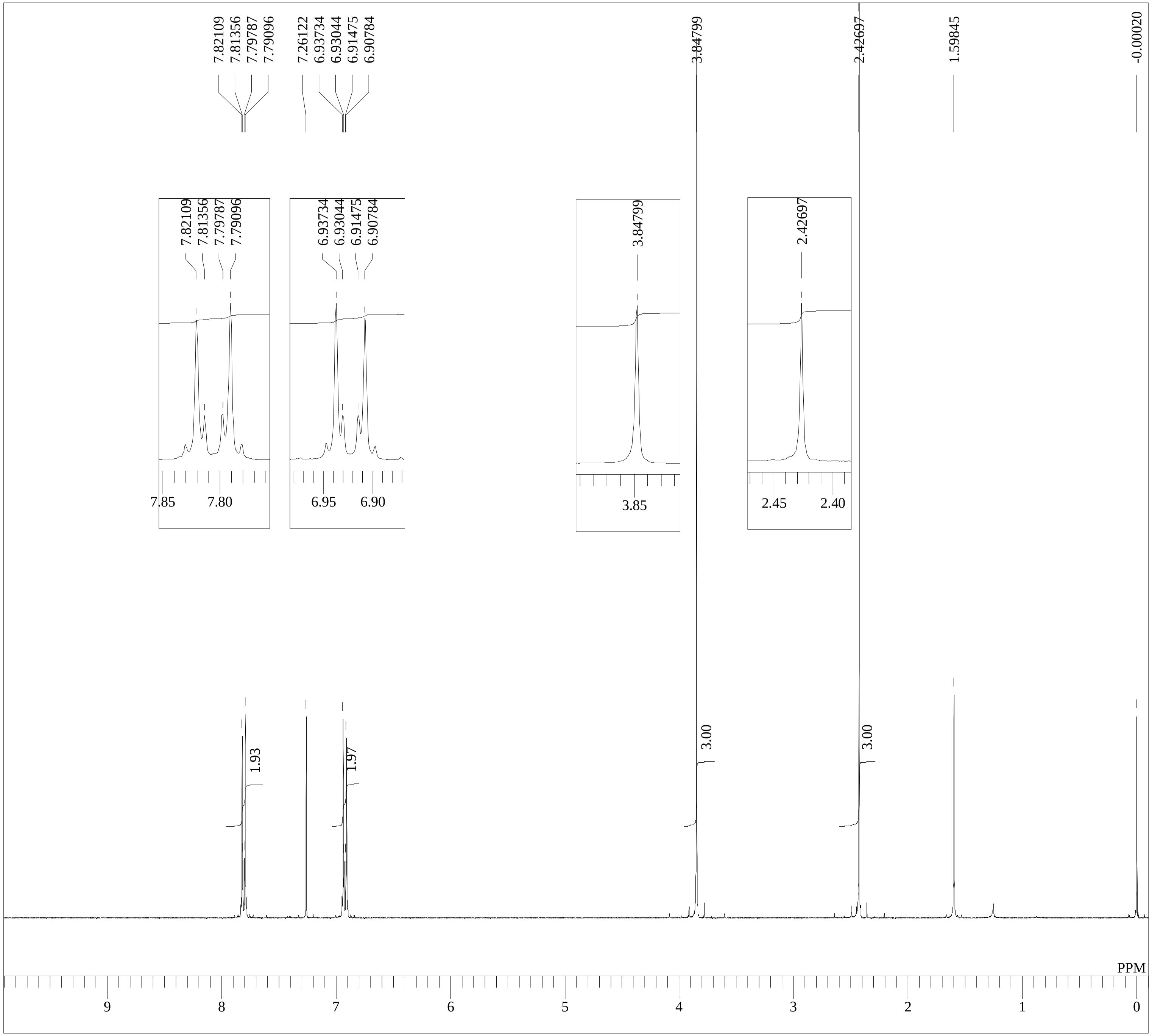

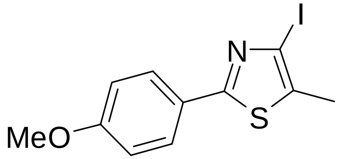

13

^{1}H NMR spectrum of compound **13** (300 MHz, $CDCl_3$, TMS)

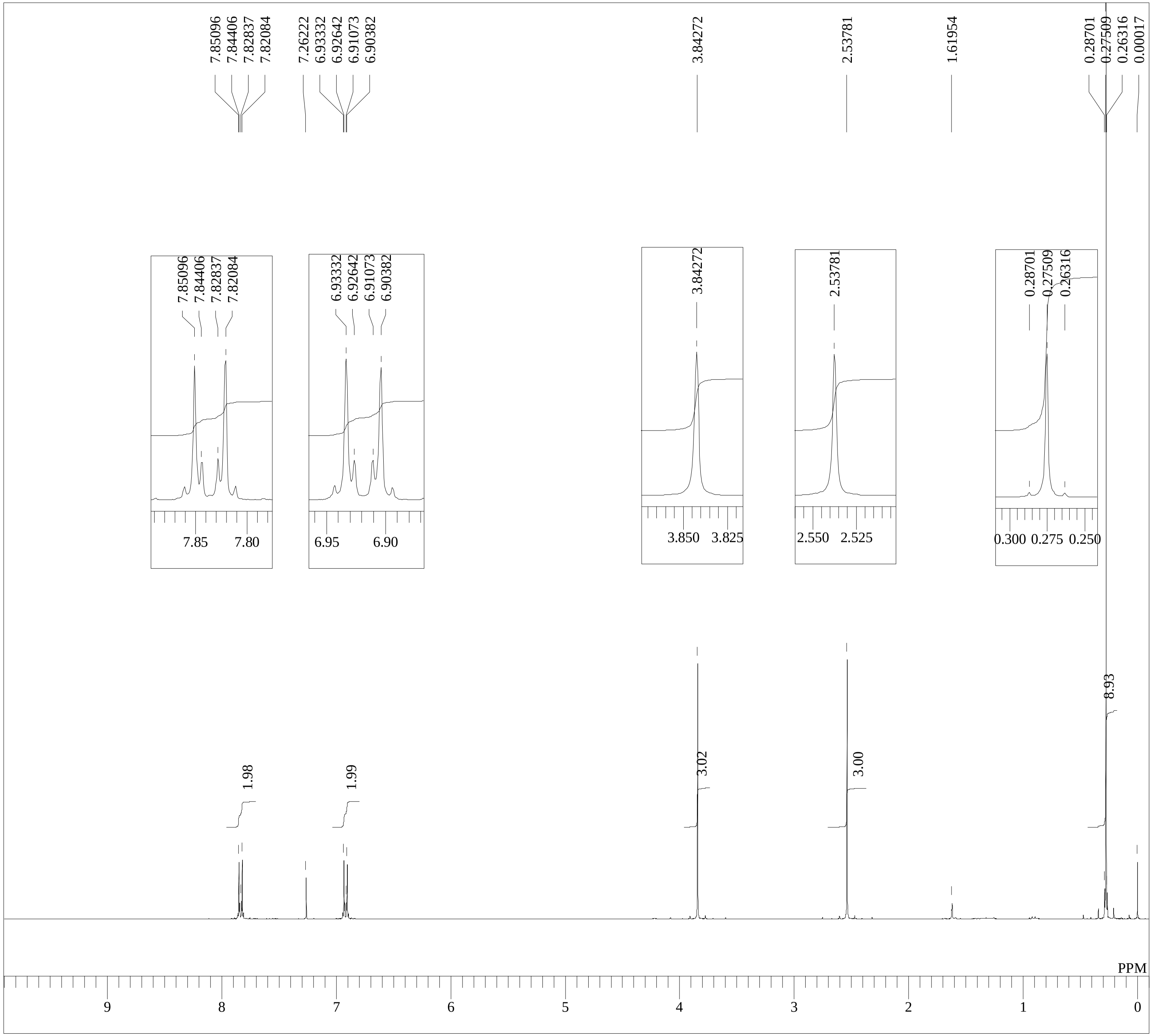

DFILE	kose83-1.als
COMNT	User YokoyamaYasushi_lab
DATIM	17:18:45.578 DRX300@NMRPC
OBNUC	1H
EXMOD	zg30
OBFRQ	300.13 MHz
OBSET	1.89 KHz
OBFIN	10.00 Hz
POINT	32768
FREQU	6172.84 Hz
SCANS	8
ACQTM	5.3085 sec
PD	1.0000 sec
PW1	14.00 usec
IRNUC	
CTEMP	27.5 c
SLVNT	CDCl3
EXREF	16.59 ppm
BF	0.12 Hz
RGAIN	406

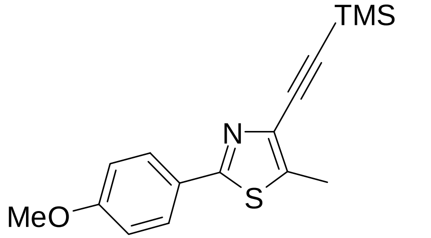

14

^{1}H NMR spectrum of compound **14** (300 MHz, $CDCl_3$, TMS)

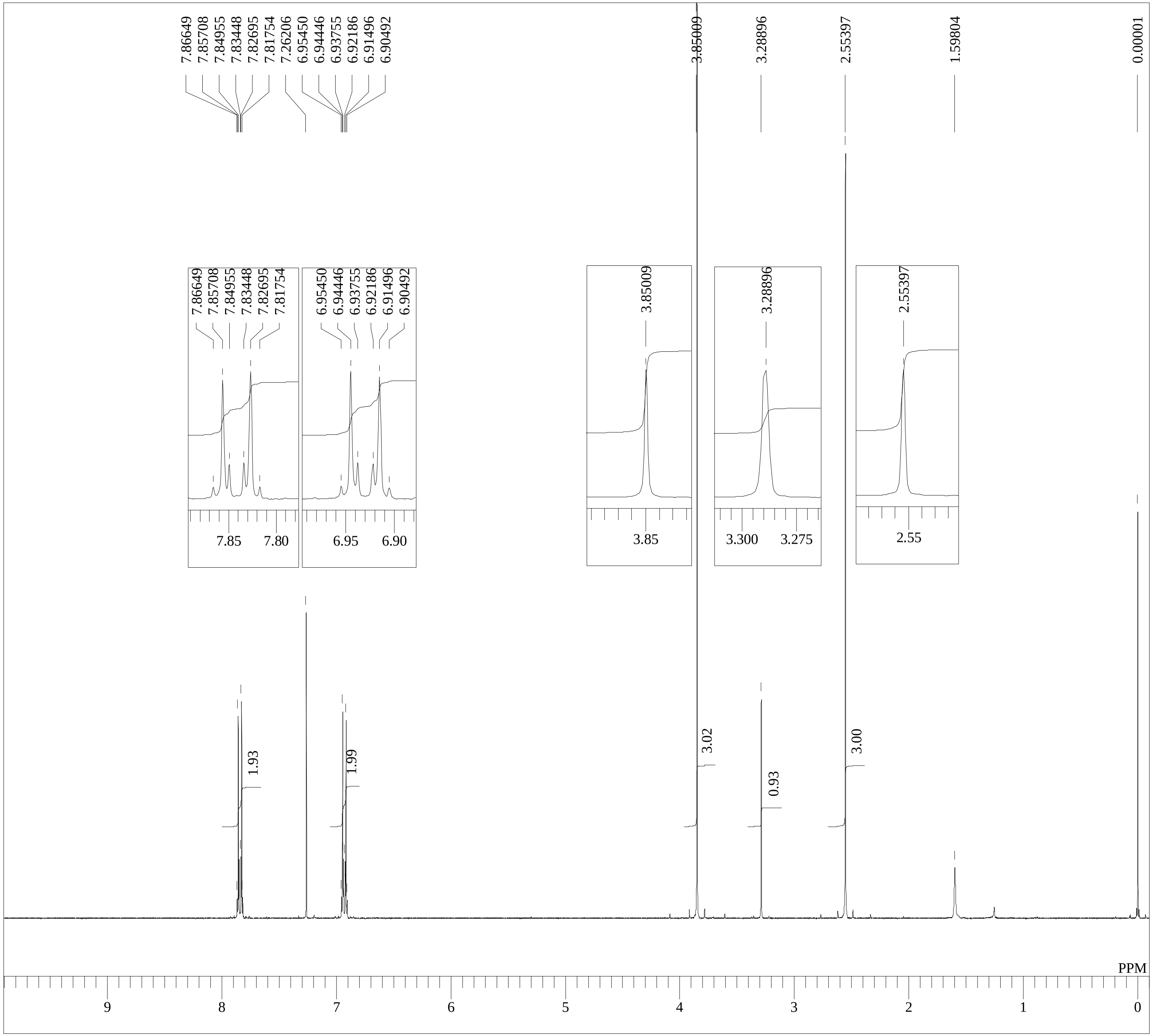

DFILE kose65-1.als
COMNT User YokoyamaYasushi_lab
DATIM 13:27:56.015 DRX300@NMRPC
OBNUC 1H
EXMOD zg30
OBFRQ 300.13 MHz
OBSET 1.89 KHz
OBFIN 10.00 Hz
POINT 32768
FREQU 6172.84 Hz
SCANS 8
ACQTM 5.3085 sec
PD 1.0000 sec
PW1 14.00 usec
IRNUC
CTEMP 27.8 c
SLVNT CDCl3
EXREF 16.59 ppm
BF 0.12 Hz
RGAIN 812

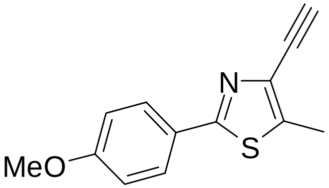

15

^{1}H NMR spectrum of compound **15** (300 MHz, $CDCl_3$, TMS)

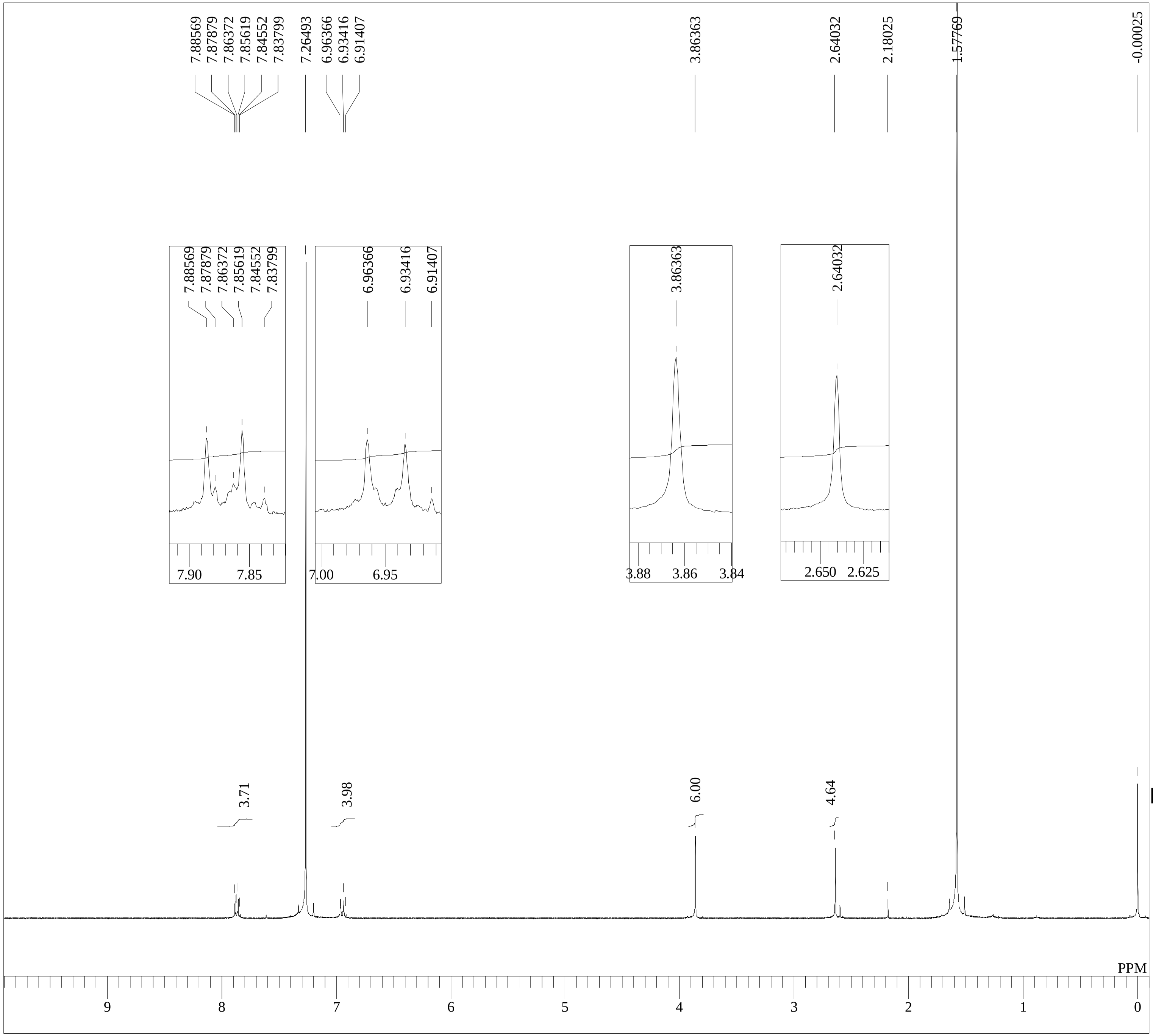

DFILE	Kose157-1.als
COMNT	User YokoyamaYasushi_lab
DATIM	11:06:04.328 DRX300@NMRPC
OBNUC	1H
EXMOD	zg30
OBFRQ	300.13 MHz
OBSET	1.89 KHz
OBFIN	10.00 Hz
POINT	32768
FREQU	6172.84 Hz
SCANS	236
ACQTM	5.3085 sec
PD	1.0000 sec
PW1	11.80 usec
IRNUC	
CTEMP	20.8 c
SLVNT	CDCl3
EXREF	16.60 ppm
BF	0.12 Hz
RGAIN	1448

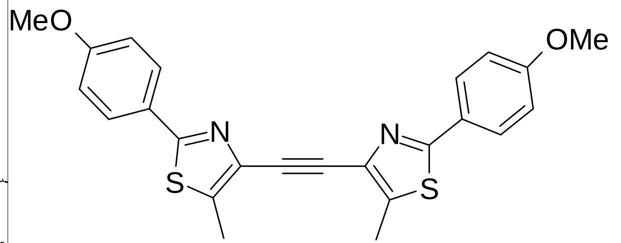

16

^{1}H NMR spectrum of compound **16** (300 MHz, $CDCl_3$, TMS)

DFILE	KOSE127-3-cry.als
COMNT	User YokoyamaYasushi_lab
DATIM	14:53:37.796 DRX300@NMRPC
OBNUC	1H
EXMOD	zg30
OBFRQ	300.13 MHz
OBSET	1.89 KHz
OBFIN	10.00 Hz
POINT	32768
FREQU	6172.84 Hz
SCANS	32
ACQTM	5.3085 sec
PD	1.0000 sec
PW1	11.80 usec
IRNUC	
CTEMP	25.0 c
SLVNT	CDCl3
EXREF	16.60 ppm
BF	0.12 Hz
RGAIN	1448

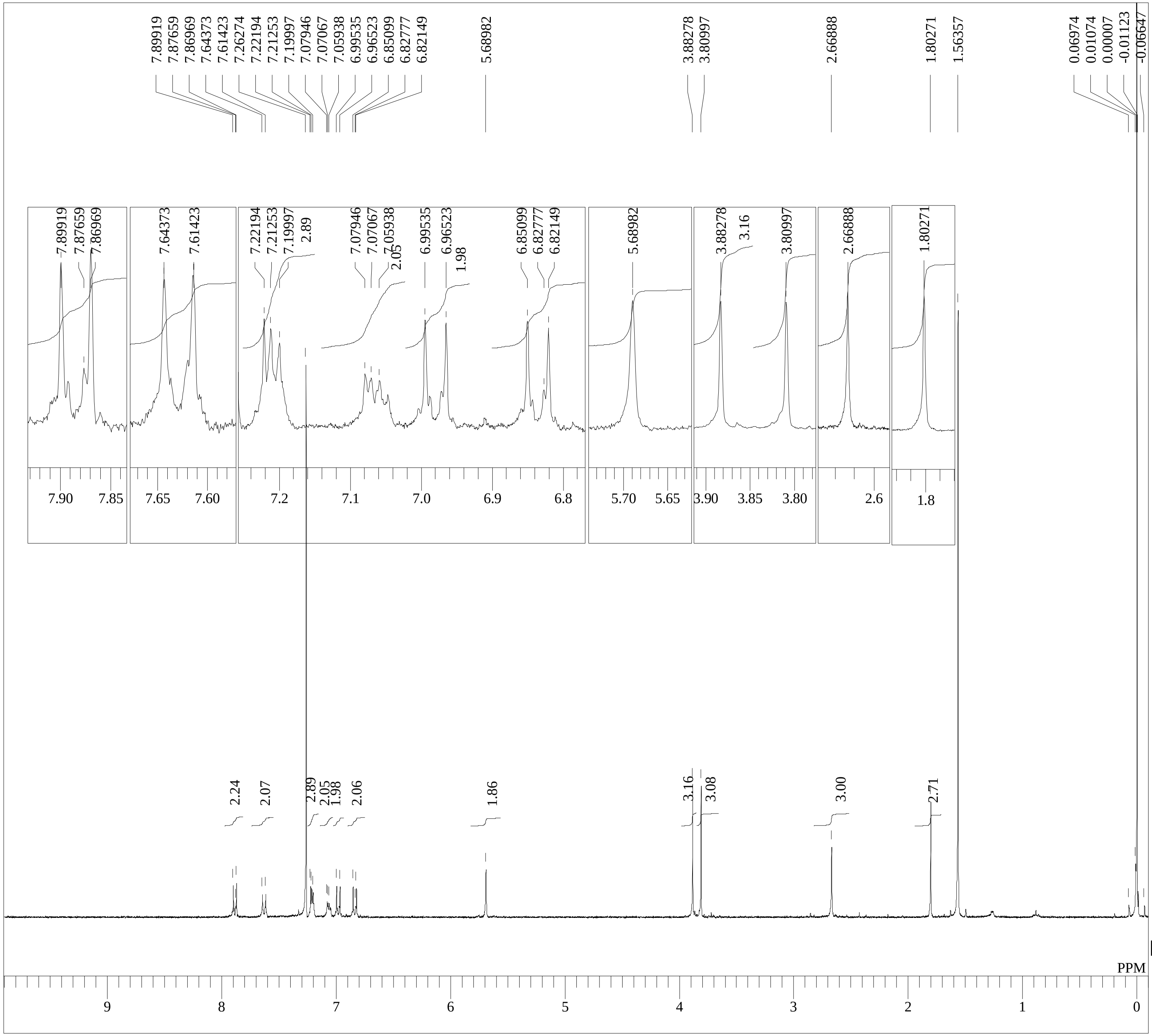

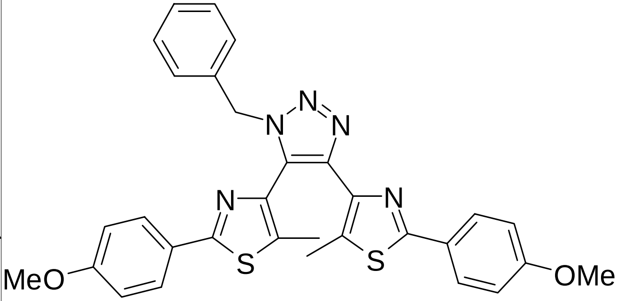

2o

^{1}H NMR spectrum of compound **2o** (300 MHz, $CDCl_3$, TMS)

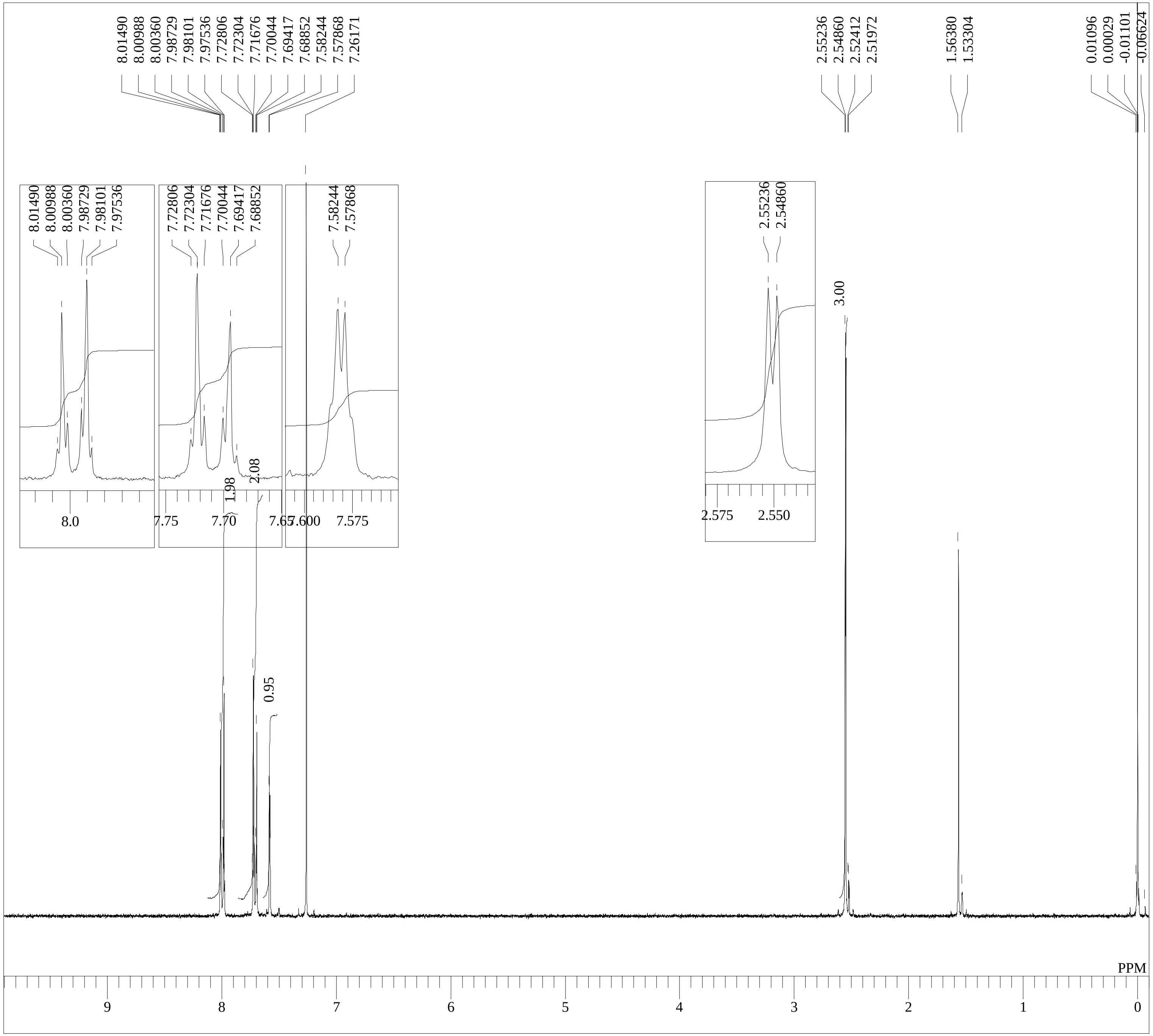

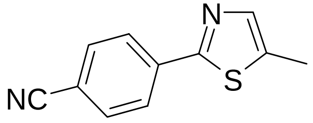

18

^{1}H NMR spectrum of compound **18** (300 MHz, $CDCl_3$, TMS)

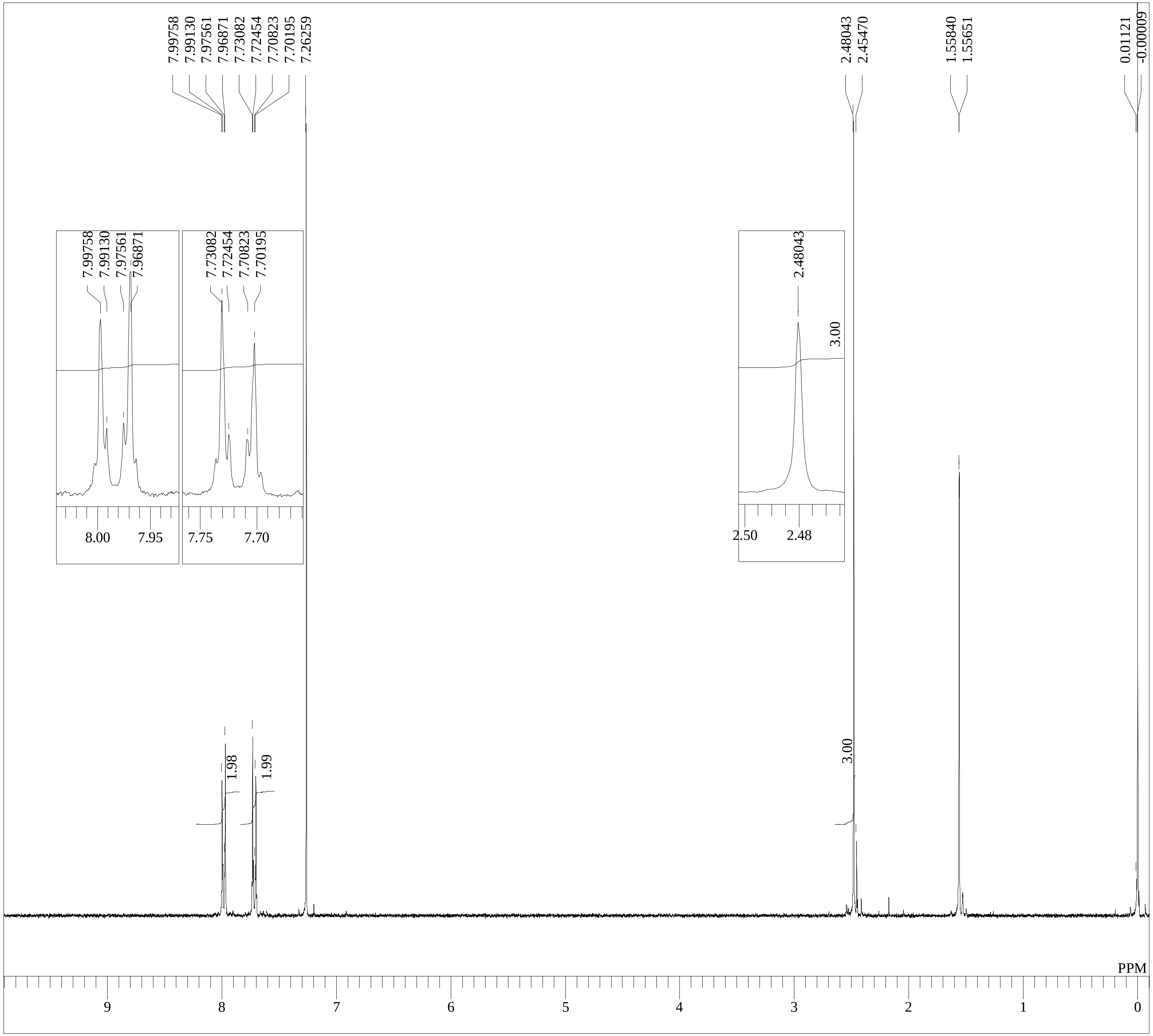

DFILE	BOO-3-142-1-cry.als
COMNT	User YokoyamaYasushi_lab
DATIM	08:34:58.921 DRX300@NMRPC
OBNUC	1H
EXMOD	zg30
OBFRQ	300.13 MHz
OBSET	1.89 KHz
OBFIN	10.00 Hz
POINT	32768
FREQU	6172.84 Hz
SCANS	8
ACQTM	5.3085 sec
PD	1.0000 sec
PW1	11.80 usec
IRNUC	
CTEMP	27.1 c
SLVNT	CDCl3
EXREF	16.59 ppm
BF	0.12 Hz
RGAIN	1625

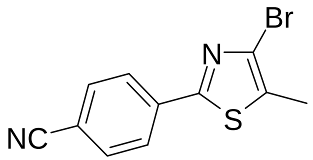

19

^{1}H NMR spectrum of compound **19** (300 MHz, $CDCl_3$, TMS)

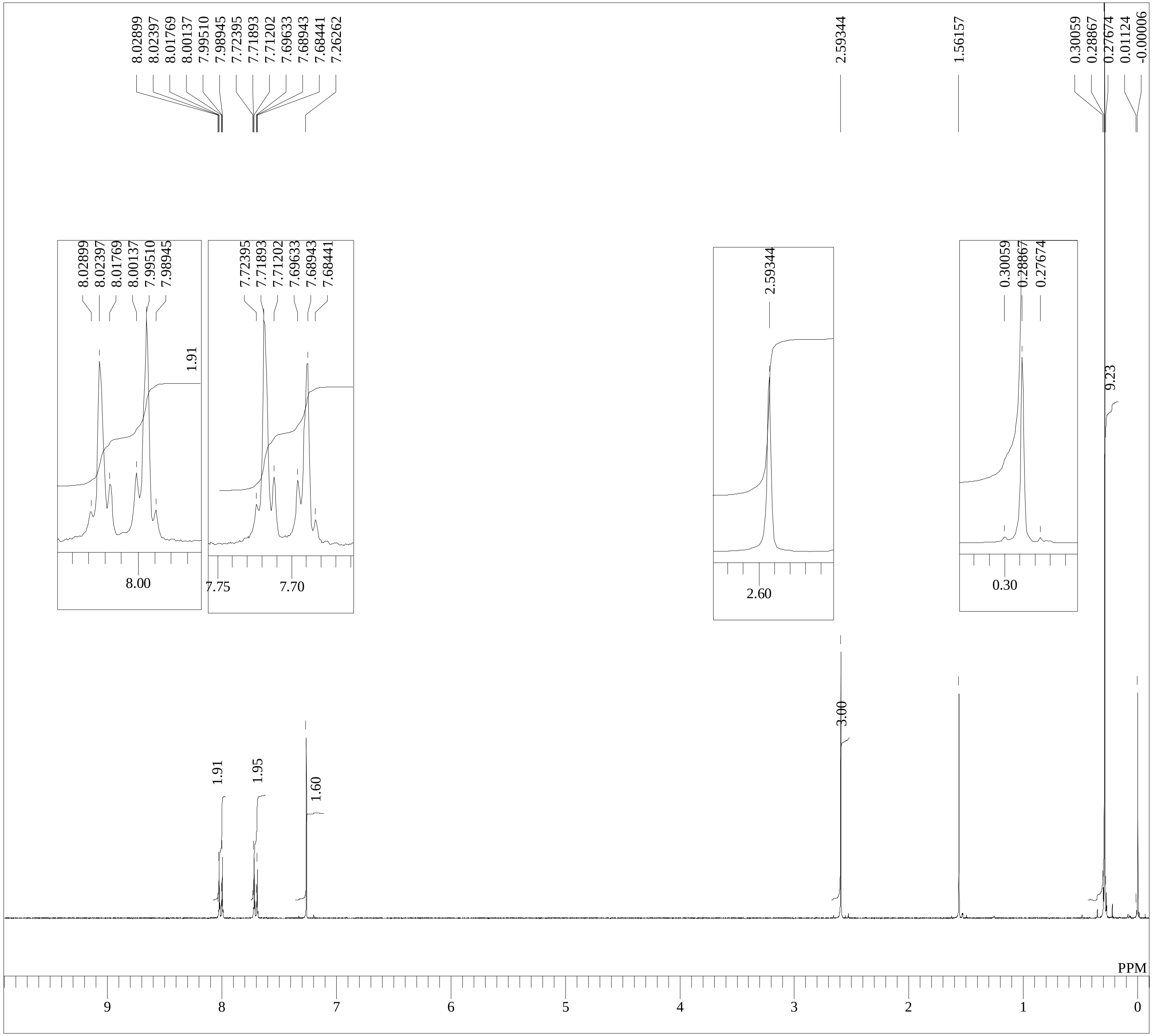

DFILE	BOO-3-120-1.als
COMNT	
DATIM	14:26:07.765 DRX300@NMRPC
OBNUC	1H
EXMOD	zg30
OBFRQ	300.13 MHz
OBSET	1.89 KHz
OBFIN	10.00 Hz
POINT	32768
FREQU	6172.84 Hz
SCANS	8
ACQTM	5.3085 sec
PD	1.0000 sec
PW1	11.80 usec
IRNUC	
CTEMP	28.4 c
SLVNT	CDCl3
EXREF	16.59 ppm
BF	0.12 Hz
RGAIN	1149

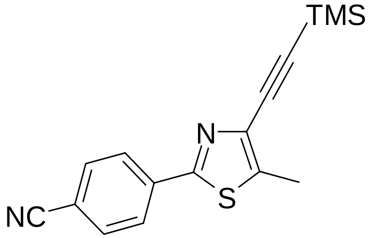

20

^{1}H NMR spectrum of compound **20** (300 MHz, $CDCl_3$, TMS)

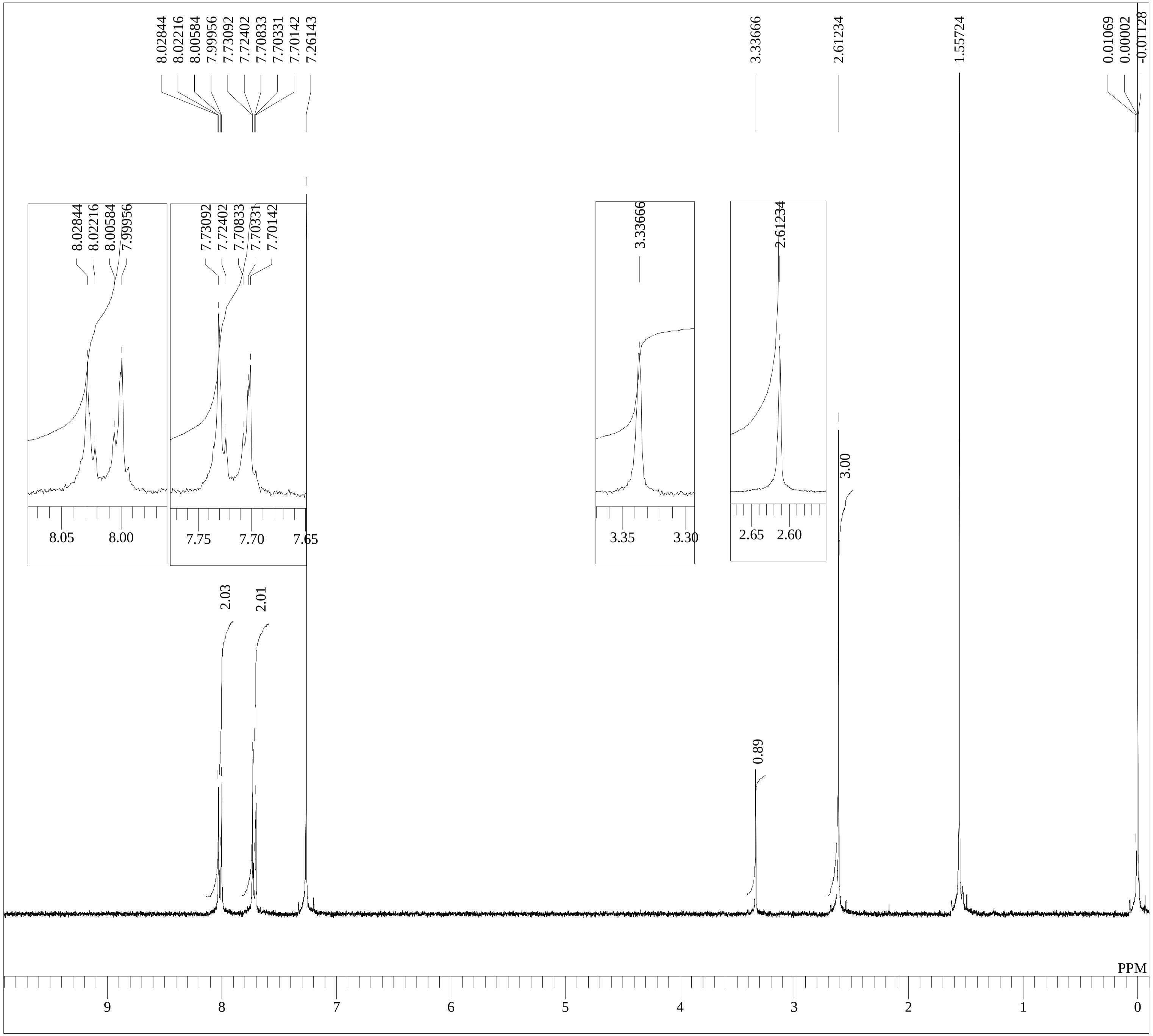

DFILE	BOO-1-122-1.als
COMNT	
DATIM	13:15:07.406 DRX300@NMRPC
OBNUC	1H
EXMOD	zg30
OBFRQ	300.13 MHz
OBSET	1.89 KHz
OBFIN	10.00 Hz
POINT	32768
FREQU	6172.84 Hz
SCANS	8
ACQTM	5.3085 sec
PD	1.0000 sec
PW1	11.80 usec
IRNUC	
CTEMP	27.4 c
SLVNT	CDCl3
EXREF	16.60 ppm
BF	0.12 Hz
RGAIN	1625

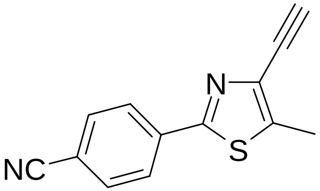

21

^{1}H NMR spectrum of compound **21** (300 MHz, $CDCl_3$, TMS)

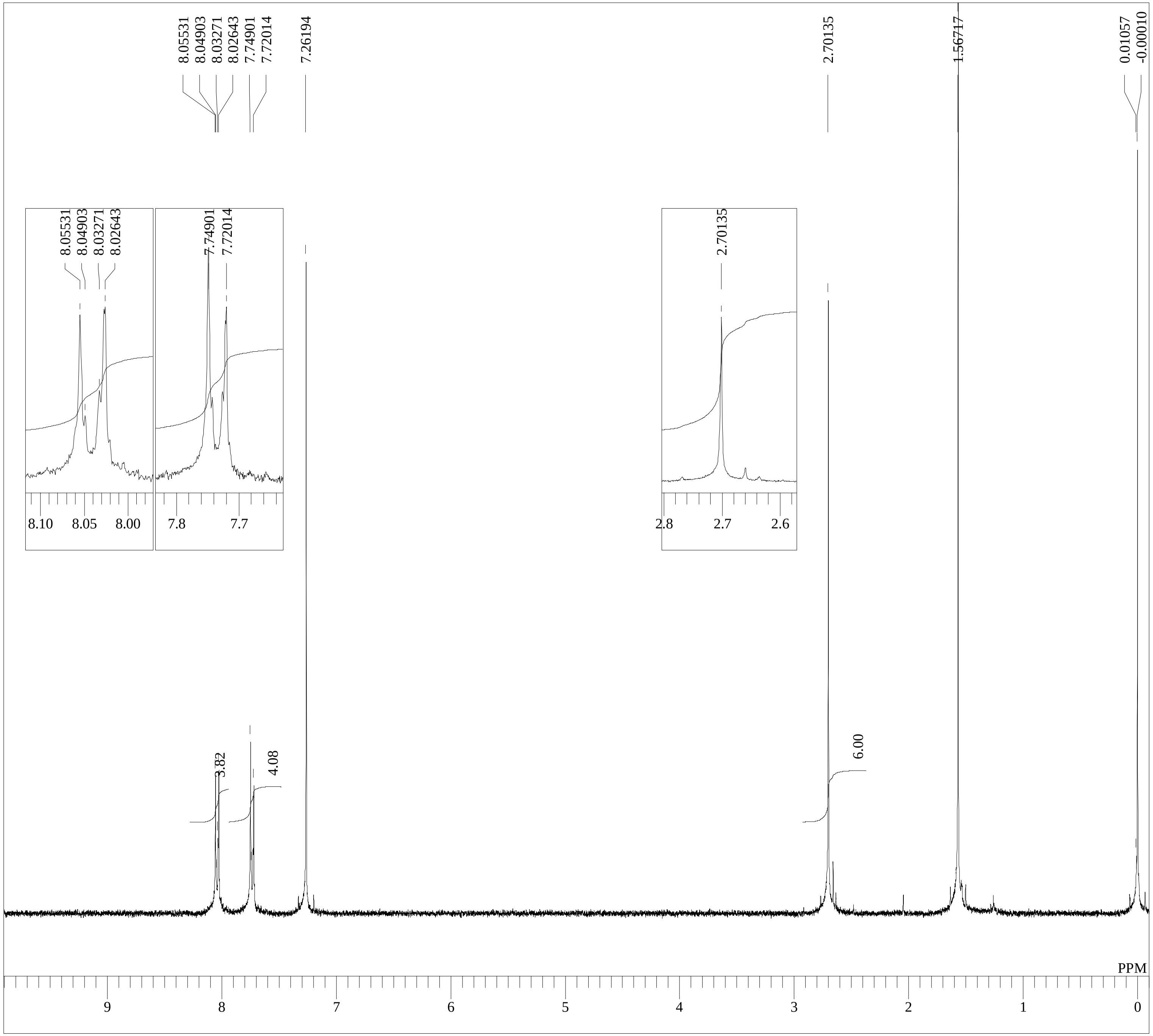

DFILE	BOO-3-130-4.als
COMNT	User YokoyamaYasushi_lab
DATIM	17:51:59.734 DRX300@NMRPC
OBNUC	1H
EXMOD	zg30
OBFRQ	300.13 MHz
OBSET	1.89 KHz
OBFIN	10.00 Hz
POINT	32768
FREQU	6172.84 Hz
SCANS	8
ACQTM	5.3085 sec
PD	1.0000 sec
PW1	11.80 usec
IRNUC	
CTEMP	28.0 c
SLVNT	CDCl3
EXREF	16.60 ppm
BF	0.12 Hz
RGAIN	1824

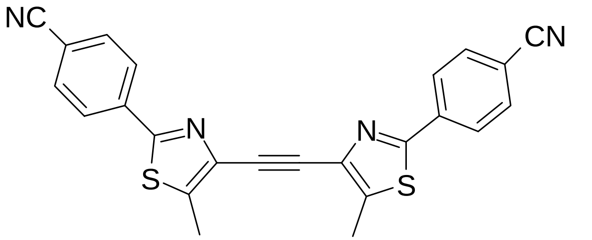

22

^{1}H NMR spectrum of compound **22** (300 MHz, $CDCl_3$, TMS)

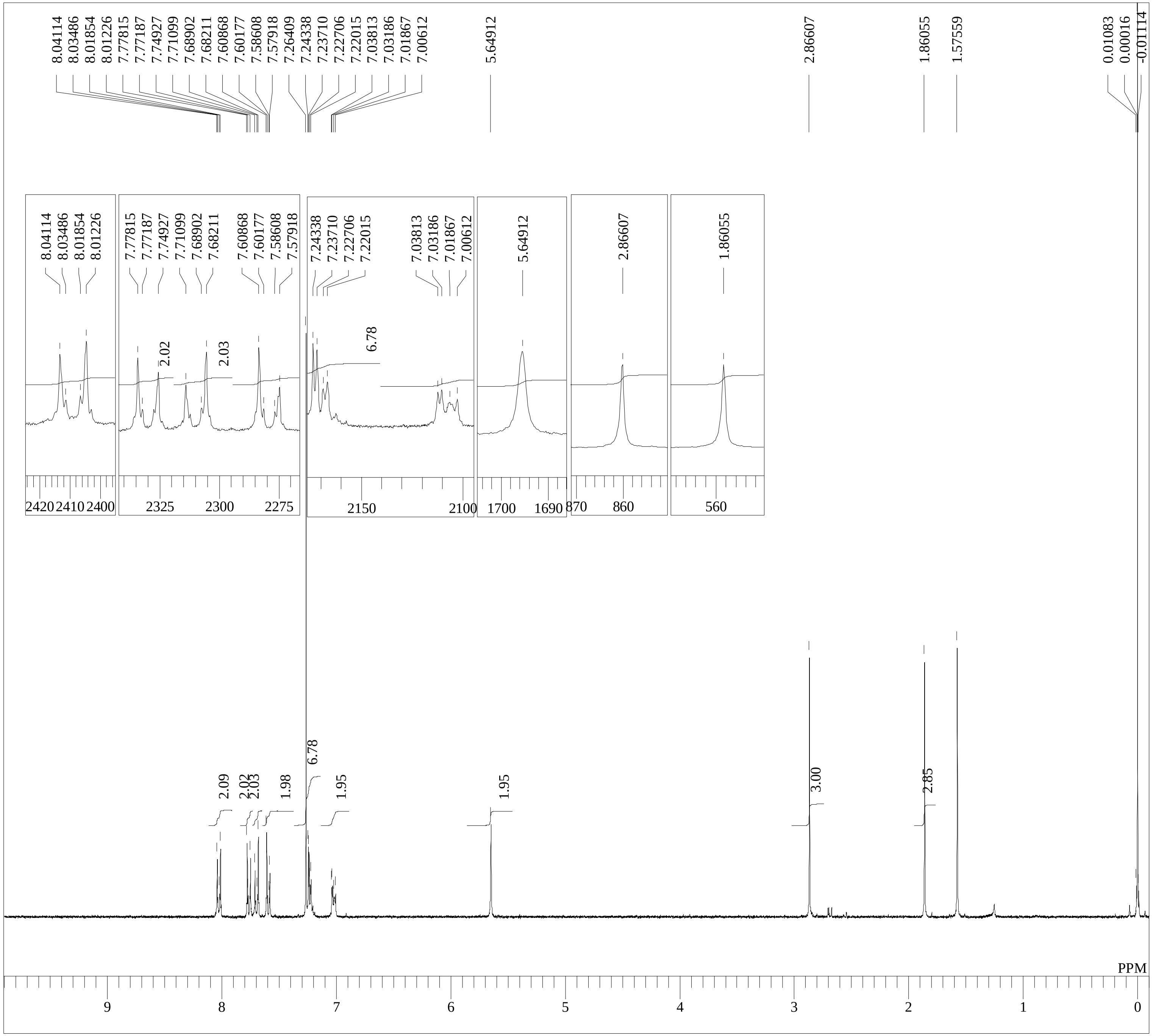

DFILE BOO-3-160-1 2nd.als
COMNT User YokoyamaYasushi_lab
DATIM 13:41:30.468 DRX300@NMRPC
OBNUC 1H
EXMOD zg30
OBFRQ 300.13 MHz
OBSET 1.89 KHz
OBFIN 10.00 Hz
POINT 32768
FREQU 6172.84 Hz
SCANS 8
ACQTM 5.3085 sec
PD 1.0000 sec
PW1 11.80 usec
IRNUC
CTEMP 21.8 c
SLVNT CDCl3
EXREF 16.60 ppm
BF 0.12 Hz
RGAIN 1149

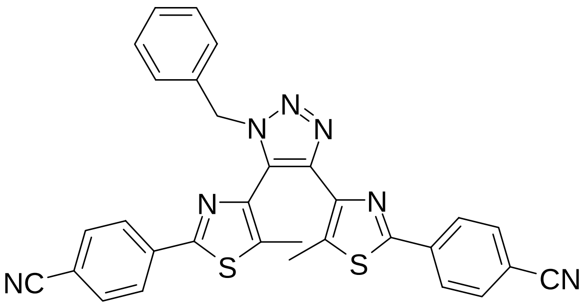

3o

^{1}H NMR spectrum of compound **3o** (300 MHz, $CDCl_3$, TMS)

SI-6. IR spectra

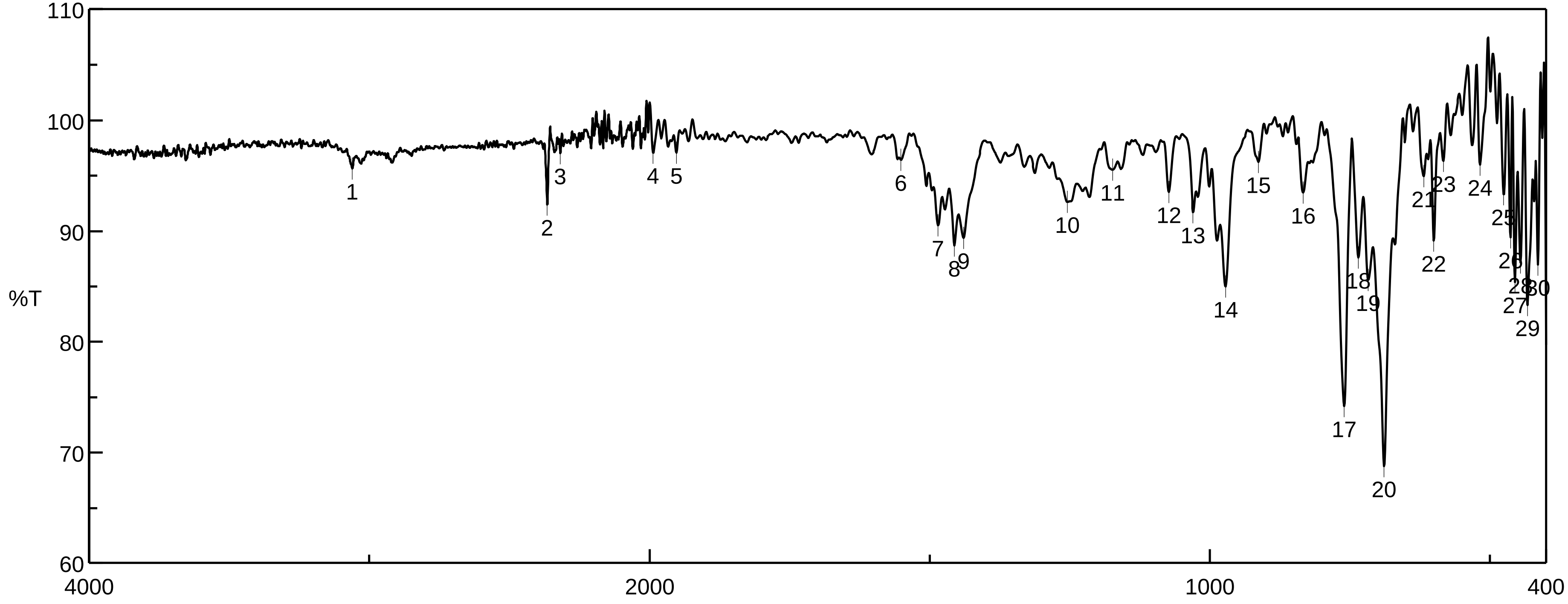

ピーク検出結果

No.	位置	強度	No.	位置	強度	No.	位置	強度	No.	位置	強度
1	3060.48	95.6049	2	2365.26	92.3415	3	2318.02	96.9849	4	1994.03	97.017
5	1951.61	97.0089	6	1551.45	96.3775	7	1484.92	90.4838	8	1455.99	88.6727
9	1439.6	89.3628	10	1254.47	92.5778	11	1173.47	95.4953	12	1073.19	93.4851
13	1029.8	91.6605	14	971.947	84.9731	15	913.129	96.203	16	833.098	93.4188
17	760.78	74.1466	18	734.746	87.5715	19	717.39	85.5403	20	689.427	68.7085
21	618.074	94.9105	22	600.717	89.115	23	583.361	96.28	24	517.793	95.9393
25	476.331	93.2637	26	463.796	89.4115	27	455.118	85.3593	28	445.476	87.1061
29	432.941	83.2816	30	414.62	86.9349						

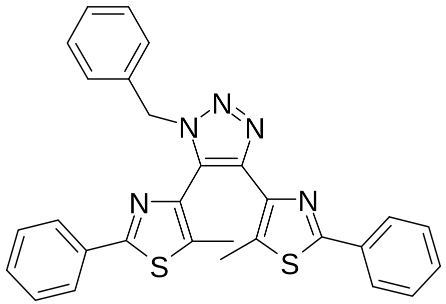

1o

IR spectrum of compound **1o** (ATR; diamond prism, neat)

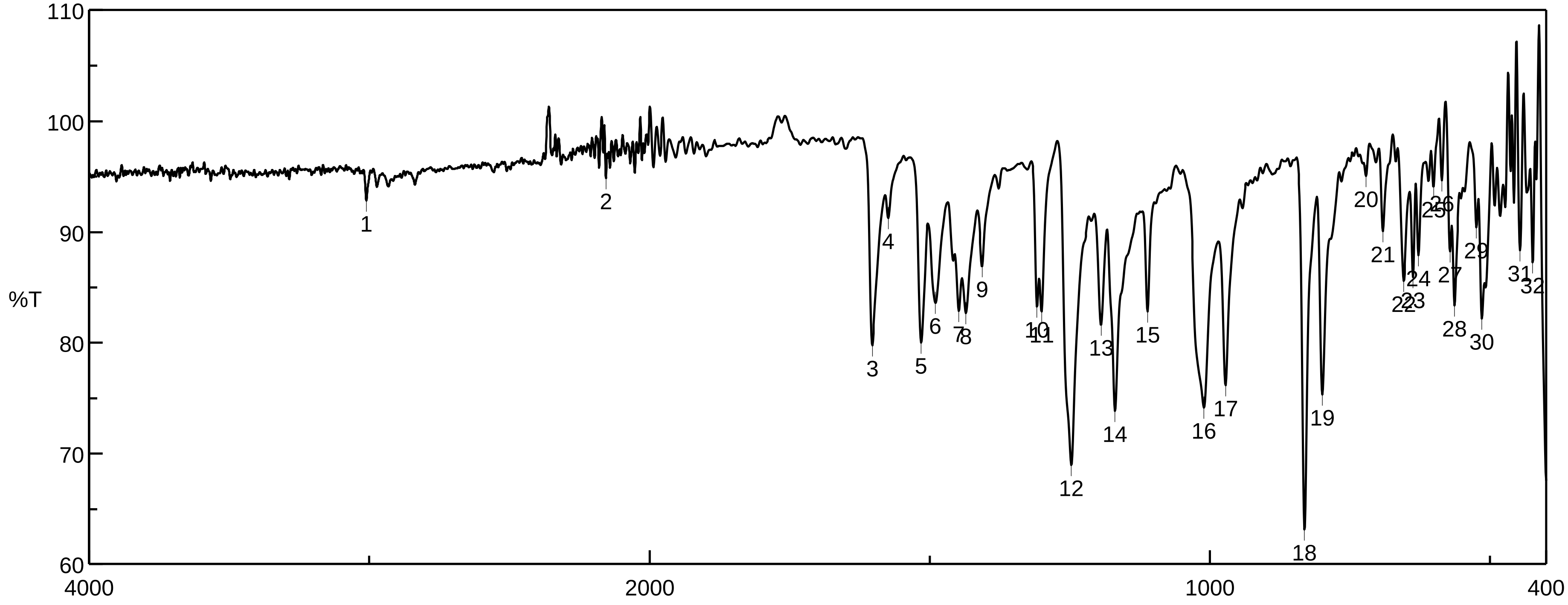

ピーク検出結果

No.	位置	強度	No.	位置	強度	No.	位置	強度	No.	位置	強度
1	3010.34	92.7846	2	2155.06	94.7896	3	1602.56	79.7406	4	1573.63	91.2115
5	1515.78	79.9827	6	1489.74	83.546	7	1448.28	82.8181	8	1435.74	82.6495
9	1405.85	86.8559	10	1308.46	83.2468	11	1299.79	82.7788	12	1247.72	68.903
13	1193.72	81.5811	14	1169.62	73.7879	15	1110.8	82.7955	16	1010.52	74.1047
17	971.947	76.1428	18	831.169	63.1227	19	799.35	75.2901	20	721.247	94.9992
21	691.355	90.0454	22	654.715	85.5637	23	637.358	85.8902	24	627.716	87.8433
25	600.717	94.0738	26	586.254	94.6218	27	571.79	88.1936	28	564.077	83.3299
29	524.543	90.3701	30	514.901	82.1585	31	446.44	88.2874	32	424.263	87.2159

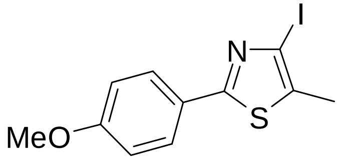

13

IR spectrum of compound **13** (ATR; diamond prism, neat)

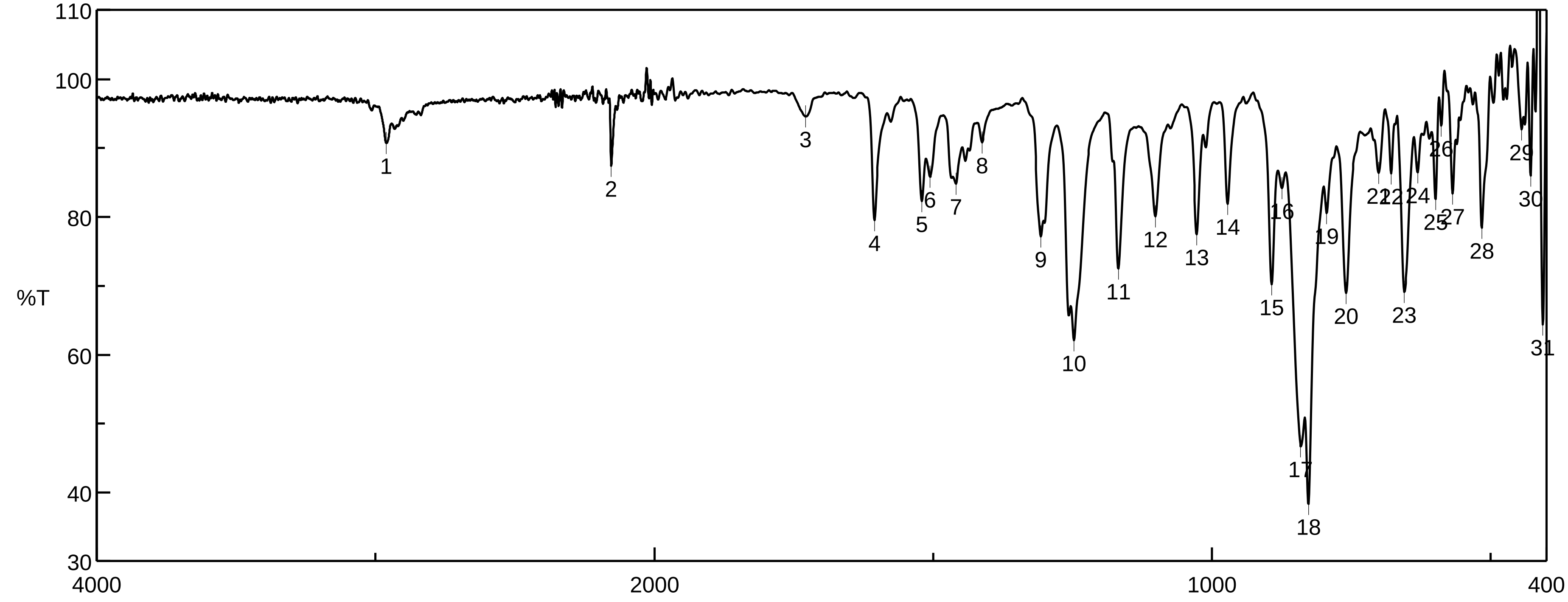

ピーク検出結果

No.	位置	強度	No.	位置	強度	No.	位置	強度	No.	位置	強度
1	2961.16	90.639	2	2154.1	87.3925	3	1728.87	94.5092	4	1605.45	79.4856
5	1520.6	82.2164	6	1506.13	85.7598	7	1458.89	84.724	8	1412.6	90.7708
9	1306.54	77.0983	10	1247.72	62.0095	11	1167.69	72.4603	12	1101.15	80.042
13	1026.91	77.3898	14	971.947	81.8235	15	892.88	70.1203	16	874.56	84.1361
17	840.812	46.6453	18	826.348	38.259	19	794.528	80.5224	20	759.816	68.8635
21	700.998	86.3691	22	678.82	86.2225	23	655.679	69.0886	24	630.609	86.3952
25	598.789	82.5333	26	589.147	93.2238	27	568.898	83.2991	28	515.865	78.3955
29	444.512	92.6413	30	428.12	85.9252	31	406.907	64.2817			

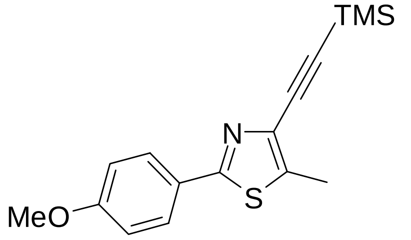

14

IR spectrum of compound **14** (ATR; diamond prism, neat)

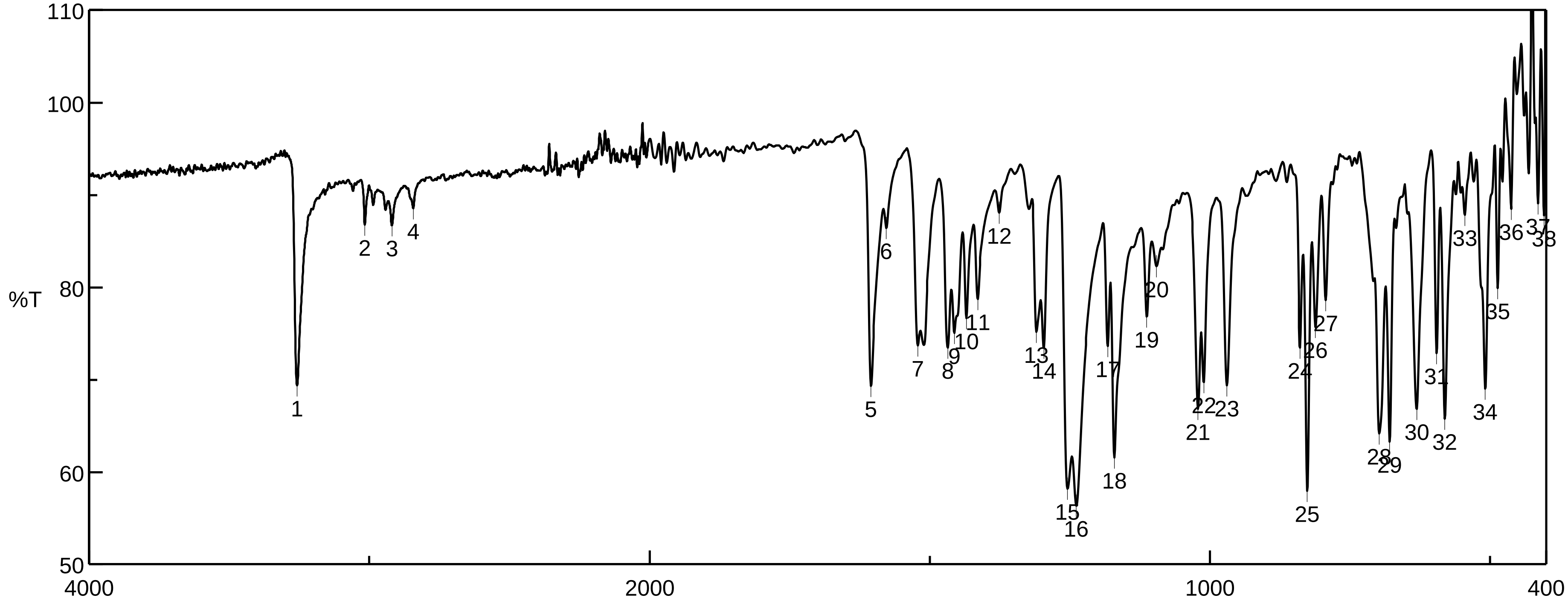

ピーク検出結果

No.	位置	強度	No.	位置	強度	No.	位置	強度	No.	位置	強度
1	3258.14	69.3435	2	3015.16	86.7468	3	2918.73	86.6626	4	2843.52	88.5334
5	1605.45	69.3143	6	1577.49	86.4106	7	1521.56	73.6687	8	1467.56	73.4253
9	1455.99	75.0266	10	1434.78	76.6541	11	1414.53	78.7165	12	1375.96	88.0187
13	1309.43	75.1702	14	1295.93	73.4241	15	1254.47	58.1366	16	1239.04	56.3106
17	1182.15	73.5894	18	1170.58	61.5272	19	1112.73	76.7858	20	1095.37	82.2556
21	1021.12	66.7779	22	1010.52	69.6819	23	970.019	69.3465	24	838.883	73.4364
25	826.348	57.9269	26	811.885	75.6911	27	793.564	78.5622	28	698.105	64.1345
29	679.785	63.2584	30	630.609	66.7991	31	595.896	72.8657	32	581.433	65.7536
33	544.792	87.8148	34	509.115	68.9681	35	486.938	79.8604	36	462.832	88.4513
37	414.62	89.029	38	404.014	87.7258						

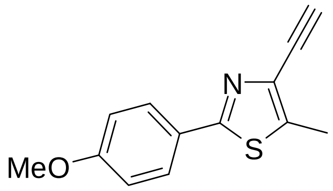

15

IR spectrum of compound **15** (ATR; diamond prism, neat)

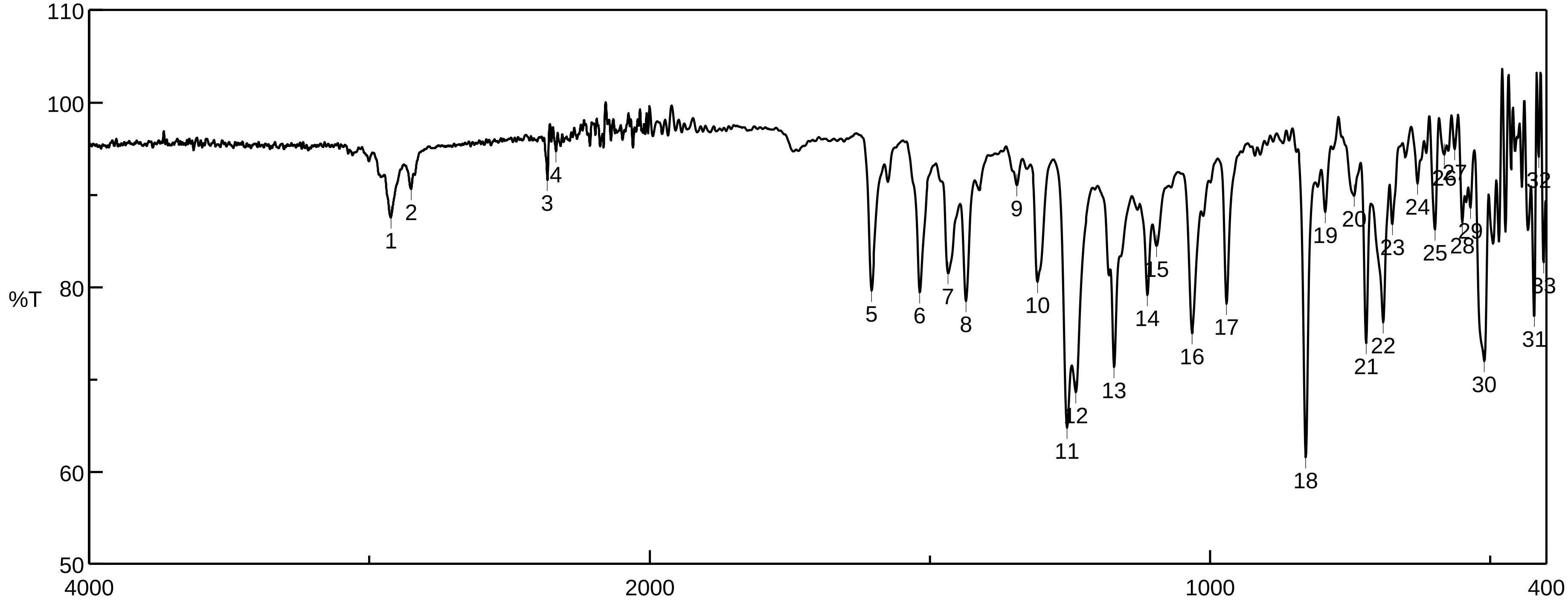

ピーク検出結果

No.	位置	強度	No.	位置	強度	No.	位置	強度	No.	位置	強度
1	2923.56	87.5076	2	2851.24	90.6128	3	2365.26	91.5723	4	2333.45	94.6684
5	1604.48	79.6122	6	1518.67	79.4043	7	1467.56	81.4901	8	1435.74	78.4743
9	1345.11	90.9947	10	1307.5	80.5171	11	1255.43	64.755	12	1240	68.6059
13	1171.54	71.3015	14	1111.76	79.1128	15	1095.37	84.42	16	1032.69	74.9944
17	970.983	78.1743	18	829.241	61.5293	19	794.528	88.1156	20	742.46	89.8817
21	721.247	73.9297	22	691.355	76.1527	23	674.963	86.8057	24	629.644	91.2019
25	598.789	86.2196	26	582.397	94.3437	27	564.077	94.9356	28	549.613	87.0019
29	535.15	88.5988	30	511.044	71.9722	31	421.37	76.8547	32	413.656	94.0912
33	404.978	82.6881									

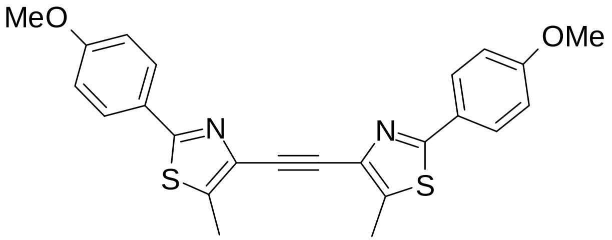

16

IR spectrum of compound **16** (ATR; diamond prism, neat)

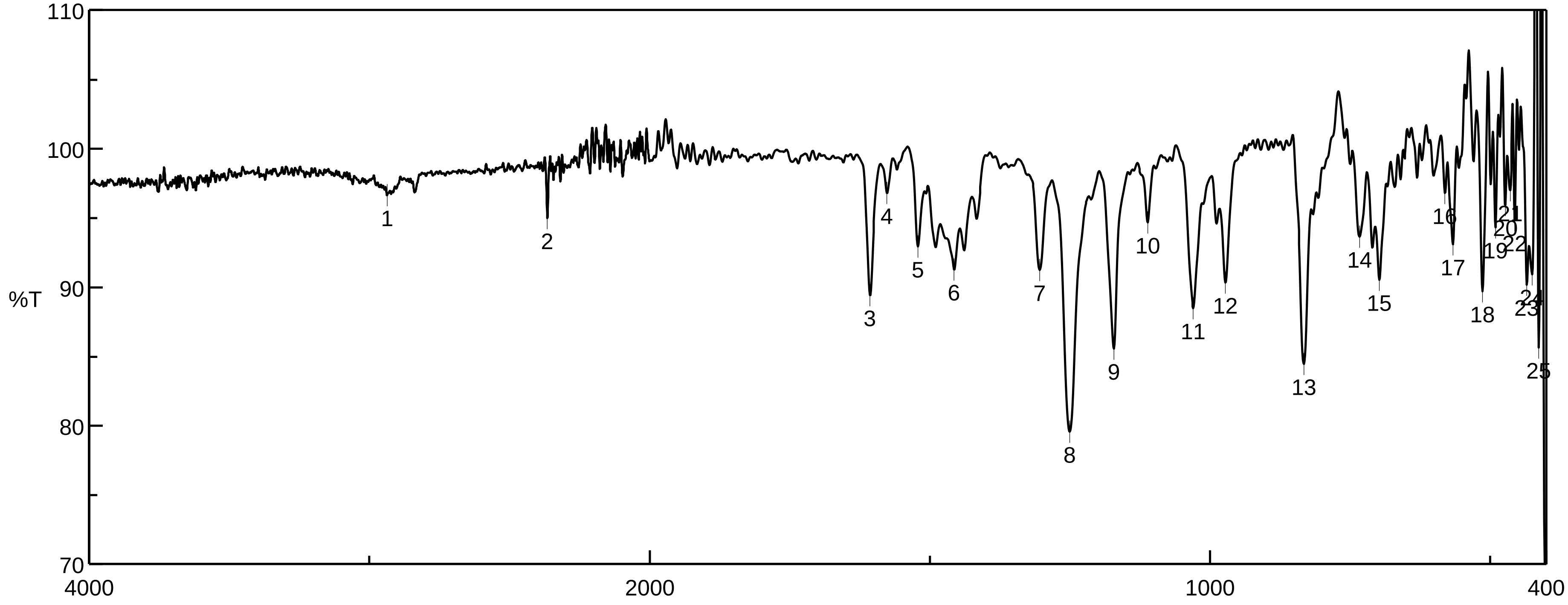

ピーク検出結果

No.	位置	強度	No.	位置	強度	No.	位置	強度	No.	位置	強度
1	2937.06	96.6298	2	2365.26	94.9566	3	1607.38	89.3957	4	1576.52	96.7891
5	1521.56	92.9216	6	1456.96	91.2713	7	1303.64	91.2407	8	1250.61	79.5367
9	1171.54	85.5339	10	1110.8	94.6727	11	1029.8	88.4598	12	972.912	90.3035
13	832.133	84.4562	14	732.817	93.6367	15	698.105	90.4953	16	581.433	96.774
17	566.969	93.0787	18	513.936	89.6902	19	490.795	94.311	20	473.439	95.9226
21	464.761	96.979	22	456.082	94.8183	23	434.869	90.1602	24	425.227	90.9185
25	413.656	85.6334									

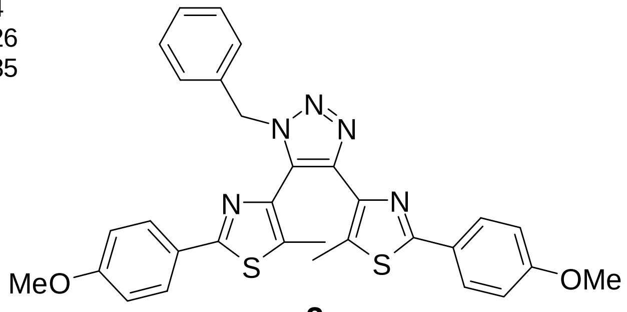

2o

IR spectrum of compound **2o** (ATR; diamond prism, neat)

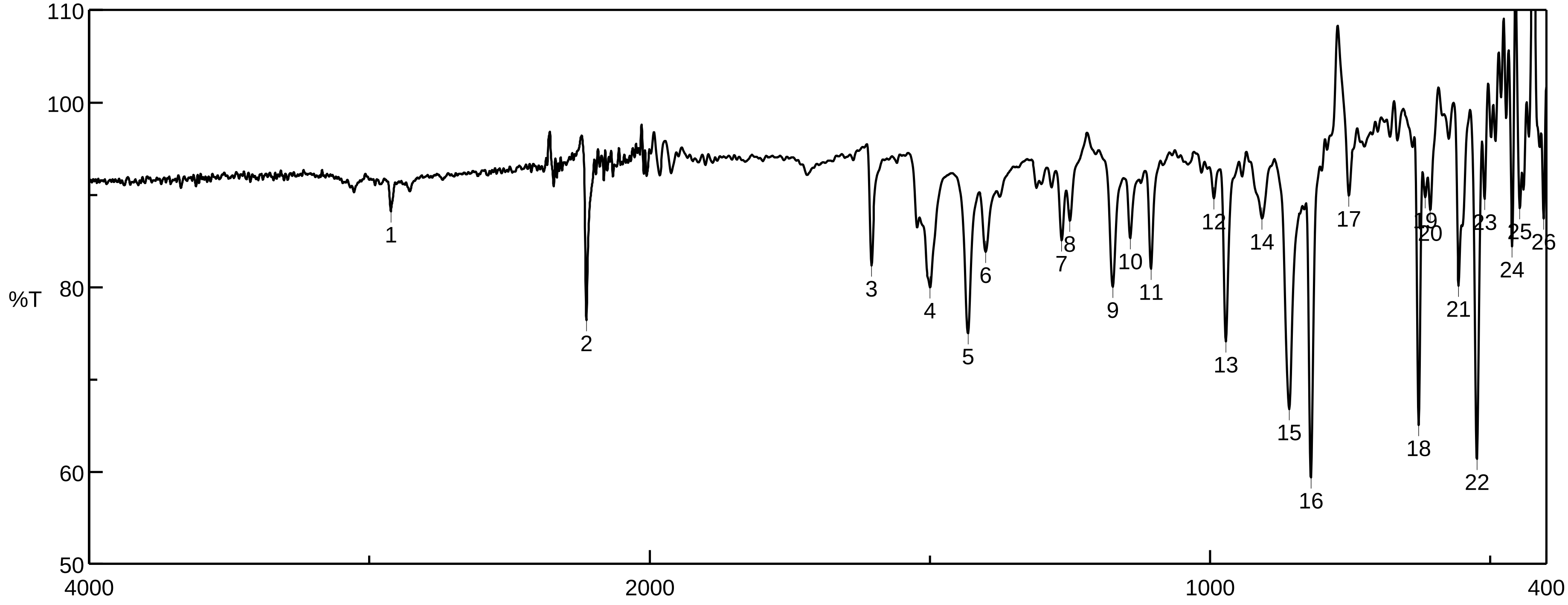

ピーク検出結果

No.	位置	強度	No.	位置	強度	No.	位置	強度	No.	位置	強度
1	2923.56	88.1951	2	2225.45	76.4122	3	1604.48	82.293	4	1499.38	79.9653
5	1431.89	74.9961	6	1400.07	83.7751	7	1265.07	85.0494	8	1250.61	87.1767
9	1173.47	79.9967	10	1142.62	85.2738	11	1105.01	81.9697	12	993.16	89.5944
13	971.947	74.0742	14	907.344	87.4029	15	859.132	66.771	16	819.598	59.3289
17	753.066	89.8742	18	627.716	65.0543	19	616.145	89.7049	20	607.467	88.3605
21	556.363	80.1198	22	523.579	61.3818	23	510.08	89.5328	24	460.904	84.3765
25	447.404	88.5436	26	404.978	87.4259						

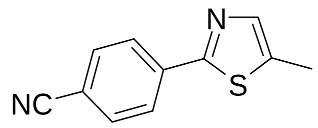

18

IR spectrum of compound **18** (ATR; diamond prism, neat)

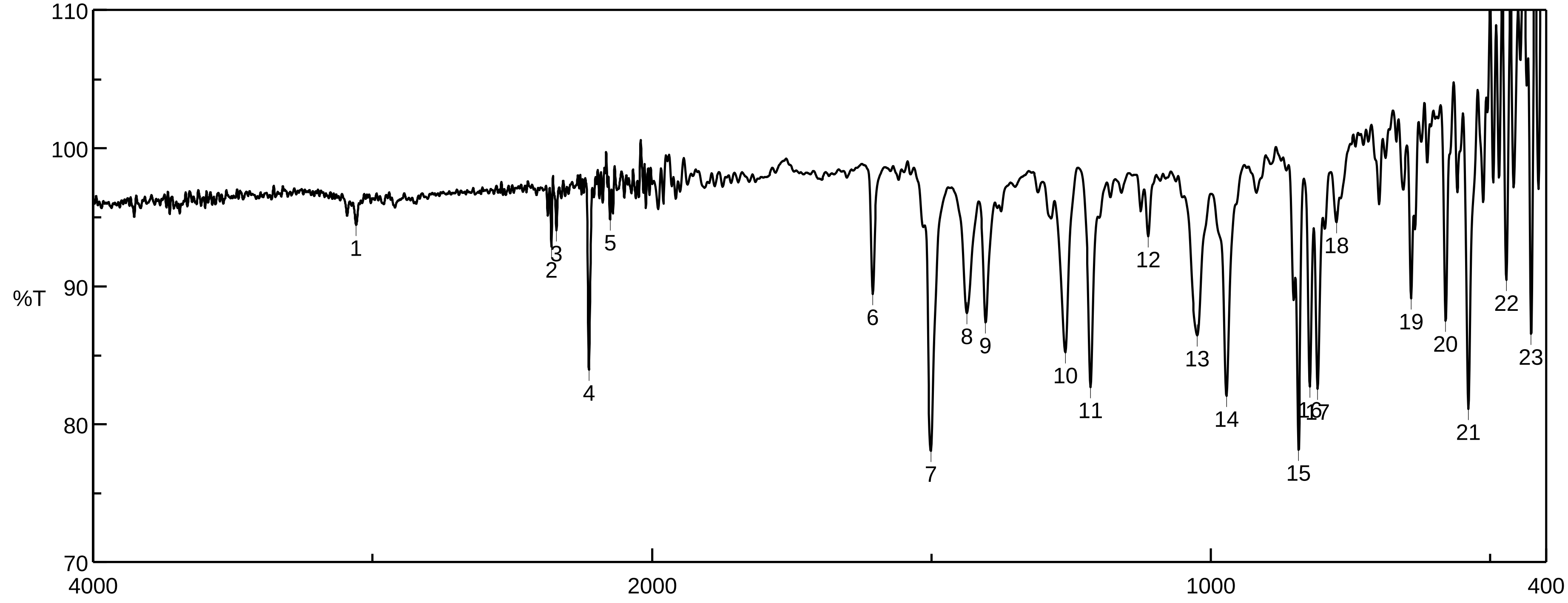

ピーク検出結果

No.	位置	強度	No.	位置	強度	No.	位置	強度	No.	位置	強度
1	3058.55	94.4196	2	2360.44	92.8535	3	2341.16	94.0144	4	2225.45	83.9257
5	2149.28	94.8064	6	1605.45	89.4263	7	1500.35	78.0334	8	1436.71	88.0145
9	1402.96	87.3594	10	1260.25	85.1891	11	1214.93	82.6677	12	1111.76	93.6042
13	1024.02	86.4053	14	971.947	82.0291	15	843.704	78.1163	16	822.491	82.6875
17	808.992	82.5614	18	775.244	94.6302	19	641.215	89.1091	20	580.469	87.4653
21	539.007	81.0666	22	471.51	90.4422	23	427.155	86.5438			

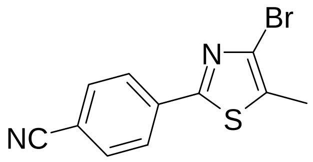

19

IR spectrum of compound **19** (ATR; diamond prism, neat)

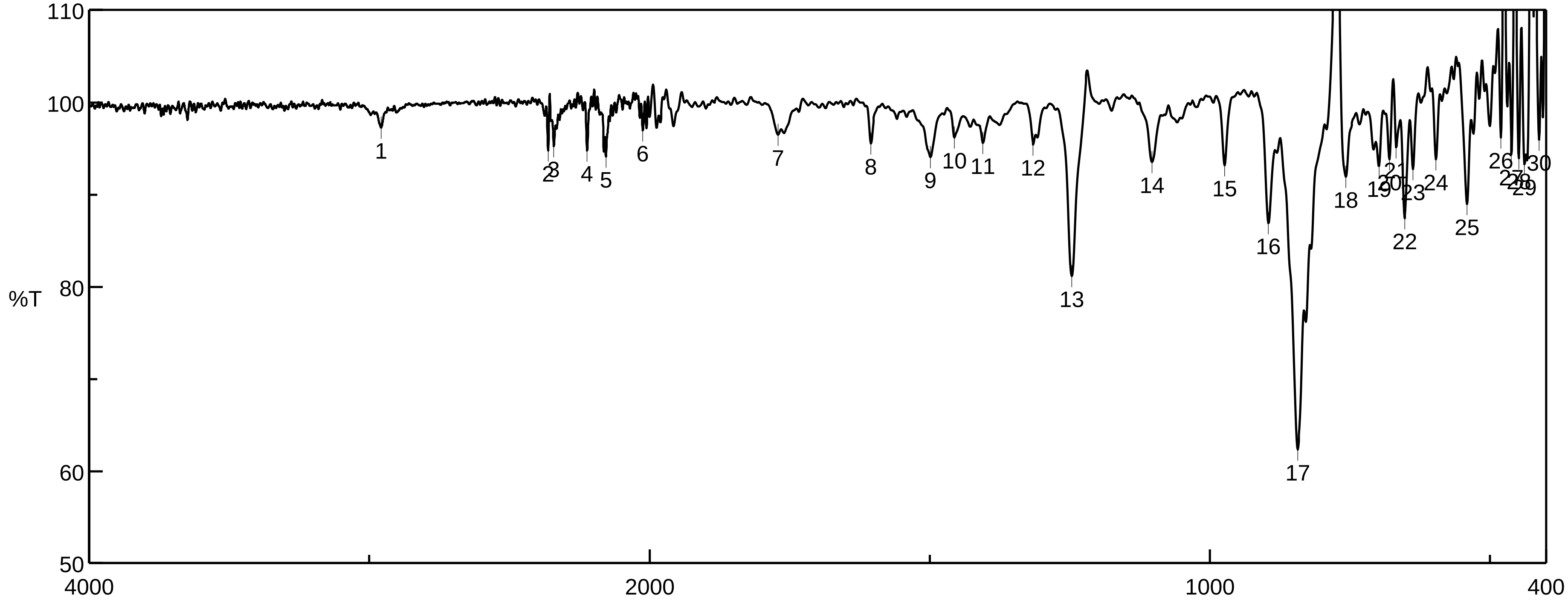

ピーク検出結果

No.	位置	強度	No.	位置	強度	No.	位置	強度	No.	位置	強度
1	2958.27	97.2382	2	2362.37	94.7538	3	2341.16	95.2324	4	2222.56	94.7313
5	2155.06	94.0995	6	2023.93	96.9524	7	1770.33	96.4578	8	1605.45	95.5166
9	1498.42	94.0479	10	1455.99	96.1779	11	1404.89	95.5862	12	1315.21	95.3763
13	1246.75	81.1285	14	1103.08	93.5075	15	973.876	93.1469	16	895.773	86.8611
17	843.704	62.3207	18	757.888	91.8788	19	698.105	93.0488	20	679.785	93.8051
21	668.214	95.1026	22	652.786	87.4208	23	637.358	92.7081	24	596.861	93.8099
25	540.935	88.9326	26	481.153	96.1609	27	461.868	94.3421	28	448.369	93.8981
29	438.726	93.242	30	412.692	95.8983						

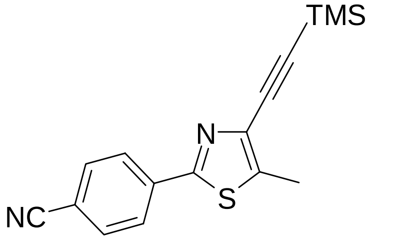

20

IR spectrum of compound **20** (ATR; diamond prism, neat)

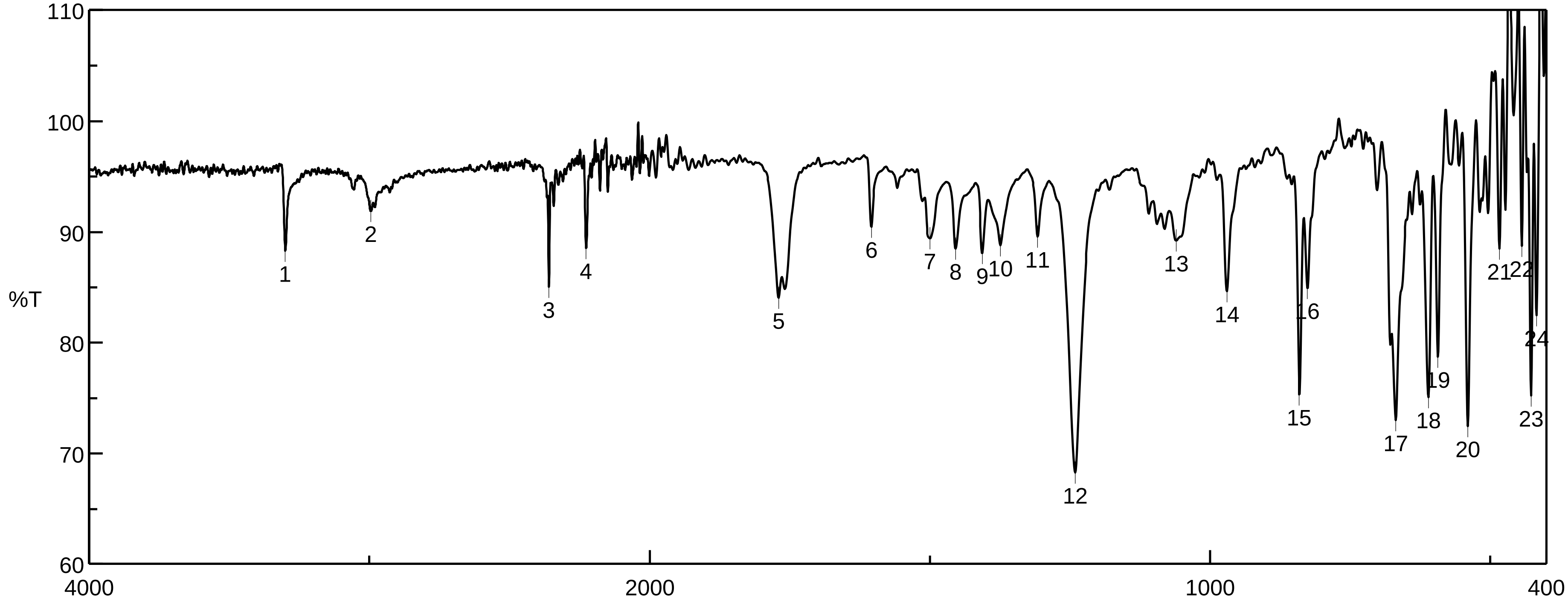

ピーク検出結果

No.	位置	強度	No.	位置	強度	No.	位置	強度	No.	位置	強度
1	3299.61	88.278	2	2993.94	91.8375	3	2359.48	85.0187	4	2226.42	88.4928
5	1769.37	83.9979	6	1604.48	90.4038	7	1499.38	89.3723	8	1454.06	88.4477
9	1405.85	88.0498	10	1374.03	88.7653	11	1307.5	89.5163	12	1240.97	68.2312
13	1060.66	89.1691	14	970.019	84.6216	15	840.812	75.3066	16	826.348	84.9053
17	669.178	72.9899	18	610.36	75.058	19	593.968	78.7013	20	539.971	72.4321
21	484.045	88.4557	22	443.547	88.7115	23	427.155	75.1834	24	417.513	82.439

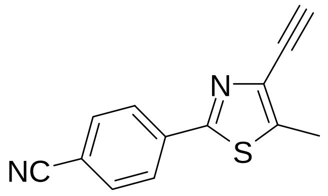

21

IR spectrum of compound **21** (ATR; diamond prism, neat)

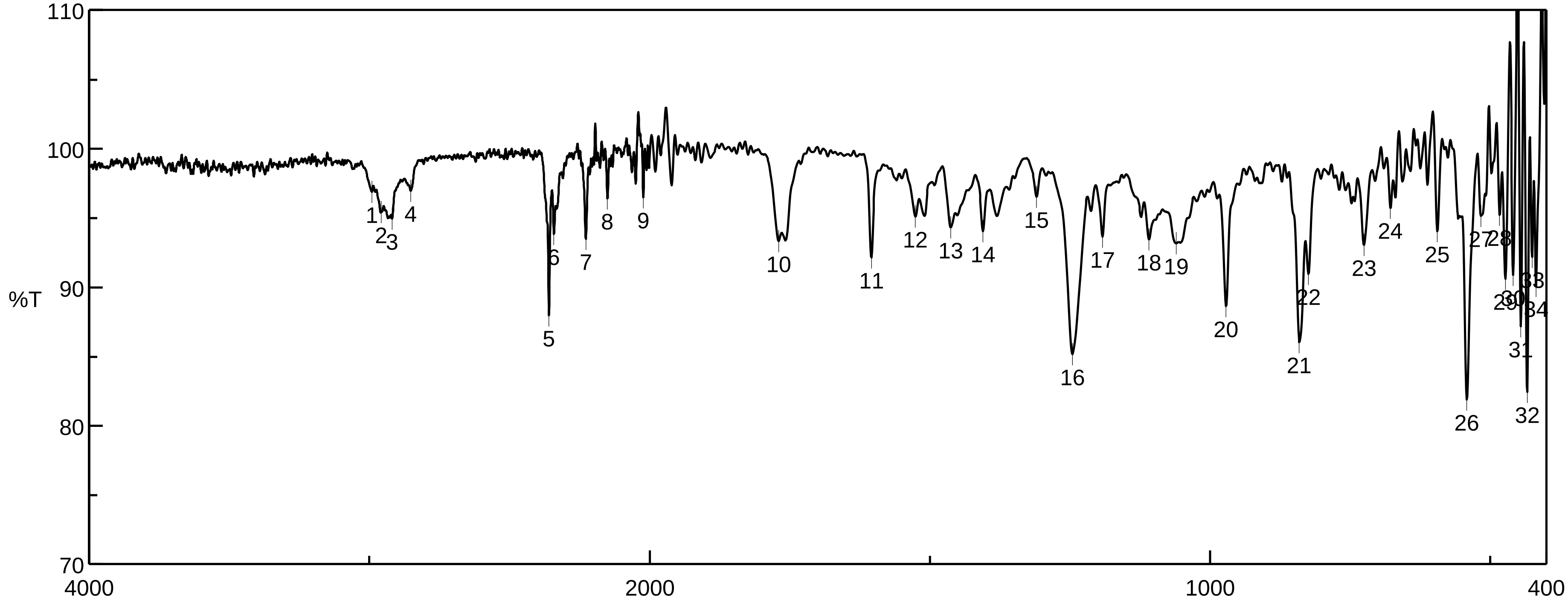

ピーク検出結果

No.	位置	強度	No.	位置	強度	No.	位置	強度	No.	位置	強度
1	2991.05	96.8791	2	2958.27	95.3929	3	2918.73	94.9128	4	2852.2	96.952
5	2359.48	87.9661	6	2341.16	93.8369	7	2227.38	93.4737	8	2150.24	96.3997
9	2022.96	96.4733	10	1769.37	93.3076	11	1604.48	92.1339	12	1526.38	95.076
13	1462.74	94.298	14	1404.89	94.0373	15	1309.43	96.5198	16	1245.79	85.1535
17	1191.79	93.6229	18	1108.87	93.4211	19	1060.66	93.1499	20	971.947	88.6146
21	840.812	85.9991	22	824.42	90.9295	23	725.104	93.0321	24	678.82	95.7304
25	594.932	94.0142	26	541.899	81.8865	27	516.829	95.1165	28	484.045	95.2083
29	473.439	90.5879	30	458.975	90.8676	31	445.476	87.1635	32	433.905	82.4166
33	425.227	92.1802	34	418.477	90.0927						

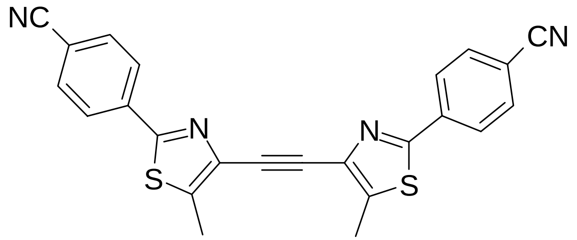

22

IR spectrum of compound **22** (ATR; diamond prism, neat)

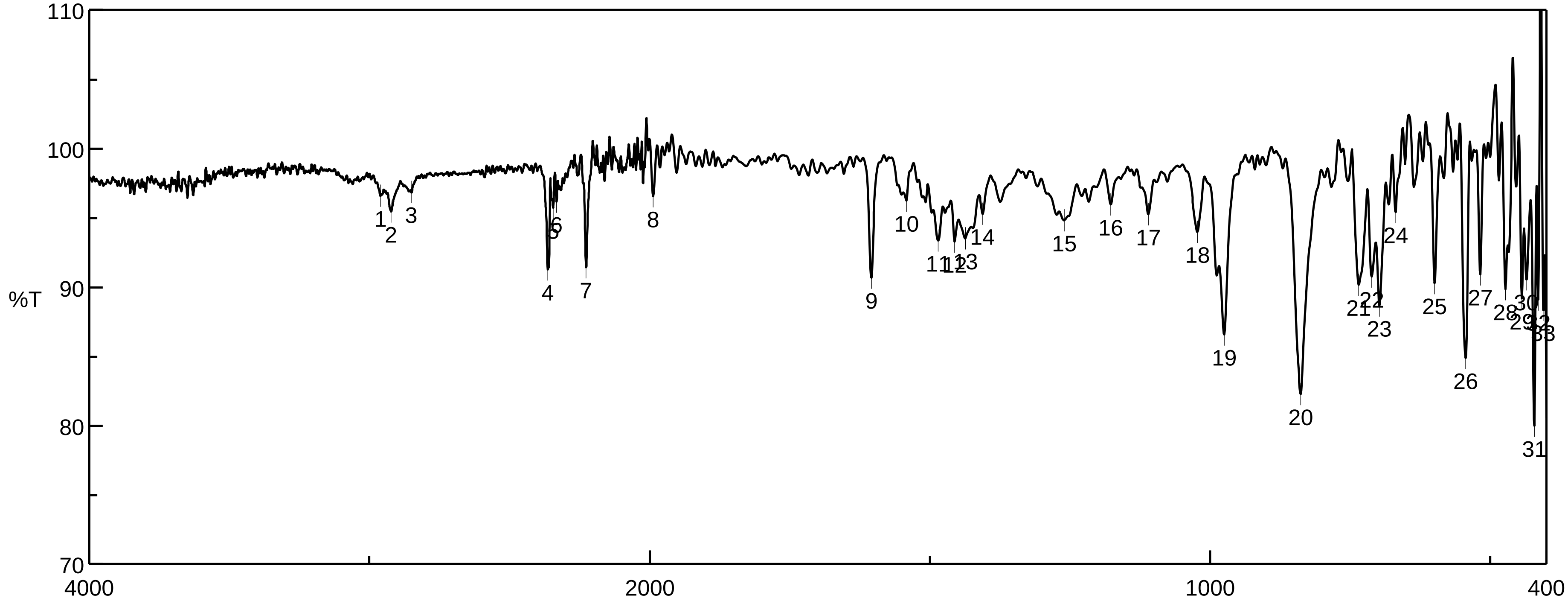

ピーク検出結果

No.	位置	強度	No.	位置	強度	No.	位置	強度	No.	位置	強度
1	2960.2	96.5694	2	2922.59	95.4382	3	2851.24	96.8826	4	2363.34	91.2516
5	2344.05	95.7332	6	2331.52	96.1709	7	2226.42	91.4092	8	1994.03	96.5624
9	1604.48	90.6692	10	1541.81	96.2488	11	1484.92	93.3573	12	1455.99	93.2877
13	1436.71	93.53	14	1405.85	95.303	15	1260.25	94.8272	16	1177.33	95.9451
17	1109.83	95.246	18	1022.09	93.9693	19	974.84	86.5796	20	837.919	82.2513
21	734.746	90.1572	22	711.604	90.756	23	698.105	88.6471	24	669.178	95.4049
25	599.753	90.2622	26	543.828	84.8709	27	517.793	90.9193	28	473.439	89.8533
29	443.547	89.1664	30	435.834	90.5517	31	421.37	79.9747	32	414.62	89.0837
33	405.942	88.362									

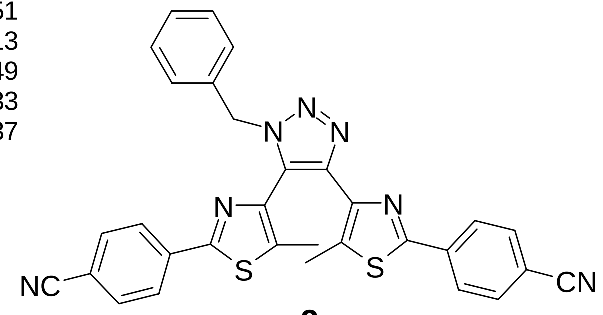

3o

IR spectrum of compound **3o** (ATR; diamond prism, neat)

SI-7. Mass spectra

2009/11/27

Page 1

File: YOK-BOO-3-174-1
Sample: Description
Instrument: JEOL MSRoute
Inlet: Direct Probe

Date Run: 2009-11-27 (Time Run: 10:19:32)

Ionization mode: EI+

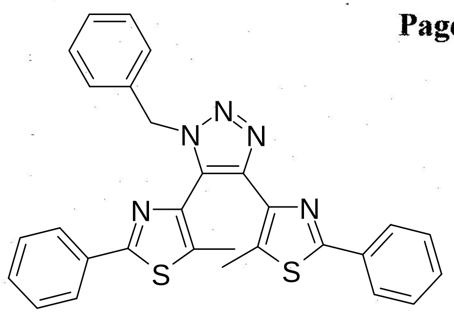

1o

Scan: 468
Base: m/z 477; 27%FS TIC: 2344704

R.T.: 5.3

#Ions: 400

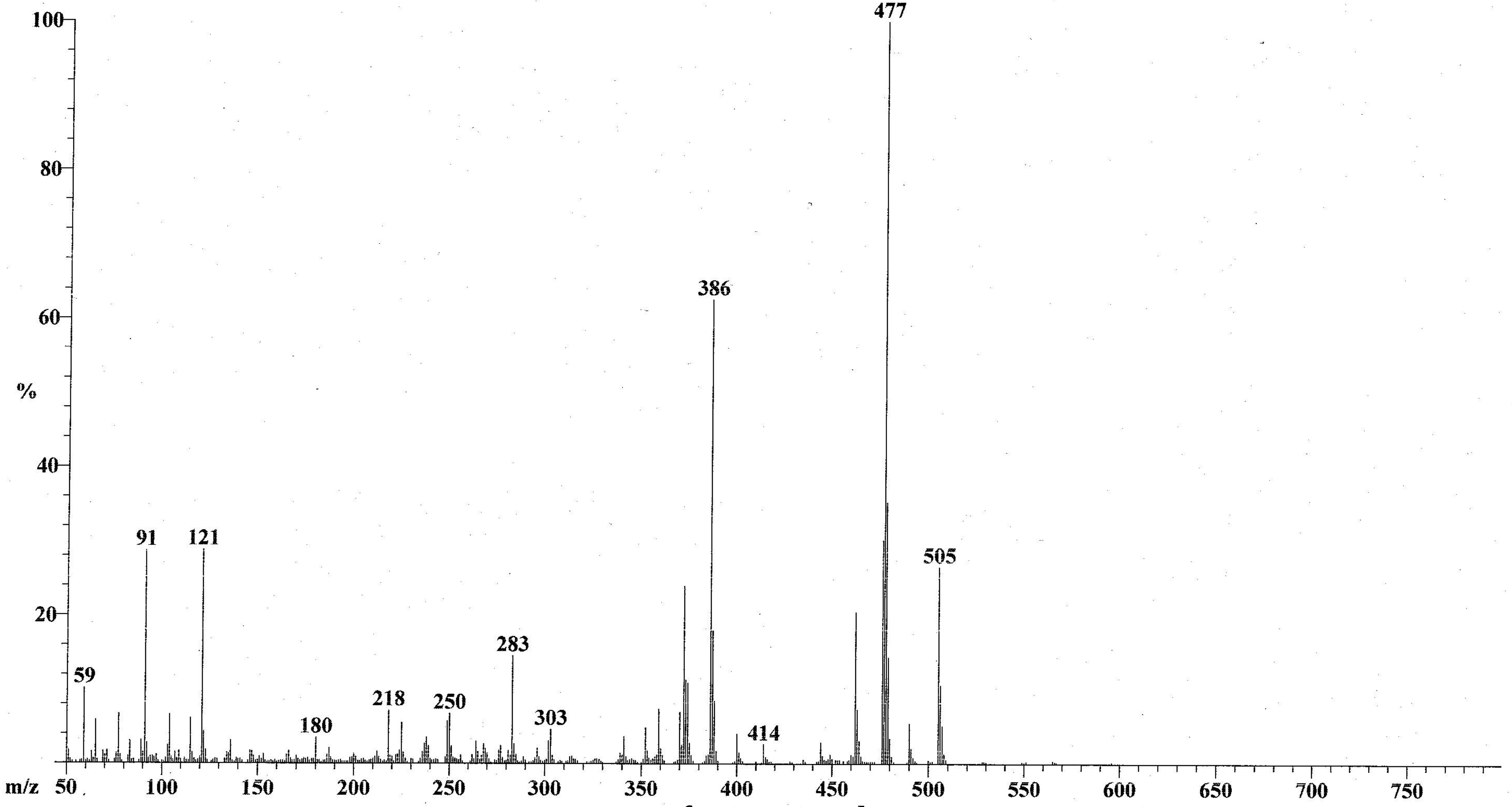

LRMS spectrum of compound **1o**

2010/01/12

File: YOK-kose135-2
Sample: Description
Instrument: JEOL MSRoute
Inlet: Direct Probe

Date Run: 2010-01-12 (Time Run: 16:59:03)

Ionization mode: EI+

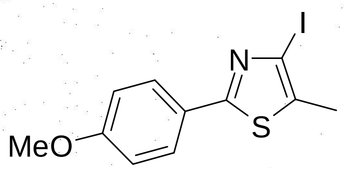

13

Scan: 70
Base: m/z 331; 48.4%FS TIC: 1317177

R.T.: 2.88

#Ions: 173

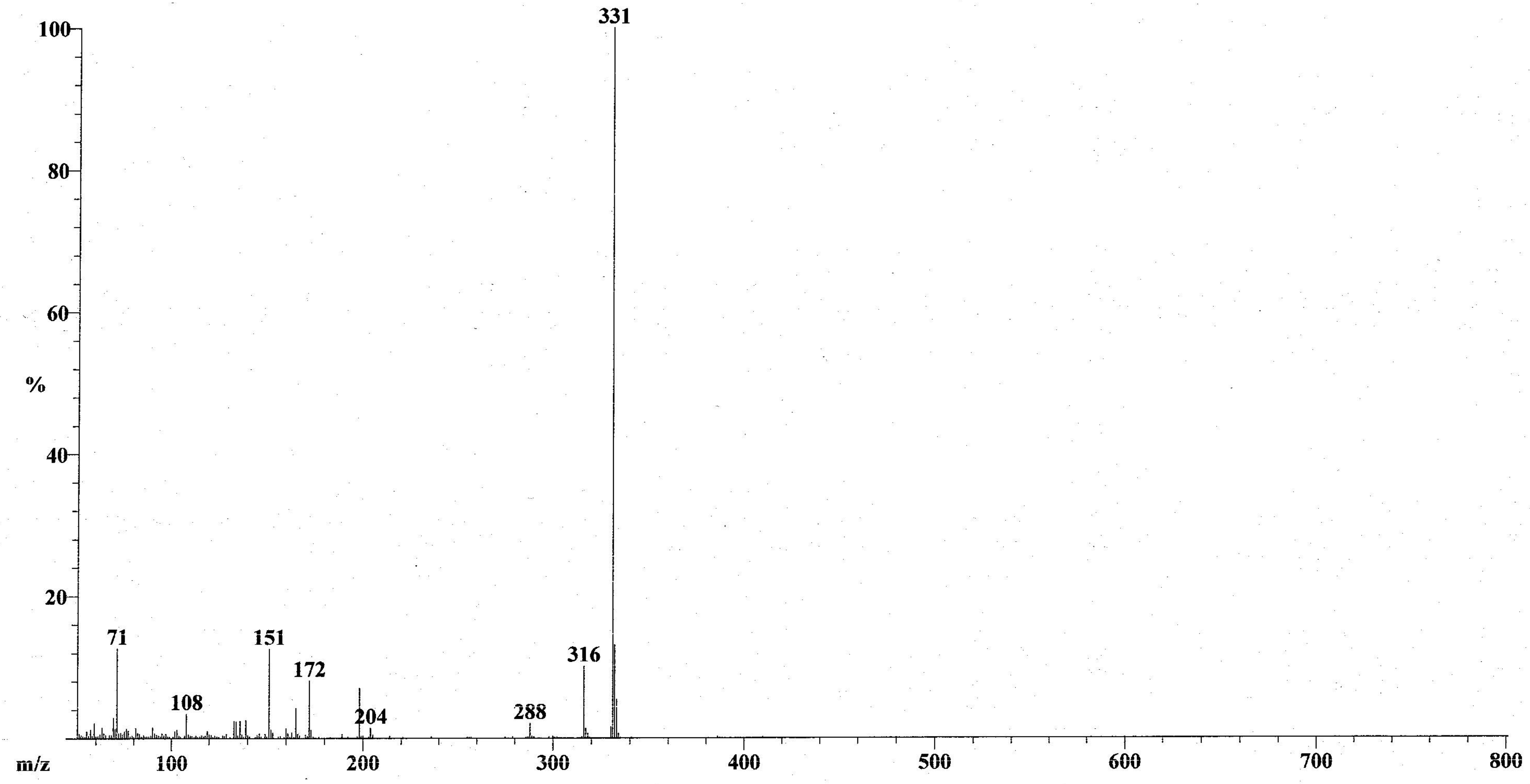

LRMS spectrum of compound **13**

2010/01/12

File: YOK-kose83-1
Sample: Description
Instrument: JEOL MSRoute
Inlet: Direct Probe

Date Run: 2010-01-12 (Time Run: 17:06:56)

Ionization mode: EI+

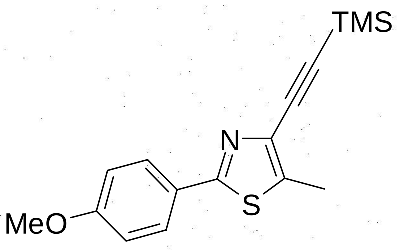

14

Scan: 63
Base: m/z 301; 67.2%FS TIC: 2782972

R.T.: 2.6

#Ions: 268

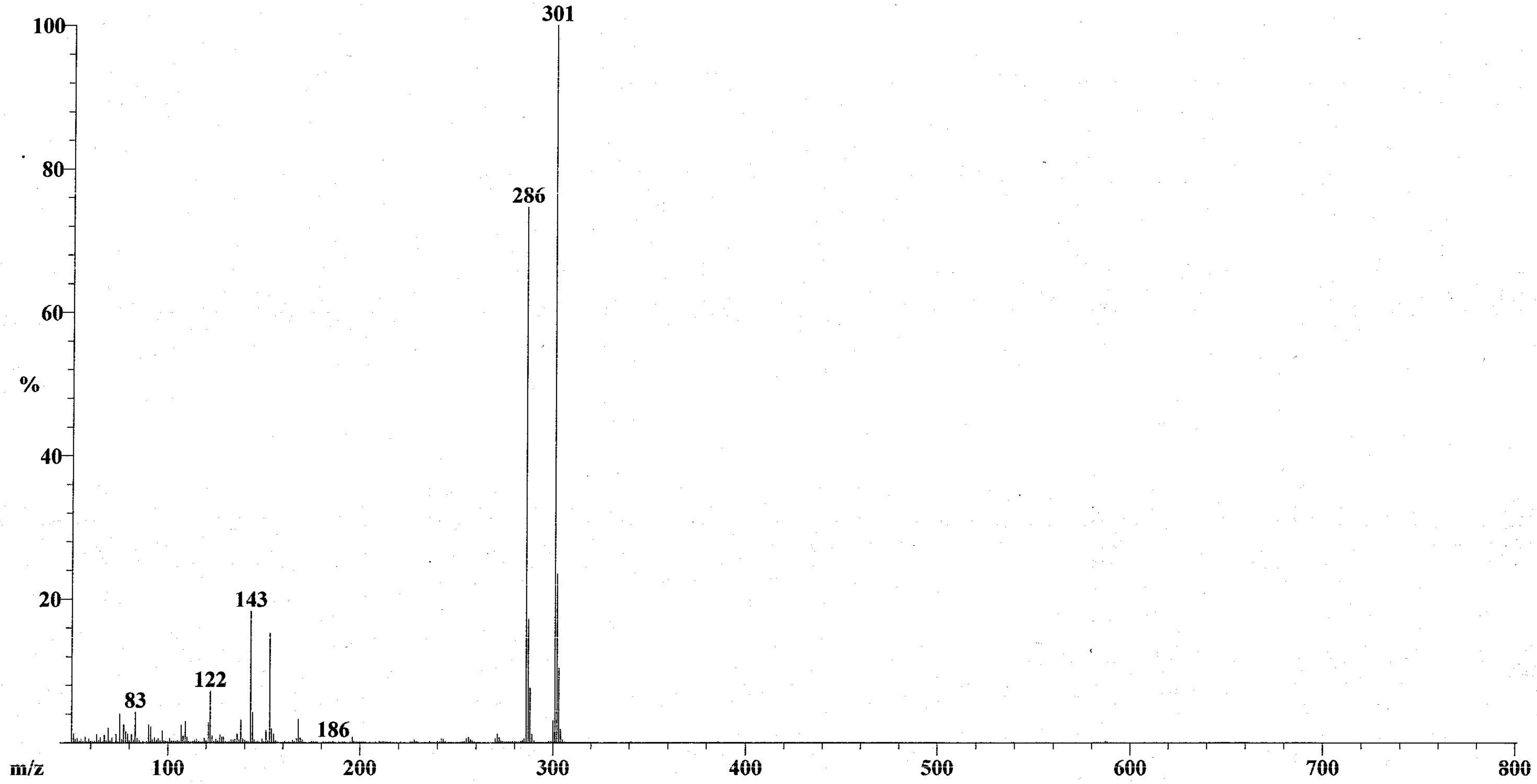

LRMS spectrum of compound **14**

File: YOK-kose65-1
Sample: Description
Instrument: JEOL MSRoute
Inlet: Direct Probe

Date Run: 2010-01-12 (Time Run: 17:14:27)

Ionization mode: EI+

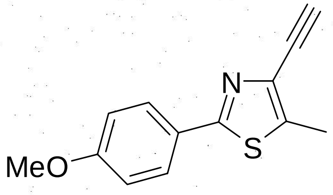

15

Scan: 62
Base: m/z 229; 65.7%FS TIC: 1830955

R.T.: 2.55

#Ions: 167

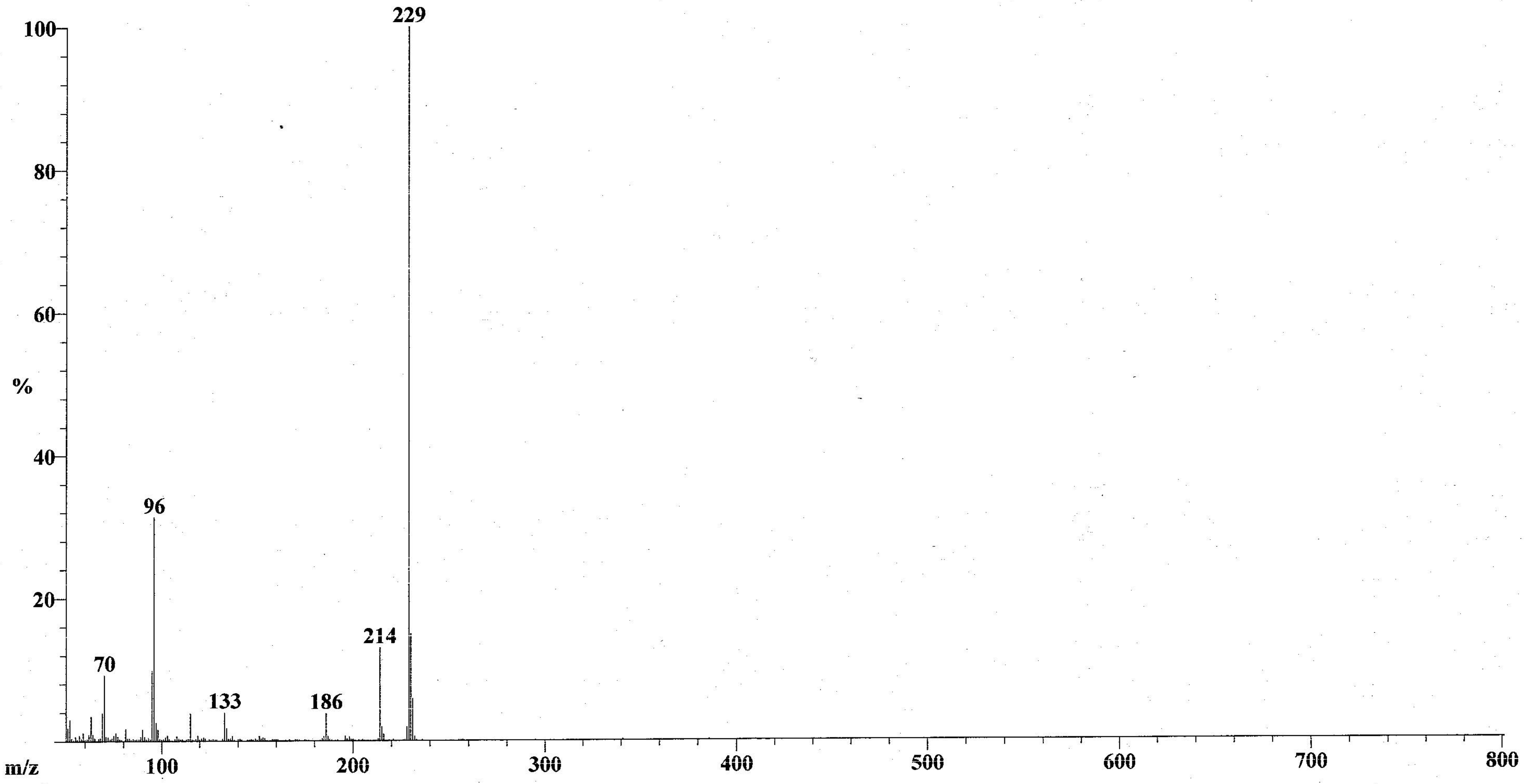

LRMS spectrum of compound **15**

2010/01/12 **Page 1**

File: YOK-kose155-1
Sample: Description
Instrument: JEOL MSRoute
Inlet: Direct Probe

Date Run: 2010-01-12 (Time Run: 17:20:58)

Ionization mode: EI+

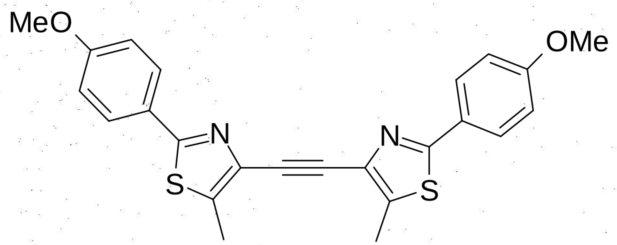

16

Scan: 98
Base: m/z 432; 46.9%FS TIC: 2344603

R.T.: 4.07

#Ions: 503

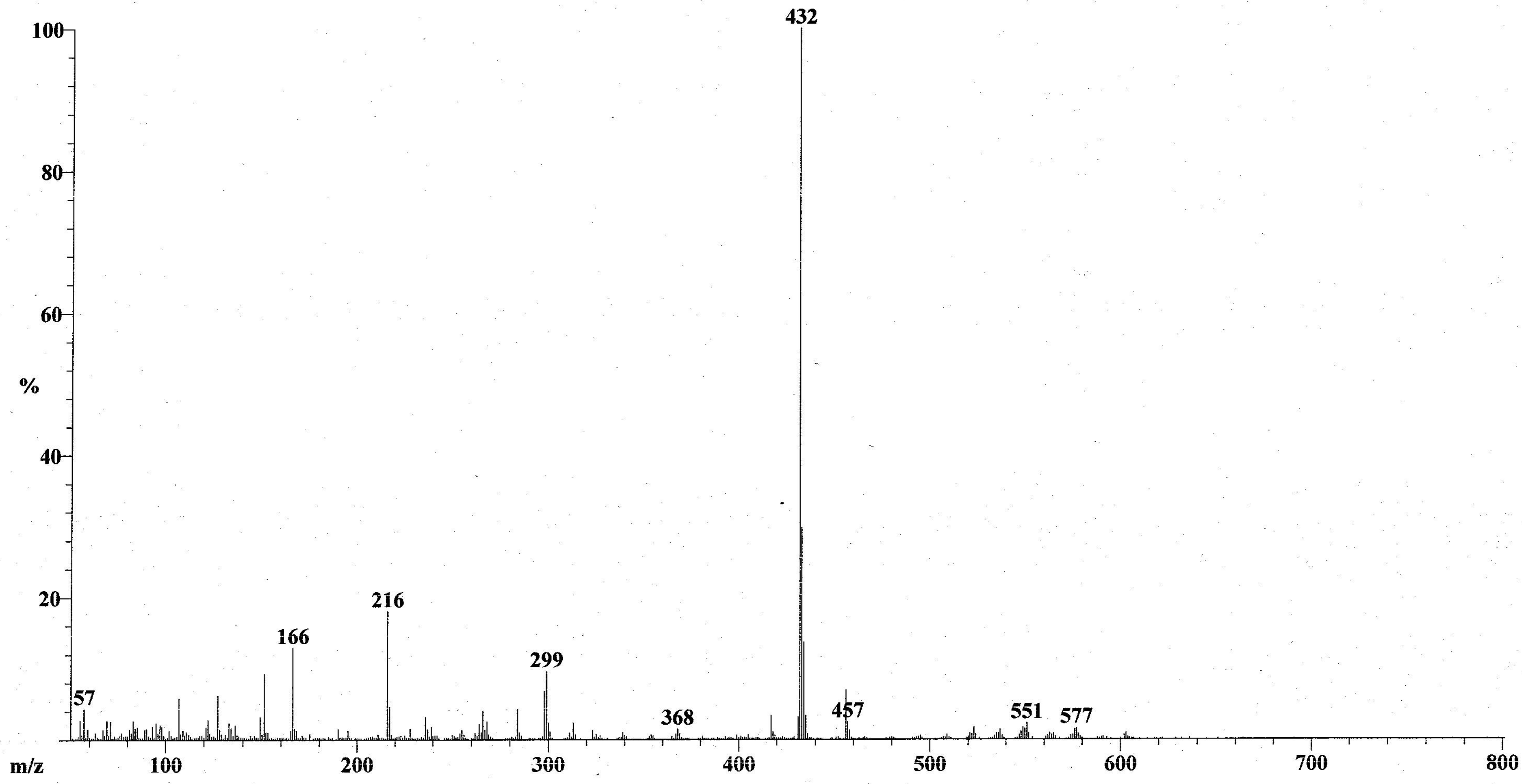

LRMS spectrum of compound **16**

2009/11/27

File: YOK-KOSE157-3-cry
Sample: Description
Instrument: JEOL MSRoute
Inlet: Direct Probe

Date Run: 2009-11-27 (Time Run: 10:08:51)

Ionization mode: EI+

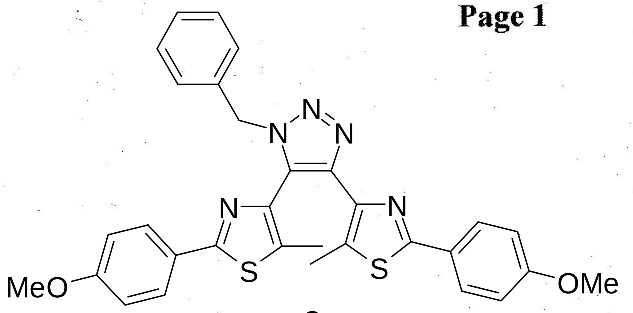

2o

Scan: 429
Base: m/z 537; 2.5%FS TIC: 210534

R.T.: 4.86

#Ions: 110

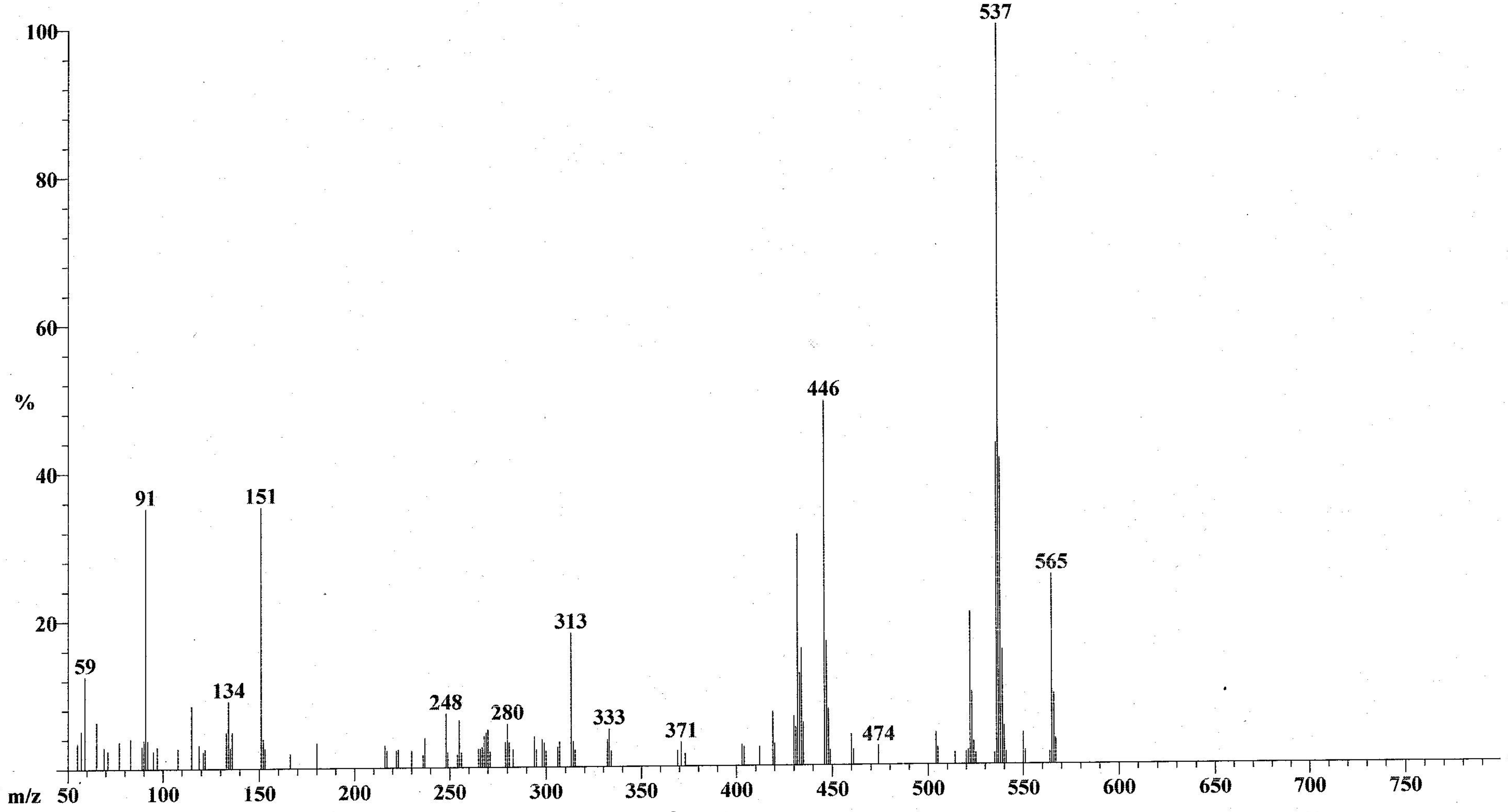

LRMS spectrum of compound **2o**

File: YOK-BOO-3-140-1
Sample: Description
Instrument: JEOL MSRoute
Inlet: Direct Probe

Date Run: 2009-12-15 (Time Run: 15:05:58)

Ionization mode: EI+

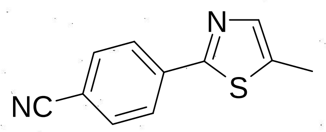

18

Scan: 30
Base: m/z 200; 2.1%FS TIC: 68654

R.T.: 1.23

#Ions: 22

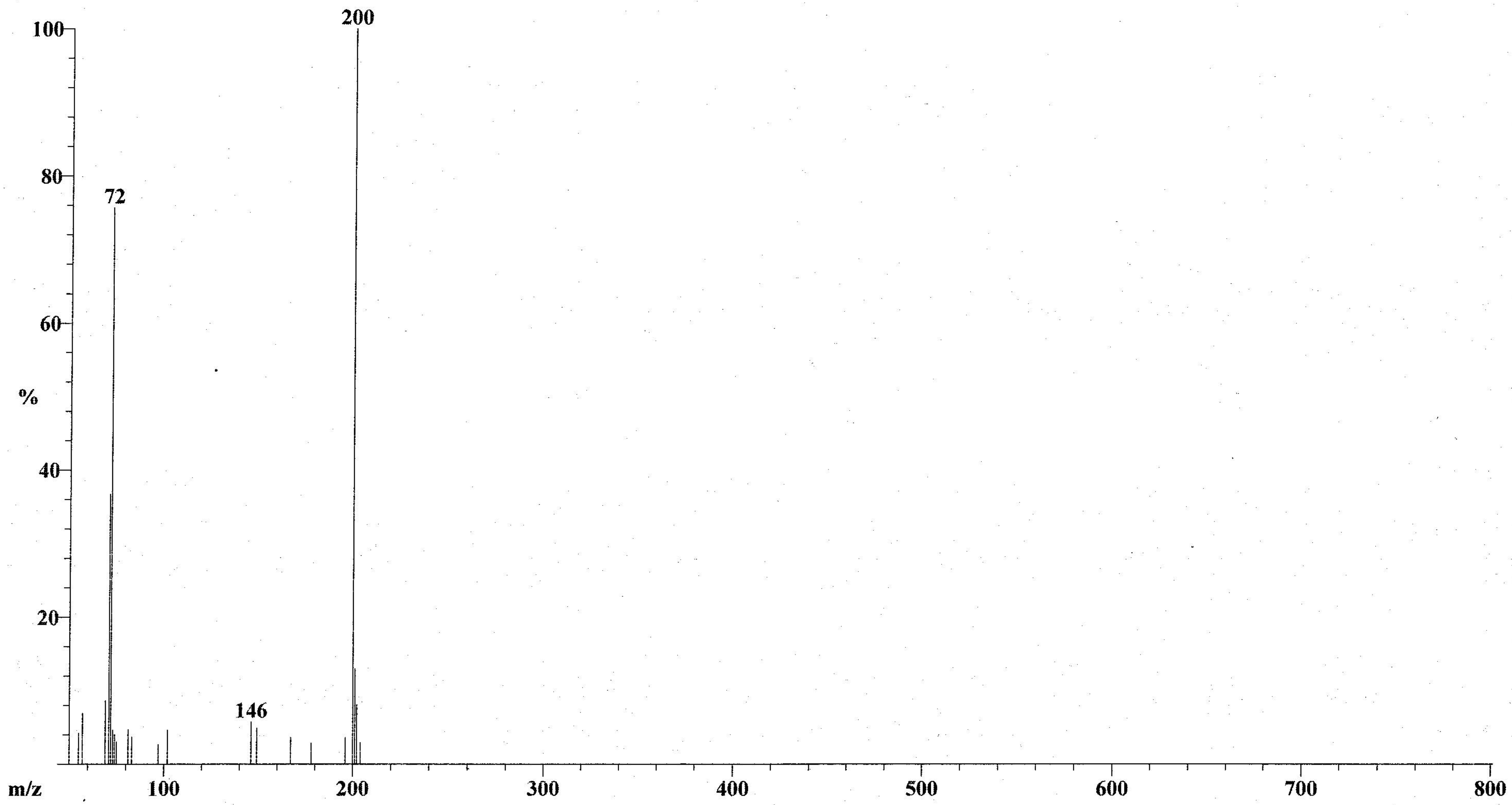

LRMS spectrum of compound **18**

2009/12/15

Page 1

File: YOK-BOO-3-144-3
Sample: Description
Instrument: JEOL MSRoute
Inlet: Direct Probe

Date Run: 2009-12-15 (Time Run: 15:52:46)

Ionization mode: EI+

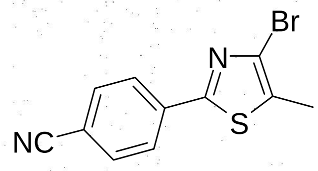

19

Scan: 68
Base: m/z 278; 5.5%FS TIC: 311118

R.T.: 2.8

#Ions: 70

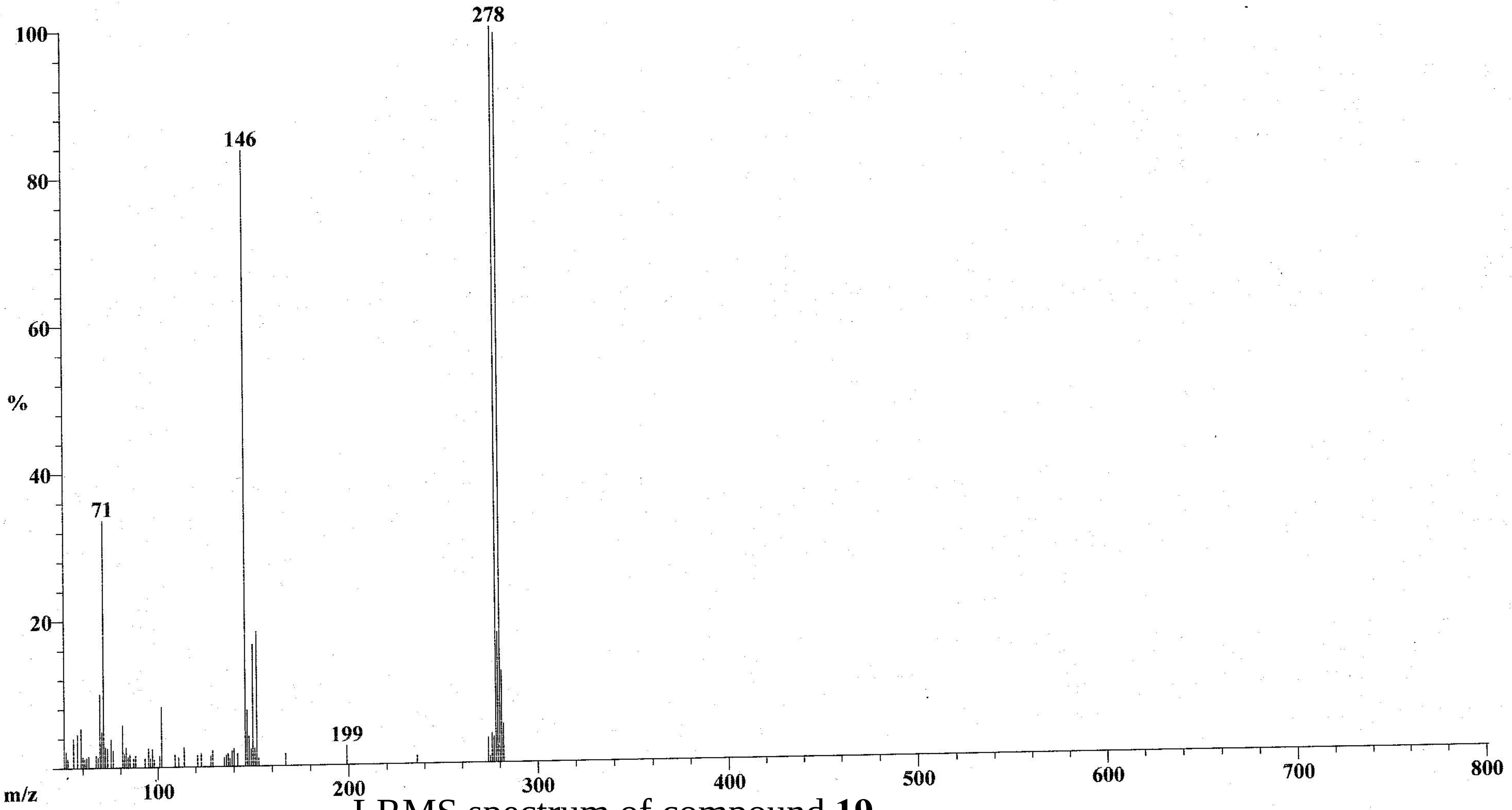

LRMS spectrum of compound **19**

2009/12/15

File: YOK-BOO-3-120-1
Sample: Description
Instrument: JEOL MSRoute
Inlet: Direct Probe

Date Run: 2009-12-15 (Time Run: 15:30:19)

Ionization mode: EI+

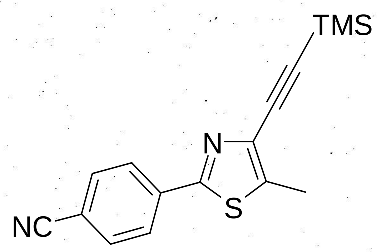

20

Scan: 59
Base: m/z 281; 14.1%FS TIC: 497482

R.T.: 2.43

#Ions: 98

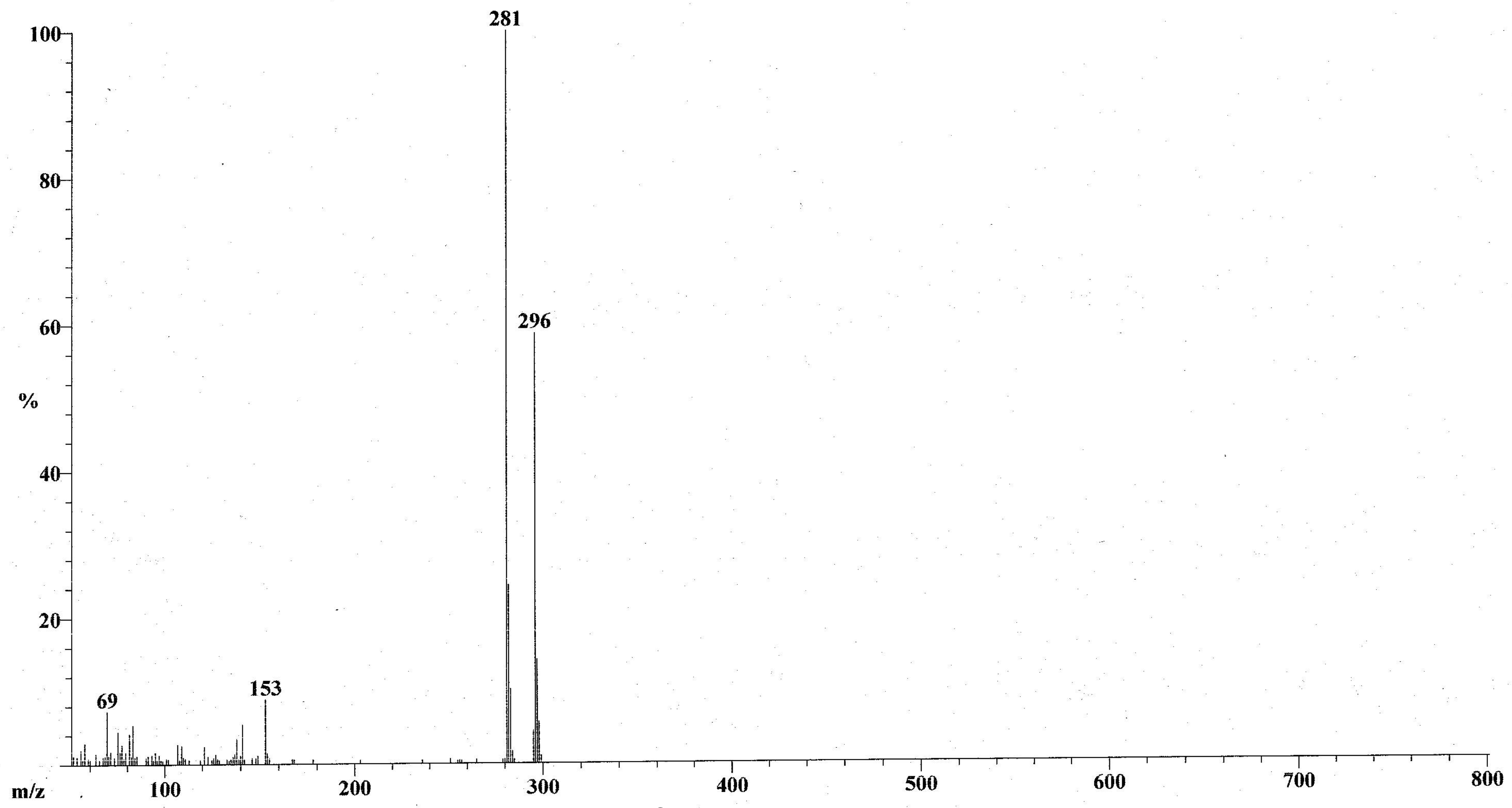

LRMS spectrum of compound **20**

2009/12/15

File: YOK-BOO-3-148-1
Sample: Description
Instrument: JEOL MSRoute
Inlet: Direct Probe

Date Run: 2009-12-15 (Time Run: 15:20:04)

Ionization mode: EI+

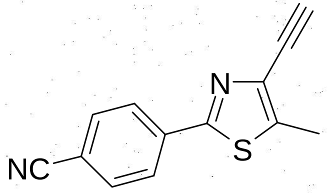

21

Scan: 54
Base: m/z 224; 1.2%FS TIC: 43028

R.T.: 2.23

#Ions: 20

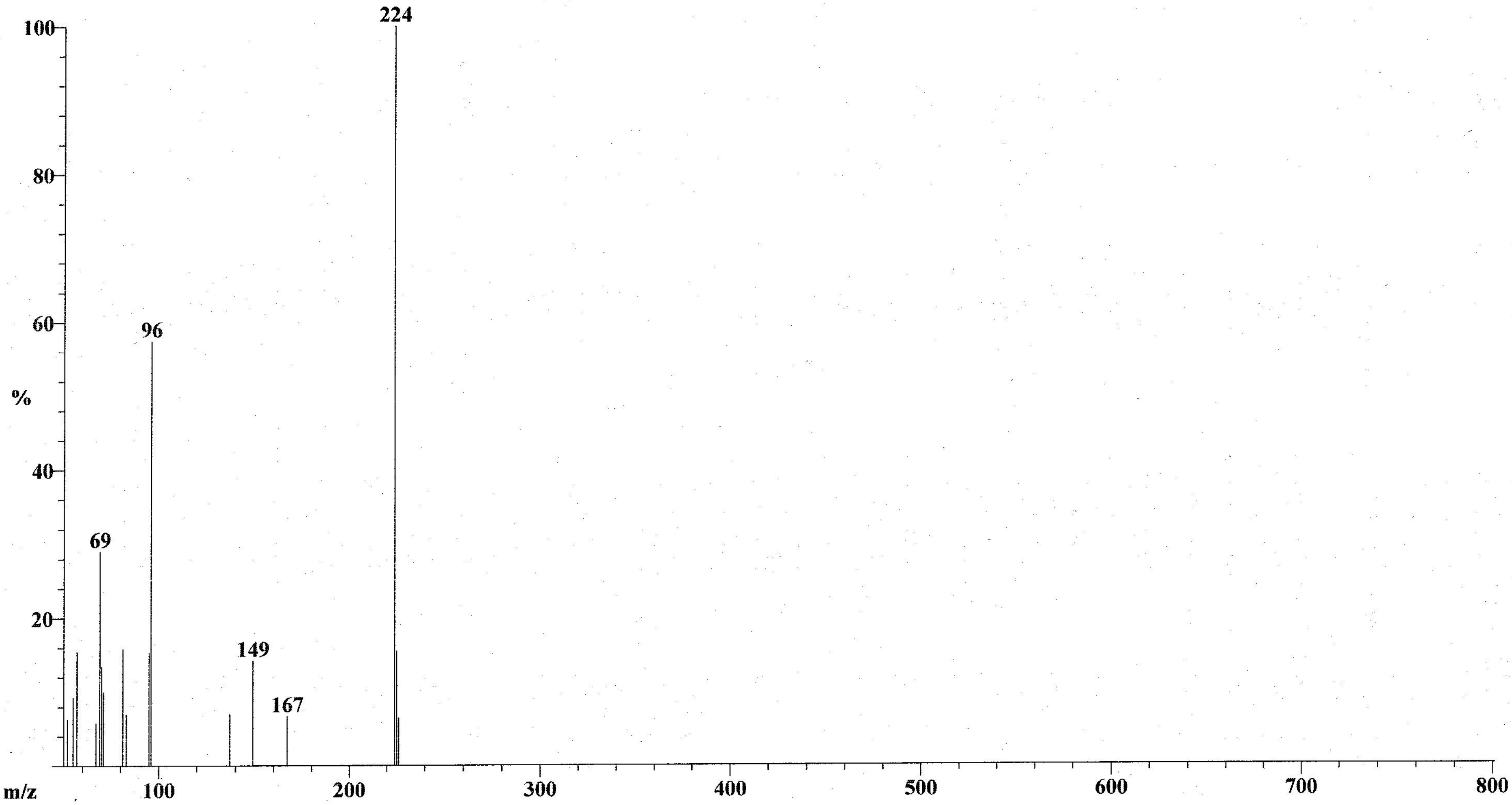

LRMS spectrum of compound **21**

2009/10/13

File: YOK-BOO-3-130-4
Sample: Description
Instrument: JEOL MSRoute
Inlet: Direct Probe

Date Run: 2009-10-13 (Time Run: 15:17:09)

Ionization mode: EI+

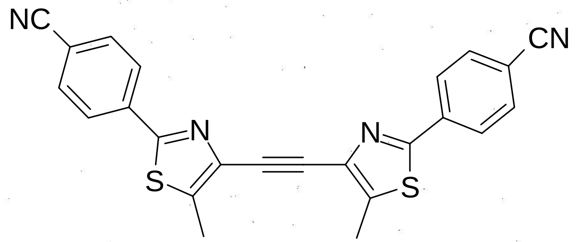

22

Scan: 367
Base: m/z 422; 13.2%FS TIC: 531353

R.T.: 4.15

#Ions: 205

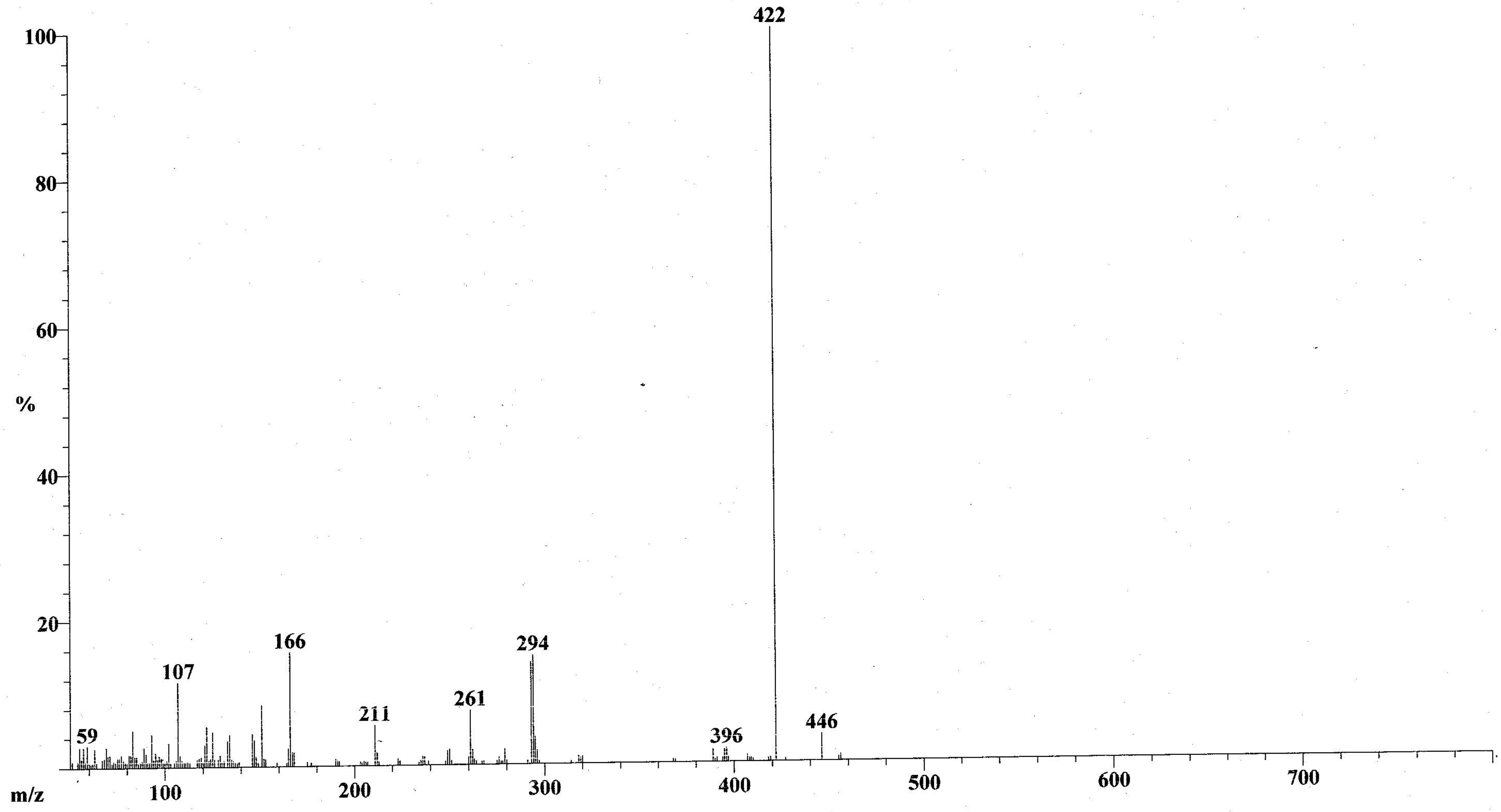

LRMS spectrum of compound **22**

File: YOK-BOO-3-1160-1 2nd
Sample: Description
Instrument: JEOL MSRoute
Inlet: Direct Probe

Date Run: 2009-11-27 (Time Run: 09:57:46)

Ionization mode: EI+

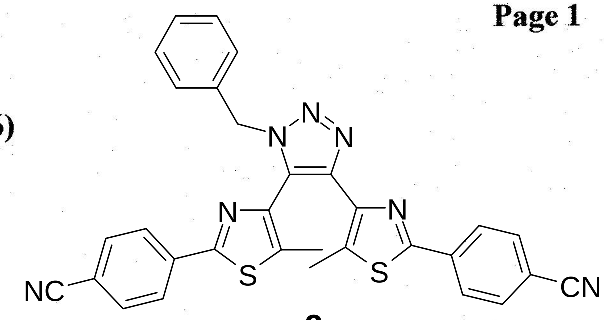

3o

Scan: 460
Base: m/z 91; 3.5%FS TIC: 278478

R.T.: 5.21

#Ions: 102

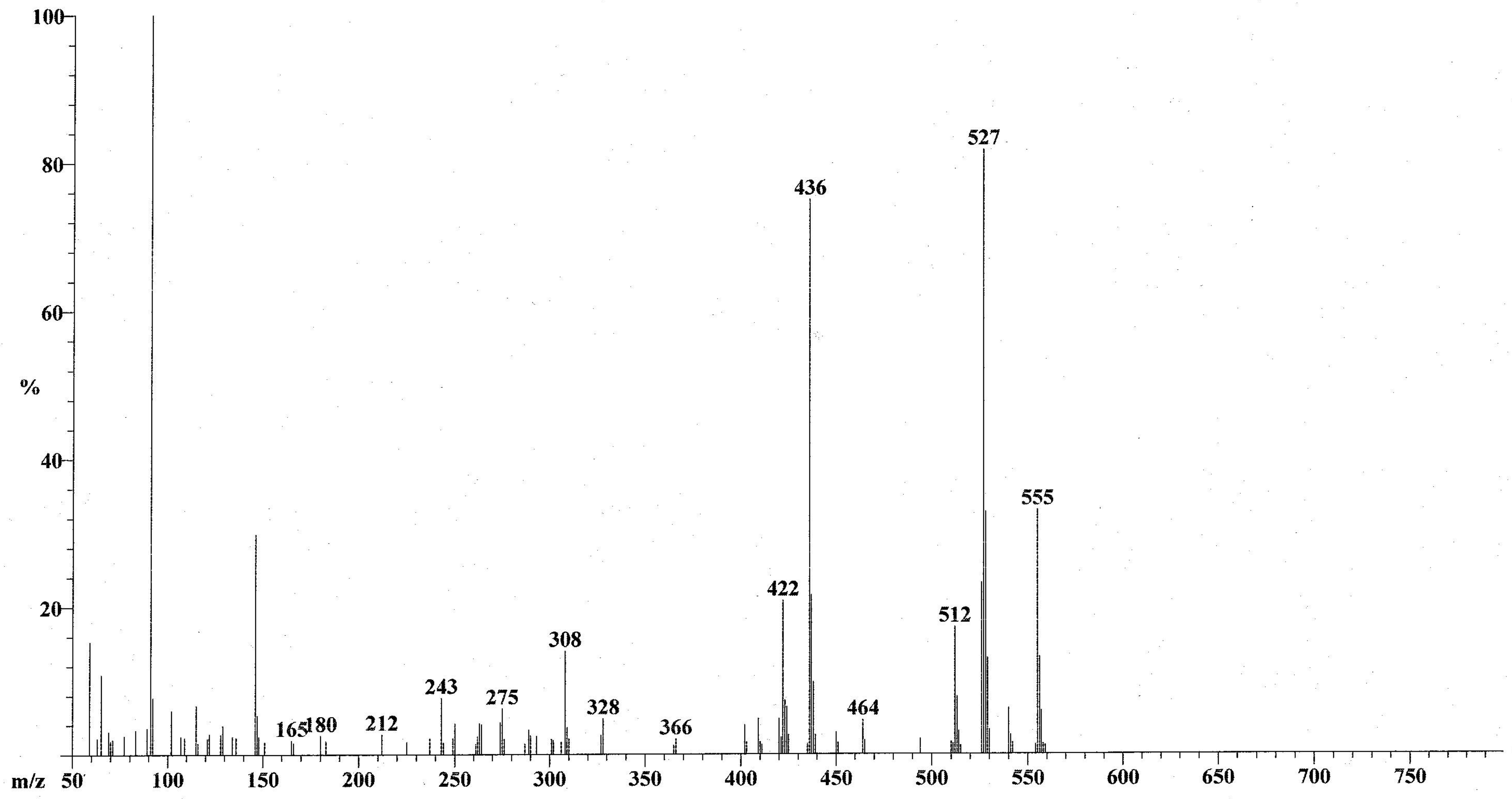

LRMS spectrum of compound **3o**
